# Ligand-induced activation of RyR1 in native membranes

**DOI:** 10.1038/s41467-026-75504-9

**Published:** 2026-09-24

**Authors:** Vasilii Mikirtumov, Sabrina Golusik, Ruifeng Huo, Thiemo Sprink, Nikita Balyschew, Wen Yang, Christoph Diebolder, Shuguang Yuan, Abhay Kotecha, Mikhail Kudryashev

**Affiliations:** 1 In situ Structural Biology, Max Delbrück Center for Molecular Medicine in the Helmholtz Association (MDC), Berlin, Germany; 2 Institute of Chemistry and Biochemistry, Freie Universität Berlin, Takustraße 6, Berlin, Germany; 3 Core Facility for cryo-EM, Charité-Universitätsmedizin Berlin, corporate member of Freie Universität Berlin and Humboldt Universität zu Berlin, Berlin, Germany; 4 Technology Platform for Cryo-EM, Max Delbrück Center for Molecular Medicine in the Helmholtz Association (MDC), Berlin, Germany; 5 Max Planck Institute of Biophysics, Frankfurt am Main, Germany; 6 Materials and Structural Analysis Division, Thermo Fisher Scientific, Eindhoven, The Netherlands; 7 AlphaMol Science Ltd, Shanghai, China; 8 Research Center for Medicinal Structural Biology, National Research Center for Translational Medicine at Shanghai, State Key Laboratory of Medical Genomics, Ruijin Hospital, Shanghai Jiao Tong University School of Medicine, Shanghai, 200025 China; 9 Institute of Medical Physics and Biophysics, Charité-Universitätsmedizin, Berlin, Germany; 10 Present Address: Institute of Medical Physics and Biophysics, Charité-Universitätsmedizin Berlin, corporatemember of Freie Universität Berlin and Humboldt Universität zu Berlin, Berlin, Germany; 11 Present Address: Center of Excellence for Structural Cell Biology, Department of Structural Biology, St. Jude Children’s Research Hospital, Memphis, TN USA

**Keywords:** Electron microscopy, Calcium channels, Membrane biophysics

## Abstract

Synchronized calcium release through arrays of the ryanodine receptor RyR1, fundamental to skeletal muscle excitation-contraction coupling, is achieved through the mechanical interaction of RyR1s and voltage-sensing receptors DHPR that activate RyR1s in response to action potentials. The calcium release is enhanced through “coupled gating”, when the activation of one channel promotes the opening of its neighbours. Here, we determine high-resolution structures of RyR1 in native sarcoplasmic reticulum membranes by cryo-EM/ET, capturing the conformations along the activation pathway and corner-to-corner interfaces between adjacent RyR1 receptors. Compared with purified RyR1s, receptors in native membranes follow an activation pathway with reduced cytosolic-shell tilt and greater consecutive in-plane rotation. Activation-induced rotation remodels the inter-receptor interface, lowering the energy barrier to the cooperative opening of the receptor cluster. Our analysis demonstrates how the native membrane receptor lattice influences ion channel cluster dynamics and provides a mechanistic framework for understanding calcium signaling in muscle.

## Abstract (web summary)

Effective muscle contraction relies on synchronized opening of RyR1 calcium channels. Here, authors present high-resolution structures of RyR1 in native membranes, revealing how ligand-induced activation of receptors leads to synchronous activation.

## Introduction

Skeletal muscle contraction relies on a precise interplay between electrical signals and Ca²⁺ release from internal stores^1^. This fundamental process, known as excitation–contraction, or EC-coupling, critically depends on the function of type-1 ryanodine receptors RyR1, large ion channels embedded in the sarcoplasmic reticulum (SR) membrane. Classic EM studies have shown that RyR1 tetramers interact with each other through corner-to-corner contacts, forming ordered arrays within the SR^2^. In these checkerboard-like arrays, every second RyR1 is mechanically coupled with a voltage-activated DHPR tetrad and can, upon activation, activate its neighboring DHPR-free RyR1s^3^. This phenomenon, known as coupled gating^3,4^, means that activation of one RyR1 can promote the opening of its neighbors, thereby synchronizing Ca^2+^ release and ensuring synchronous contraction of actin-myosin filaments. The structural basis for the physical communication between receptors is a central unanswered question in EC-coupling.

Disruptions in RyR1 function, whether due to genetic mutations, altered regulatory proteins, or disturbances in lipid composition, can lead to severe skeletal muscle disorders such as Malignant hyperthermia 1 (MHS1) and congenital myopathies 1 A and B (CMYO1A&B), highlighting the clinical significance of understanding RyR1 activation mechanisms^5^. The regulation of coupled gating is modulated by various factors, notably FKBP12, which is constantly bound to each RyR1 protomer with nanomolar affinity, stabilizing the channels’ closed state by imposing synchronous activity of the subunits^6,7^. Depletion of FKBP12 is known to induce aberrant channel activity, often resulting in calcium leak, as shown by single-channel recordings catching multiple subconductance states^8^. The knowledge of the activation mechanism of RyR1 comes from detailed high-resolution cryo-EM studies of purified RyR1 channels^9–16^. These studies have revealed substantial flexibility in RyR1’s large cytosolic domains, which undergo pronounced conformational changes, specifically large-scale tilting and outward rotation, accompanying pore opening^10,14,15^. However, purified RyR1 preparations lack critical physiological components, including the native lipid composition, endogenous regulatory proteins such as FKBP12, which is typically replaced by high-affinity isoform FKBP12.6 during purification, or is absent. The endogenous lipid environment plays a crucial role in modulating RyR1 function, influencing activation behavior and channel sensitivity^17,18^. Furthermore, direct interactions among neighboring RyR1 tetramers within the SR lattice have been proposed as critical determinants of coordinated gating^3,19^. But how native membrane conditions and inter-channel contacts specifically shape RyR1 gating transitions remains unclear. Advances in in situ cryo-electron tomography (cryo-ET) visualized RyR1 lattices within native SR membranes and muscle fibers^20–22^. Yet, the limited resolution of these structures and the availability of only the resting-state structures in most of these studies limit our understanding of detailed structural rearrangements during channel activation.

High-resolution structures from purified receptors defined the channel’s intrinsic response to ligands and lipids, while lower-resolution in situ studies demonstrated the overall arrangement of RyR1 in cells. An important unanswered question, however, is how these regulatory layers are integrated within the physiological context. To bridge this gap, we determine the structures of RyR1 by single-particle cryo-EM (SPA) and subtomogram averaging (StA) from cryo-ET data in its native membrane environment, within the receptor lattices, to define the structural basis of coupled gating. The RyR1 structures in four distinct as well as two intermediate functional states induced by activating ligands show that the activation of RyR1 includes a large rotation component in the membrane plane. The structures reveal direct, corner-to-corner physical contacts between receptors, providing a high-resolution view of the interface that mediates coupled gating. We show that these lattice interactions constrain the channel’s activation pathway to an in-plane rotation-dominant motion and that the interfaces are fine-tuned to facilitate cooperative activation. These findings explain the mechanism of ligand-gated activation of RyR1 in native membranes and provide structural insights critical for understanding RyR1-associated skeletal muscle diseases and developing targeted therapeutic strategies.

## Results

### Structures of the RyR1 channel in native membranes

**Fig. 1:**
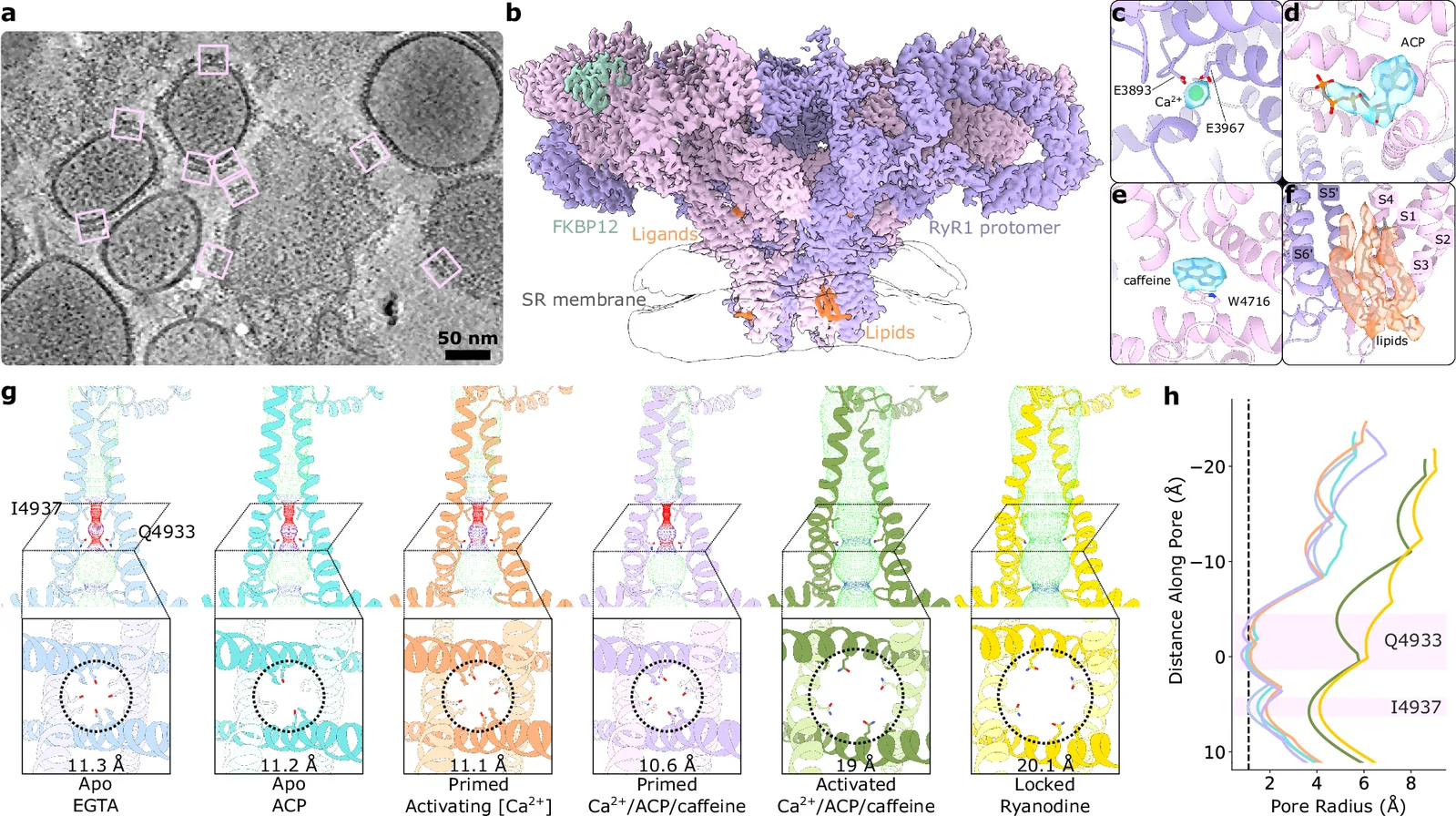
High-resolution structures of RyR1 in native membranes reveal ligand binding and pore conformations across the activation pathway. **a** A representative slice through a cryo-electron tomogram of SR vesicles, showing arrays of RyR1 channels, highlighted with squares. **b** Side view of the composite cryo-EM map of the Primed state, derived from the ActMix dataset. Protomers are individually colored, with the associated FKBP12 shown in cyan, ordered lipids and ligands in orange, and the SR membrane in gray. **c–e** Detailed views of the ligand-binding sites from the high-resolution map in (**b**), showing densities for: **c** a high-affinity Ca²⁺ ion, **d** the non-hydrolyzable ATP analog ACP, **e** caffeine. **f** Ordered lipid densities resolved within a hydrophobic pocket formed at the domain-swapped interface between transmembrane domains of adjacent subunits. The prime symbol (‘) denotes helices from the neighboring subunit. **g** Conformational changes of the ion-conducting pore across the full activation pathway. Side and top views of the pore are shown for the Apo (EGTA-ET, ACP), Primed (Ca²⁺, ActMix), Activated (ActMix), and Locked (Ryanodine) states. Side chains of the key gating residues I4937 and Q4933 are depicted as sticks. The pore interior is colored by radius: constricted (<1.1 Å, red), partially open (1.1–2.2 Å, blue), and fully open ( > 4 Å, green). **h** Pore radius profiles corresponding to the structures shown in (**g**). The minimal radius of a hydrated Ca^2+^ ion of 1.1 Å is indicated by the dashed line, illustrating the transition from impermeable to permeable states. Highlighted regions correspond to the locations of the primary (I4937) and secondary (Q4933) gates.

We isolated junctional SR microsomes from rabbit skeletal muscle and examined RyR1 arrays by cryo-ET (Fig. 1a), following the previous reports^20,21,23,24^ with modifications, and performed high-resolution imaging of the SR vesicles in 3D by cryo-ET and in 2D for single-particle cryo-EM. While the linear two-row organization of the receptors in the muscle was perturbed, the local order was preserved with the corner-to-corner contacts clearly visible (Supplementary Fig. 1). The corner-to-corner contacts were formed via bridging-solenoid BSol domains, recapitulating the physiological arrangement of RyR1. Using a combination of SPA and StA, we determined multiple RyR1 structures under the following conditions: resting (Apo) states with and without the non-hydrolyzable ATP analog adenylyl-imidodiphosphate, denoted ACP; a Primed state induced by activating [free Ca^2+^] (ActCa); a mixture of Primed and Activated states in equilibrium by the addition of Ca^2+^, ACP, and caffeine (ActMix); and a Ryanodine-locked state with a fully dilated pore. The reconstructions had global resolutions between 3.5 Å and 4.9 Å (Supplementary Table 1). Focused refinement further improved local resolutions up to 3.0 Å in the most ordered regions, enabling building atomic models (Fig. 1b, Supplementary Figs. 2–10) and confident placement of side chains, bound ligands, and ordered lipids (Fig. 1c–f).

Structural analysis revealed ligand densities that matched previously identified binding sites: Ca^2+^ occupied high-affinity site coordinated by residues E3893 and E3967 side chains (Fig. 1c), and ACP was bound within the known nucleotide pocket formed by S6c, CTD, and TaF motif^25^ (Fig. 1d). Caffeine density appeared stacked with the TRP4716 indole ring of the S2S3 helical bundle (Fig. 1e). Density corresponding to the ryanodine moiety was bound near Q4933 occluding the pore and locking the channel open. Endogenous FKBP12 remained bound in all states, confirming the native composition of the interacting partners of RyR1. The juxtamembrane helices at the top of the TMD interface make several contacts with the SR membrane and are possibly modulating the local curvature and flattening of the SR membrane around the TMD, similarly to the previously reported structures of RyR1 in proteoliposomes^26^. We observed well-resolved lipid densities nestled in the hydrophobic pocket between the pVSD (helices S1–S4) and the Pore domain (helices S5’–S6’) of the other subunit (Fig. 1f), similar to those identified previously^15,16,18^. Ordered lipids were present in both the closed and open-state maps, where local resolution reached 3–3.2 Å, whereas previous high-resolution studies on purified RyR1 reconstituted in nanodiscs reported the absence of lipids in the open-pore state due to the steric clash with Phe4808^15^. The previously reported transmembrane helix S0^14,27^ was undetectable in native membranes, suggesting partial order or limited functional significance in the membrane context. Interestingly, we haven’t observed any co-purified ATP molecules in any of the EGTA datasets, although ATP is expected to be constantly bound in the skeletal muscle due to its high cytosolic concentration and cannot be hydrolyzed by RyR1^14,28^.

Our analysis further revealed conformational variability of RyR1 cytosolic shell in native membranes, particularly in the Apo state, classifiable into discrete “Up,” “Down,” and “Tight” states based on positions of BSol domains (Supplementary Fig. 11). The “Down” state featured a shell closer to the membrane, “Up” displayed elevated orientation, and “Tight” exhibited BSol domains positioned closer to the channel axis; domain rotations were in the order of 1–2 degrees. Such variability likely represents receptor flexibility in the absence of ligands. Cytoplasmic shells exhibit a wide range of motion in all states of the receptor; therefore, for each of the states in this study, we obtained additional classes, typically resulting in the shell position being either “Up” or “Down”, termed relative to the consensus reconstruction for each of the functional states (Supplementary Table 1). In all datasets, the consensus reconstructions from all particles represented the major class (Supplementary Figs. 12, 13). The “Down” conformation was predominant in the ActCa dataset (85.4% of particles, Supplementary Table 1), while the activating ligands have shifted particle populations predominantly to the “Up” conformation: 72.5% and 56.7% of particles for the Primed and Activated states, respectively (Supplementary Table 1). The ActMix-ET dataset yielded both “Down” and “Up” conformations in addition to the consensus reconstruction, while 67% of particles were “Up” in the Ryanodine dataset (Supplementary Table 1).

Unlike other datasets where the EF1&2 hand domain remains stable across subclasses, the EGTA-ET dataset revealed significant subclass-specific conformational variability in this region (Supplementary Fig. 14). Structural similarity analysis of the EF1&2 hand domain using multidimensional scaling (see “Methods”) relative to the CSol domain (Supplementary Fig. 14i) or the neighbouring S2S3′ bundle (Supplementary Fig. 15j) consistently placed all EGTA-ET models outside the established Apo cluster, closer to canonical activated and inactivated states (Supplementary Fig. 15a–h, j). Because inactivated conformations are reported to form salt bridges across this interface (Glu4075–Arg4736 and Lys4101–Asp4730)^15^, we measured the corresponding Cα-Cα distances. This also showed a higher similarity of the EF1&2 hand domain in the Apo-states in membranes to the canonical open and inactivated states of purified receptors (Supplementary Fig. 15i).

### Pore opening in native membranes

Our analysis of the RyR1 transmembrane domain in native membranes revealed distinct conformational changes compared to the purified channels. In one of the Apo states (Apo1/EGTA-ET), we observed dual constriction: the primary hydrophobic gate was at residue I4937, while a second constriction was seen near residue Q4933 on the pore-lining S6 helix (Fig. 1g, h). This dual-gate arrangement was previously reported only in the nanodisc-reconstituted inactivated structure^15^. Ryanodine treatment resulted in uniformly open channels with a 20 Å aperture (pore diameter measured at the I4937 Cα level), validating ligand-induced pore dilation structurally. RyR1s exposed either only to Ca^2^⁺ or ACP alone remained consistently closed, in line with the purified channel data^14^.

Upon activation by “ActMix”, we observed a substantial fraction of RyR1 (~35%) channels in the open state with fully dilated pores (19 Å aperture at the I4937 Cα level). This large aperture surpasses openings recorded from most of the purified RyR1^18^, suggesting that native membrane conditions enhance pore expansion. Classification of particles from this state revealed four distinct conformations, each with all ligands bound; two of these structures showed dilated pores, consistent with open-channel states, and thus we classified these as “Activated”. In contrast, the remaining two structures exhibited closed pores of ~11 Å, and we refer to these as “Primed”. However, given the minor conformational differences observed between the two closed-pore states, it is impossible to identify whether they represent a ready-to-open primed state or an inactivated conformation. A potentially inactivated state could represent a temporary refractory condition following ligand binding, highlighting the structural complexity of RyR1 in native membranes.

### RyR1 activation is driven by in-plane rotation of the cytosolic shell

**Fig. 2:**
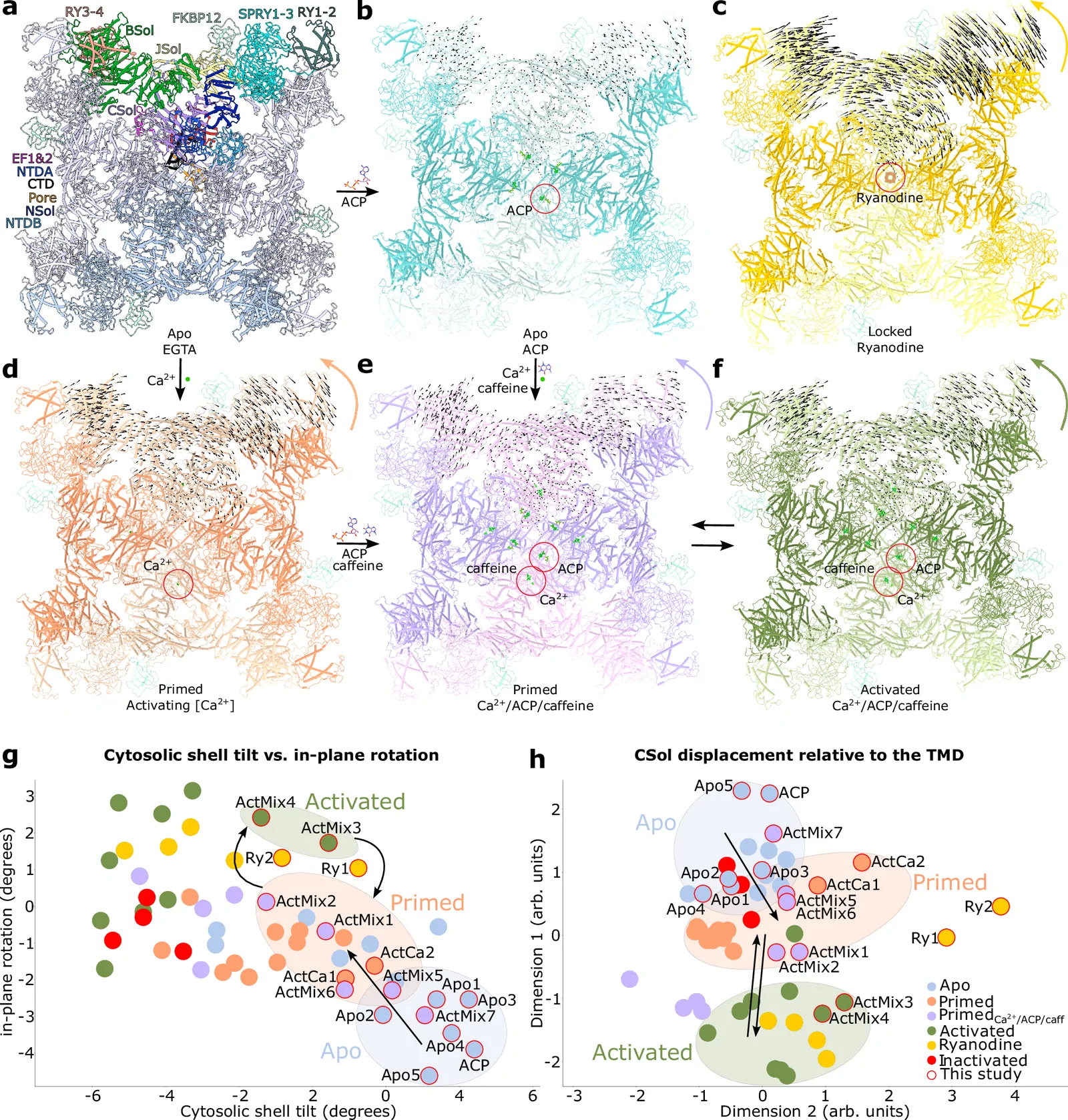
Activation of RyR1 in native membranes is dominated by in-plane rotation of the cytosolic shells. **a** Top-down view of the Apo-state model from the EGTA-SPA dataset, with major cytosolic and other domains labeled. **b–f** Visualizations of conformational transitions between key functional states, shown from a top-down perspective. Vectors on a single protomer indicate the direction and magnitude of domain displacement for each transition. **b** Transition from the Apo state (EGTA-SPA) to the ACP-bound Apo state. **c** Global change from the Apo state (EGTA-ET) to the Ryanodine-Locked state. **d** Initial priming from the Apo state (EGTA-ET) to the Ca²⁺-bound Primed state. **e** Further priming, showing the transition from the Ca²⁺-only primed state to the Primed state in the full activation mixture (ActMix). **f** The final gating step, showing the transition from the Primed state to the Activated (open) state, both derived from the ActMix dataset. **g** Quantitative comparison of cytosolic assembly motion across all available RyR1 structures. This plot of cytosolic shell tilt versus in-plane rotation demonstrates that structures from native membranes (circled in red) follow a distinct, rotation-dominant activation pathway. This contrasts with the large tilting motion observed in key purified receptor structures. Models are coloured by functional state. See Supplementary Fig. 24a for a complete list of structures. **h** Multidimensional scaling plot of conformational changes within the activation core (CSol domain displacement relative to the TMD). The clustering shows that core rearrangements are similar between native (circled in red) and purified RyR1 structures. See Supplementary Fig. 24b for a complete list of structures.

Previous studies on purified RyR1 channels proposed an activation mechanism involving significant vertical tilting of the cytosolic shell^10,14^ toward the SR membrane and in-plane rotation around the channel’s central axis, the former defined as the flexion angle in previous reports^15,29^. Calculations based on the reported structures showed that the activation was associated with an extensive ~5.7° downward tilt and a ~ 5.1° rotation^14^. Our analysis of RyR1 structures in native membranes revealed a significantly lower total downward tilt of ~2.8°, accompanied by a higher cumulative in-plane rotation of ~6.3° during activation (Figs. 2a–f, Supplementary Figs. 16, 17). In native membranes, the activation sequence progresses from Apo (EGTA/ACP) through Primed (activating [free Ca^2+^] and ActMix conditions) to Activated (ActMix) states. During priming, the RyR1 cytosolic shell tilts by ~2.8° and rotates by ~3.9° (Supplementary Fig. 17b, c), while full activation is characterized primarily by additional rotation of ~2.4° without further significant tilt (Supplementary Fig. 17d). In contrast, purified RyR1 structures exhibit greater overall conformational change with a cumulative tilting of ~4.5° during priming, and an additional ~1.1° tilt upon activation^14^. The total difference in cumulative tilting between purified and native RyR1 channels is ~3°, emphasizing that native environment constraints limit the vertical domain movements (Fig. 2g). Comparison of the structures of RyR1 in SR membranes and the purified receptors quantified by a per-domain RMSD calculation confirms this trend (Supplementary Figs. 18–21).

This major difference in motion suggests that the physical packing of receptors within the native SR lattice sterically hinders large-scale tilting movements, thereby funneling the conformational transitions of activation into a rotational motion. This hypothesis is supported by multidimensional scaling analysis, which shows that the activation core conformations relative to the transmembrane domain are similar between purified and native structures (Fig. 2h), while the peripheral cytosolic domains are significantly different (Fig. 2g). The shell in the SR structures is significantly higher/tilted up in all functional states, both on the absolute scale (Supplementary Fig. 16d, e) and during functional state transitions (Supplementary Fig. 17a–d). As the proteoliposome- or nanodisc-reconstituted structures^15,26^ do not exhibit the same trend, this observation cannot be explained by the presence of the SR membrane alone. We therefore suggest that the combination of the native interacting partners, including FKBP12 and the lateral contacts with neighboring RyR1 tetramers in the native SR lattice, restricts large-scale tilting of the cytoplasmic shell during activation. In turn, activation-induced rearrangements within the TMD and activation core appear to be absorbed in the SR by intra-domain deformations of the BSol itself rather than by a rigid-body tilt of the whole shell (Supplementary Fig. 22a–d, 23).

### A BSol-mediated interface forms the structural basis for RyR1 coupling

**Fig. 3:**
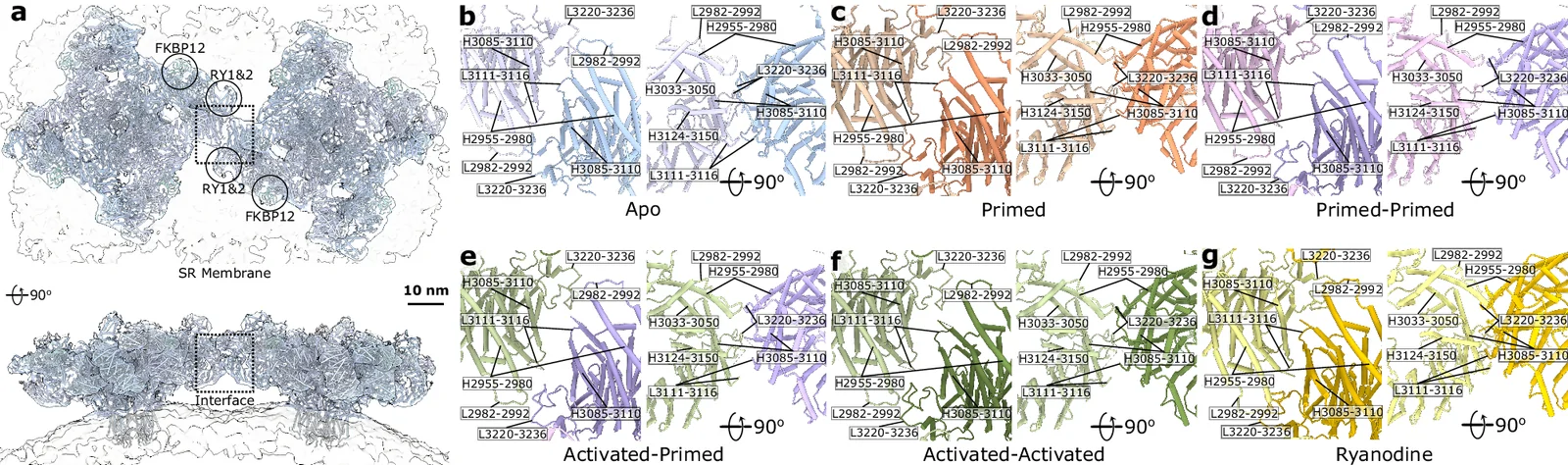
Structural characterisation of the RyR1-RyR1 interaction within the arrays via BSol-mediated interfaces in native membranes. **a** Top and side views of the Apo-state RyR1-RyR1 dimer map from the EGTA-ET dataset, with corresponding atomic models rigid-body fitted into the density. **b–g** Close-up views of the BSol-mediated corner-to-corner interface across the activation pathway. The region shown is indicated by the dashed box in (**a**). Interfaces are displayed for the: **b** Apo-Apo dimer (EGTA-ET dataset), **c** Primed-Primed dimer (Activating [Ca²⁺] dataset), **d** Primed-Primed dimer (ActMix dataset), **e** Primed-Activated dimer (ActMix dataset), **f** Activated-Activated dimer (ActMix dataset), **g** Locked-Locked dimer (Ryanodine dataset).

To understand the mechanism constraining the channel’s tilt and mediating inter-receptor communication, we analyzed the interactions between adjacent receptors by determining the structures of RyR1-RyR1 dimers in five different states (Fig. 3, Supplementary Fig. 27 and Supplementary Table 1). For this, we took advantage of the local order of the RyR1-RyR1 lattice (Supplementary Fig. 1). The resolutions of the dimer maps ranged from 11.7 to 26 Å (Supplementary Table 1), limited by the particle number and the conformational heterogeneity of the flexible interface. As these structures are the product of averaging, our reconstructions specifically represent the most homogeneous and well-ordered lattice units, as the more flexible dimer arrangements were excluded during 3D classification. Ordered contact preservation was particularly high in the SPA datasets, reaching 89% in the ActCa dataset (Supplementary Table 1). In cryo-ET datasets, the estimated contact ratio was lower; we analyzed 3D neighbour heatmaps demonstrating the preferred arrangements of neighbours (Supplementary Figs. 28a, 29a). These heatmaps showed four C4-symmetry-related peaks corresponding to native corner-to-corner interactions. Notably, a fraction of RyR1 particles were lost during cryo-ET data processing due to technical limitations, resulting in only a fraction of channels being engaged in the interaction with neighbors in the case of EGTA-ET and ActMix-ET datasets. The Apo-state dimer structure resembled the in situ dimer structures also in the Apo-state from FIB-milled native triad junctions^22^ (EMD-37094). The Inter-receptor center-to-center distance was measured at 31.5 nm, closely matching the 31 nm average seen in intact triads (Supplementary Fig. 30). Similarly, the center-to-center angle was 17°, aligning with the native 18.3° angle. This analysis shows that the RyR1 lattice tends to be locally preserved in purified SR microsomes even in the absence of the T-Tubule membrane. Our dimer maps allowed us to unambiguously dock high-resolution monomer models from the same datasets, revealing clear corner-to-corner interactions mediated primarily by the BSol domain loops (residues 2982–2992, 3111–3116, 3220–3236) and helices (residues 2955–2980, 3085–3110).

**Fig. 4:**
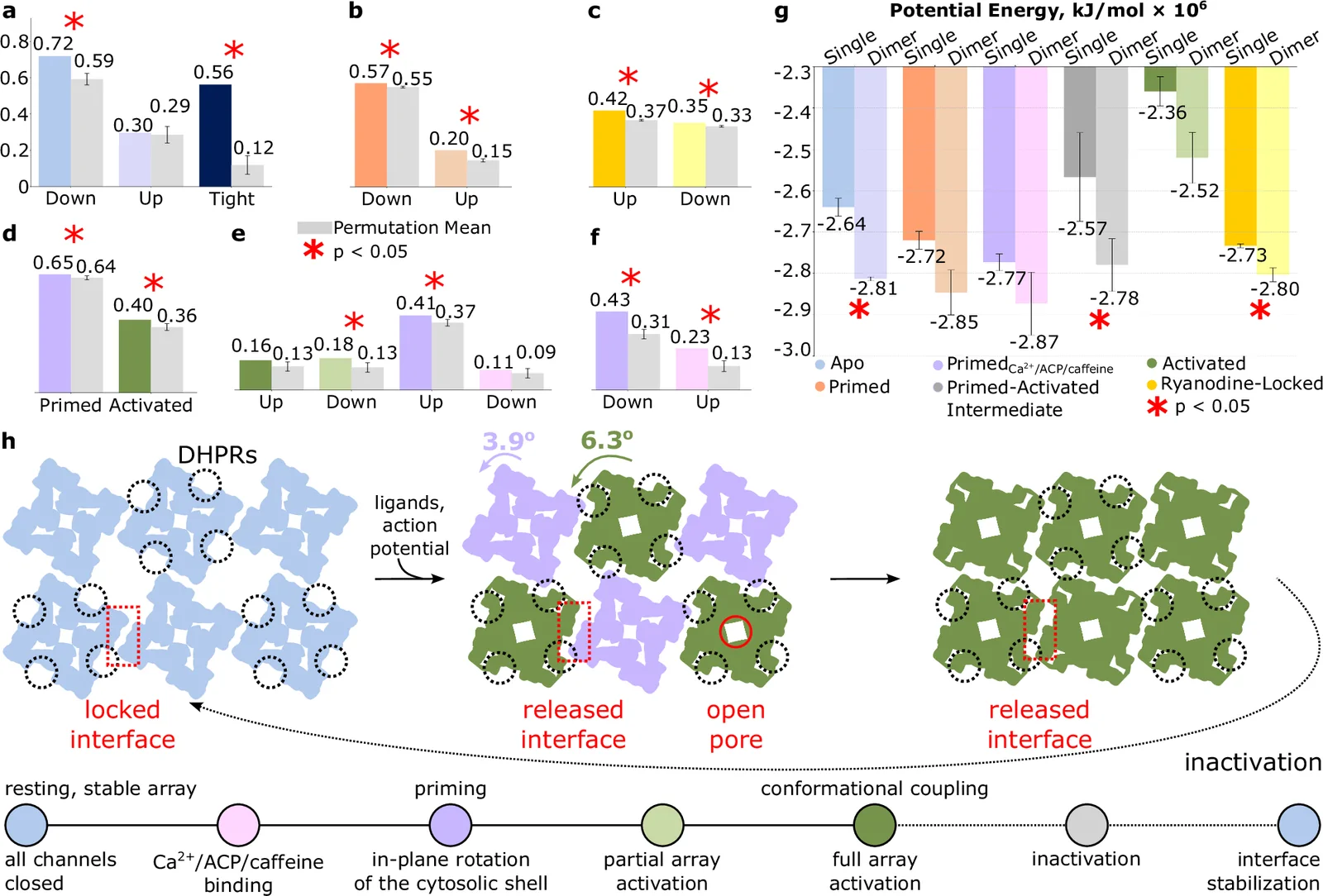
Statistical analysis of neighbour pairing provides direct evidence of functional coupling within the RyR1 lattice. **a–f** Statistical analysis of neighbor pairing. Bars show the mean fraction of adjacent channels sharing the same class. Grey bars represent the mean ± s.d. of the null distribution (10,000 permutations) obtained by shuffling class labels over the neighbor graph. Enrichment of same-class neighbors was assessed using a one-sided, non-parametric permutation test (null hypothesis: random class arrangement). *P*-values indicate the proportion of permutations meeting or exceeding the observed value. No adjustment for multiple comparisons was applied. Detailed statistical values for all comparisons in panels (**a**–**f**) are provided in Supplementary Table 2. Statistically significant enrichment indicates non-random, cooperative clustering for: **a** Same-class clustering for the EGTA-ET dataset. **b** Same-class clustering for the ActCa dataset. **c** Same-class clustering for the Ryanodine dataset. **d** Same-state clustering for the ActMix dataset, revealing a significant enrichment of Activated-Activated pairs (*p* = 0.018). **e** Same-class clustering for the ActMix dataset. **f** Same-class clustering for the ActMix-ET dataset. **g** Potential energy values calculated for each functional state for a single channel versus the dimer from energy minimization. The bars for the single channels represent the sums of two channels for a direct comparison with the corresponding dimers. Asterisks (*) denote statistically significant differences (*p* < 0.05). Detailed statistical values for all comparisons are provided in Supplementary Table 4. Schematic model of coupled RyR1 activation within the native lattice. Stable BSol-mediated contacts maintain neighboring receptors in the closed Apo state. Activating ligands induce priming and rotation of the cytosolic shell, followed by remodeling of the inter-receptor interface and pore opening. Conformational changes then propagate through neighboring RyR1–RyR1 contacts, promoting cooperative activation and coupled gating.

We obtained structural evidence of functional coupling by analyzing the conformational states of neighboring channels within the array. Statistical analysis of class assignments for cytosolic shell conformations within thousands of neighboring RyR1 pairs (Fig. 4a–f, Supplementary Table 2) demonstrated that adjacent channels showed a strong tendency to adopt similar conformations, resulting in statistically significant functional coupling for most of the classes. Most importantly, in the ActMix dataset, where both closed and open channels coexist, receptors in the open state were significantly more likely to be paired with other open-state receptors (Fig. 4c, *p* = 0.018). This correlation provides statistical evidence for the structural mechanism for coupled gating, confirming that conformational changes physically propagate via inter-channel contacts.

We performed a quantitative analysis of these contacts across the activation cycle (Supplementary Table 3) and estimated potential energies of each state for a single channel and the corresponding dimer by energy minimization (Fig. 4g, Supplementary Table 4). This simplified calculation, which does not account for the energetic contributions of lipids, ligands, or solvation, provides a theoretical approximation of the intrinsic strain within the protein component of the system. Although the potential energy values were consistently smaller for the RyR1 dimers compared to the sum of two single channels in each functional state, this stabilizing effect was statistically significant in the Apo state (Fig. 4g, Supplementary Table 4), quantifying the mechanical “locking” of the resting array.

Together, these data provide a mechanism for coupled gating. In the Apo state, neighboring channels form an extensive and stable interface of ~512 Å² with multiple interacting residues (Supplementary Table 3), consistent with a mechanically locked array. The statistically significant decrease in the potential energy of the dimer compared to a single channel (Fig. 4g) highlights the stabilizing effect of the array packaging. The initial priming step with Ca^2+^ does not weaken this contact and slightly expands the interface to ~602 Å^2^. Subsequent steps in the activation sequence, however, lead to a disruption, with the contact area reducing to 121–256 Å^2^. This progression from a large, stable interface to a smaller, less stable one represents a mechanistic insight: ligand binding induces a conformational rearrangement at the distinct receptor-receptor interface that remodels inter-channel contacts, thereby poising the local cluster for cooperative activation. Interestingly, the fully open Ryanodine-locked state does not lead to interface dissociation but instead forms a distinct, stable configuration with the largest observed contact area of ~984 Å^2^. This substantial tightening is possibly a consequence of the pronounced rotational shift during maximal pore dilation, forcing the BSol domains into a distinct, tighter-packed arrangement to accommodate the global structural changes. Consequently, lattice interactions have a considerable effect on the potential energy in this tightly packed state.

## Discussion

Our results provide high-resolution structural views of the RyR1-RyR1 interface along the receptor activation sequence, revealing the physical mechanism that underpins the long-observed physiological phenomenon of coupled gating. We propose a mechanism for RyR1 activation, shifting the focus from an individual channel event to a cooperative process orchestrated by a mechanically coupled receptor lattice. In this model (Fig. 4h), stable BSol-BSol interactions in the resting states lock the array, lowering open probability and preventing aberrant Ca^2+^ leak. The binding of activating ligands to single channels introduces conformational strain across this inter-receptor interface. The stored potential energy lowers the activation barrier for the cluster of channels, priming them to open synchronously. This lattice-based allostery provides a clear structural explanation for the constrained, rotation-dominant gating motion observed in situ.

Our results define the combined impact of native lipids, FKBP12, and RyR1-RyR1 lattice contacts as stabilizers of RyR1 in the closed state, preventing spontaneous activation and ensuring precise Ca^2+^ signaling control. The dual-gate pore architecture and wider pore dilation, coupled with a relatively low pO of 0.35 observed in native membranes, imply that endogenous lipid compositions significantly influence RyR1 sensitivity and function. Recent studies^18^ show that lipid composition affects purified RyR1 gating, supporting this conclusion. Moreover, RyR1 activation sensitivity highly depends on the detergent used during the purification^17^. The importance of this native lipid context is further underscored by the absence of the S0 helix^27^, a feature implicated in enhancing Ca^2+^ sensitivity in some artificial lipid mimetic systems but not observed here. Therefore, the endogenous lipid environment within native membranes emerges as a critical regulator of physiological gating behavior. Furthermore, while we have not detected ordered calsequestrin, triadin, and junctin in our structures, they may further reduce the open probability of RyR1 in native membranes.

Our ability to obtain high-resolution structures from cryo-ET reflected an end-to-end improvement of throughput: we increased the number of RyR1 particles per tomogram by optimizing sample enrichment and grid preparation, collected more tomograms per session using automated acquisition with PACE‑tomo^30^, and accelerated downstream analysis through streamlined data processing implemented in tomoBEAR^31^, sensitive template matching in PyTOM-TM^32,33^, and subtomogram averaging capabilities in RELION4^34^ and RELION5^35^. These improvements raised our effective yield to ~70 particles per tomogram (about ~13,000 particles per dataset), exceeding earlier reports that recovered on the order of 30–60 particles per tomogram^21,24^. In parallel, we used single-particle analysis when vesicles could be imaged in sufficiently thin ice; as expected, SPA generally produced higher-resolution reconstructions due to the larger particle numbers. The two approaches were complementary: cryo-ET clearly showed the RyR1 lattice, while SPA increased particle statistics, and together they enabled structural characterization, including determination of RyR1 dimer structures.

The clinical significance of the interchannel contacts is enforced by several disease-causing mutations at this interface (Supplementary Fig. 31). Residues HIS2976^36^ and SER3217^37^ linked to MHS1, as well as GLU3238^38^ linked to CMYO1A, are located at the interface. Mutation hotspots occur in the post-RY3&4 part of the BSol, a key structural element of the interface: GLU3290^37^, PRO3410, and ASP3501^36^ linked to MHS1, LEU2963^39^, and ARG3366^40^, ASN3326, CYS3402^41^, PRO3527^42^, and ARG3539^43^ linked to CMYO1A&B (Supplementary Fig. 31). We propose that single amino acid substitutions at these hotspots destabilize the inter-tetramer contacts, disrupting the coordinated, cooperative gating of the channel array. This allosteric dysregulation provides a molecular basis for the abnormal Ca^2+^ leak and channel hypersensitivity that drive the pathophysiology of these severe muscle disorders. Our structural elucidation of the native RyR1 lattice therefore provides a framework for understanding these channelopathies and identifies the interchannel interface as a potential therapeutic target.

Our analysis provided the most native high-resolution structures of RyR1 to date and revealed the details of the mechanism of ligand-modulated activation. While our preparations contained few putative T-tubules, similar to our previous report^21^, they were rare, and the averages did not have T-tubule membranes and DHPR; therefore, our analysis does not explain the voltage sensitivity of the EC-coupling machinery. The loss of the T-tubules has also induced a higher SR membrane curvature, varying between datasets (Supplementary Fig. 32). The local membrane curvature in the channel vicinity appeared to be preserved and resembled the structure of RyR1 in skeletal muscle^22^ (Supplementary Fig. 30). Interestingly, calmodulin, an important regulator of RyR1, was absent in all our conditions, while some auxiliary regulators, calsequestrin and junctin, were not resolved, likely due to their dynamic binding behavior. However, in the ActCa conditions, up to 89% of particles had neighbours, suggesting preservation of the native lattice contacts. Future structural investigations incorporating these additional regulators and cellular structures will further clarify RyR1 behavior in situ.

In conclusion, our study reshapes the understanding of RyR1 activation, moving beyond a single-receptor description to a model of a cooperative, mechanically interconnected supramolecular machine. We have identified the BSol-mediated dimer interface as the structural basis for coupled gating, demonstrated how lattice constraints dictate a rotation-driven activation mechanism, and provided a molecular framework for a major class of human muscle diseases. These results emphasize the physiological importance of RyR1 arrays as integrated functional units and provide perspectives on EC-coupling and the development of targeted therapeutics for RyR1-linked channelopathies.

## Methods

### Ethics

The research complies with all relevant ethical regulations. Hind leg meat from male and female rabbits, aged 75–77 days, was purchased from Bauer Kaninchen Spezialitäten GmbH (Lohe 2, 74632 Neuenstein).

### Sample preparation

SR vesicles were isolated based on the previously described protocol^44^. Sixty grams of fresh rabbit skeletal muscle tissue from the hind leg and back was ground using a meat grinder and then homogenized using a precooled blender with the addition of 300 ml of homogenization buffer (0.5 mM EDTA, 10% (300 mM) sucrose, 20 mM sodium pyrophosphate (Na_4_O_7_P_2_), 20 mM NaH_2_PO_4_ and 1 mM MgCl_2_, pH 7.1) plus the following protease inhibitors cocktail: 2.6 μg/ml aprotinin, 1.4 μg/ml pepstatin and 10 μg/ml leupeptin. Homogenization in the blender was performed at its top speed for 2 to 3 min. Homogenates derived from a total of 180 g of muscle were centrifuged in a Beckman Coulter rotor JA 8.1 fixed-angle rotor at 6800 × *g* at 4 °C for 20 min. The resulting supernatant was filtered through 3 layers of medical bandage to remove cell debris and fat tissue, and then ultracentrifuged in a Beckman Coulter Type 45Ti fixed-angle rotor at a speed of 50,000 × *g* at 4 °C for 1 h. The membrane pellets were directly dissolved in an ice-cold homogenization buffer and divided into 3 mL aliquots. One aliquot was used immediately in the next step, and the remaining aliquots were snap-frozen in liquid nitrogen and stored at −70 °C for future use. The frozen membrane pellet was thawed on ice and subjected to a discontinuous sucrose gradient with steps of 0.5 ml 50%, 2 ml 45%, 2 ml 42%, 2 ml 40%, 2 ml 38 %, 1 ml 36%, 1 ml 34%, 1 ml 32%, 1 ml 28%, 1 ml 25% and 0.5 ml 14% sucrose. We modified the discontinuous sucrose gradient layover pattern from the previous protocol to enrich the heavy SR fraction in the target bands, improving the amount of RyR1 particles per field of view in our data. The gradient was always constructed immediately before use, while the solutions were prepared one day in advance and stored at 4 °C. The sucrose gradient was then centrifuged in a Beckman Coulter SW 32.1Ti swinging-bucket rotor at 138,000 × *g* at 4 °C for 90 min. Bands at the interface of the 38 and 40% sucrose phases and at the interface of the 40 and 42% sucrose phases were extracted from the sucrose gradient, diluted with a dilution buffer (0.1 mM EGTA, 20 mM Na_4_O_7_P_2_, 20 mM NaH_2_PO_4_, and 1 mM MgCl_2_, pH 7.1) to 12 ml and then ultra-centrifuged in a Beckman Coulter 70.1Ti fixed-angle rotor at a speed of 40,000 × *g* at 4 °C for 40 min. The final membrane pellet was resuspended in 50 µl of dilution buffer. The final sample was diluted to a total protein concentration of ~30 mg/ml, estimated with a NanoDrop™. EGTA was added to the Apo-state sample aliquots to a final concentration of 5 mM. CaCl_2_ stock solution was added to the ActCa and ActMix sample aliquots to reach the target concentration of 30 µM free Ca^2+^, according to MaxChelator^45^, with free Mg^2+^ and added ACP (if applicable) taken into account. The free Mg^2+^ concentration was approximately 10–15 µM, as it was mostly sequestered by the pyrophosphate, and even lower at approximately 0.4 µM in the 2 mM ACP-containing samples. ACP and caffeine were added to the mixture to the final concentrations of 2 mM and 5 mM, respectively, and incubated at 4 °C for 30 min. After the addition of ryanodine to a final concentration of 5 µM, the sample was incubated overnight at 4 °C to ensure complete binding.

### Cryo-EM and Cryo-ET Data Collection

Grids were glow-discharged and flash-frozen for cryo-EM using a Vitrobot™ Mark IV (Thermo Fisher) at 4 °C and 95% humidity. 4 μl of sample was applied to 300-mesh copper Quantifoil® R 2/1 grids with holey carbon film. For tomography, the gold R 2/2 grids with gold support were used, and the sample was mixed with 10-nm colloidal gold fiducials (10:1 ratio). The grids were blotted with Whatman® No. 1 filter paper, 2 per side, with blot time 3.5 s, wait time 5 s, and blot force 10, and plunged into liquid ethane cooled by liquid nitrogen. The blotting papers were washed in 100 µM EGTA and then in Milli-Q water to get rid of the contaminating Ca^2+^^46^. We optimized the sample concentration and volume applied to the grid, as well as the blot time to ensure the proper ice gradient along the hole for optimal data collection.

Imaging of ActMix-ET and all SPA datasets was performed on a Thermo Fisher Titan Krios G3 microscope operated at 300 kV, equipped with a Gatan K3® direct electron detector and a Gatan BioQuantum K3® energy filter. SPA data were collected with EPU (Thermo Fischer Scientific) at a magnification of 64,000×, resulting in a nominal pixel size of 1.38 Å/pixel, calibrated to the value of 1.366 Å/pixel. Single-axis tilt series (−48° to +48°) were collected using a dose-symmetric tilt scheme^47^ with 3° intervals implemented in SerialEM^48^ and PACE-tomo^30^ at a magnification of 53,000×, resulting in a nominal pixel size of 1.68 Å/pixel. Acquisition of the EGTA-ET dataset was performed at the TFS in Eindhoven on the Titan Krios G4 version equipped with the Falcon 4i detector and Selectris X energy filter with the Thermo Fisher Scientific Tomography 5 Software at a magnification of 64,000×, resulting in a nominal pixel size of 1.894 Å/pixel, calibrated to the value of 1.948 Å/pixel. For all tomograms, the electron dose for the images was set to ~4 e^−^/Å^2^, recorded in 10 frames; the total exposure was about 128 e^−^/Å^2^. Nominal defocus was set between 2.5 and 5 μm.

### Cryo-ET data processing

All cryo-ET data were pre-processed under the TomoBEAR^31^ workflow as previously described. In brief, super-resolution non-gain-corrected tilt movies were sorted into separate folders by tomogram index and by tilt angle within these folders. Motion correction was done with MotionCor2^49^ without patches for local motion tracking, the last frame as a reference frame, and Fourier cropping of the output averages by a factor of 2. EGTA-ET data EER^50^ movies were motion-corrected with RELION^51^ with a physical Nyquist (4k x 4k) as well as super-resolution (8k x 8k) rendering. Seventy-two EER frames were grouped, which resulted in a 0.5 e^−^/frame dose fractionation. Raw stacks were generated from the motion-corrected images with the newstack program from IMOD^52^. Dynamo tilt-series alignment routines^53^ were used to generate the fiducial alignment models that were imported to Etomo^54^ from IMOD, and the alignment models were refined, followed by the generation of the aligned stacks. GCTF^55^ was used to estimate defocus values per tilt, and these values were input to ctfphaseflip^56^ from IMOD, followed by gold bead detection and erasing. CTF-corrected gold-erased aligned stacks were binned by a factor of 8, and bin8 tomograms were reconstructed.

For the EGTA-ET dataset, template matching on 194 bin8 tomograms (nominal pixel size 15.152 Å/pixel, box size 40 pixels) was done with Dynamo^57,58^ called via TomoBEAR^31^. 44,333 particles were extracted based on the threshold, defined per tomogram as 7 standard deviations above the mean for each respective cross-correlation volume, with a cap set to a maximum of 700 particles per tomogram. An alignment round of subtomograms in bin8 was done in SUSAN (https://github.com/rsanchezloayza/SUSAN), with 2 iterations of only translational search and without symmetry, followed by two more iterations with a local angular search within 10° with a 1° step and C4 symmetry. All particles were imported into RELION4^34^ together with the tilt stacks, the corresponding tilt series alignments, fitted CTF (.defocus files), as well as the acquisition order and dose distribution information (Supplementary Fig. 33). Four successive rounds of 3D classification in bin4 with global angular search resulted in a final subset of 13,726 particles. Particles were subjected to 3D refinement in bin4 and then in bin1. The tomograms and particles were then re-imported into RELION4^34^ at a super-resolution pixel size (8k x 8k images, calibrated pixel size 0.974 Å/pixel) and subjected to 3D refinement in bin1.5 (calibrated pixel size 1.461 Å/pixel, box size 340 pixels), resulting in a 7 Å map. Polishing, CTF refinement, and another 3D refinement produced the consensus 4.6 Å map, sharpened with a B-factor of −101 Å^2^. Focused refinement was done on the activation core and TMD parts (CSol, TaF, TMD, CTD) in bin1 (calibrated pixel size 0.974 Å/pixel, box size 340 pixels). Two rounds of polishing, CTF refinement, and Refine3D, and a final Refine3D on the signal-subtracted particles (the remaining parts of the receptor were subtracted from the particles based on the focused mask) resulted in the 4 Å map. Another focused refinement was done on the cytosolic shell part (NTD-A, NTD-B, NSol, SPRY1, SPRY2, SPRY3, RY1&2 plus FKBP12) with C4-symmetry expanded particles in bin1. Refine3D on the signal-subtracted particles converged at 4.2 Å, and one extra defocus refinement brought the resolution down to 4 Å. The other part of the cytosolic shell (BSol and RY3&4 domains) couldn’t be improved by focused refinement. Both focused maps were sharpened with a B-factor of −80 Å^2^ and filtered according to the local resolution.

Multiple 3D classifications without alignment were run on bin3 particles, with a whole-channel mask and varying parameter sets: 4, 6, 8, 10, and 12 for the number of classes; 4, 8, 16, 32, 64, and 128 for the regularization T (tau2_fudge). In addition, the particles were symmetry-expanded to C4, masked with a whole-chain mask, signal-subtracted, 3D refined, and subjected to the same 3D classification rounds. Classes were independently compared within tetramer and monomer classification runs, and three stable conformations were identified based on the cytosolic shell conformation: either lower “Down” or higher “Up”, with respect to the SR membrane plane, when compared to the consensus reconstruction. The third class had the shell domains positioned closer to the channel axis and was therefore termed “Tight”. The particles that belonged to the same class across all runs were taken, based on the unique particle IDs intersection, and subjected to Refine3D in bin3. 4.402 particles in the “Down” conformation resulted in the 6 Å map, B-factor −200 Å^2^; 3.555 particles in the “Up” conformation resulted in the 6 Å map, B-factor −200 Å^2^; 1.088 particles in the “Tight” conformation resulted in the 7 Å map, B-factor −200 Å^2^; Pixel size was calibrated with an atomic model. The global resolution was estimated from FSC with a 0.143 threshold cut-off.

To obtain the RyR1-RyR1 dimer reconstruction, clustering of particles was performed by the script adapted from the ref. ^59^. A histogram of neighboring particles’ locations in the reference frame, analogous to the neighbor 3D heat map, showed four distinct C4 symmetry-related peaks. The subset of 219 particles was selected with neighbors located only within the peak regions by applying a mask on the histogram. This particle subset was then symmetry-expanded to C4 (876 particles), reconstructed with a big enough box size to accommodate two channels (bin4 pixel size 3.896 Å/pixel, box size 340), re-centered, cropped to a 200 pixel box, and subjected to 3D classification without alignment with a focused mask on the neighbor density. The two best classes with 222 particles showing a RyR1 density within the neighbor mask were selected and refined with a full dimer mask, giving a RyR1 dimer map at 26 Å resolution. No symmetry was applied in order not to bias the interface density.

For the ActMix-ET dataset, the template matching on 182 bin8 tomograms (pixel size 13.44 Å/pixel; all binning levels are stated relative to the physical pixel size of 1.68 Å/pixel) was done with PyTOM^32,33^, with a 7^o^ angular sampling and a normalization with the whitening filter. The EMD-10637^21^ was used as the initial template, rescaled to a pixel size of 13.44 Å and a box size of 32 pixels, and modulated with per-tilt, CTF, and dose weighting. 3000 top hits per tomogram were extracted, and 6 tomograms were omitted after visual inspection of the cross-correlation volumes because there were no sharp peaks. All 524,185 extracted particles were combined into a single STAR file^60^, with the XYZ coordinates upscaled by a factor of 8 to match the unbinned data, and imported into RELION5^35^ together with the tilt stacks, the corresponding tilt series alignments, exposure distribution, fitted CTF, and acquisition order files. The new .mdoc files with all the necessary metadata were created and linked into a RELION5^35^ project directory together with the raw frames. Motion-corrected micrographs and CTF values from TomoBEAR^31^ were also linked, and the corresponding STAR files^60^ were created. Tilt series alignment folders from TomoBEAR^31^ were linked into the RELION5^35^ AlignTiltSeries job output, and the tomograms STAR^60^ file was created. All particles were extracted in bin8 with a pixel size of 13.44 Å/pixel and a box size of 32 pixels as 2D stacks in float16, and subjected to multiple rounds of 3D classification with 25 iterations, local angular search within 10°, 8 classes, no applied symmetry, and an initial low-pass filter of 40 Å. Each classification was monitored for convergence by plotting the number of particles per class per iteration. Classes that had the same number of particles in the last 10 iterations of classification were denoted as stable. Particles belonging to the stable classes that didn’t show RyR1 features were excluded from the downstream processing. Particles that belonged to stable classes with good-looking class averages were kept and subjected to the next classification round. Particles that were jumping between “good” and “bad” classes were subjected to the next classification round, separate from the stable particles. At the end, all good and stable classes were pooled together and classified further. The whole process was repeated until there were no more bad classes or an occasional bad class containing only a small fraction of all particles. (Supplementary Fig. 34). From this point on, the C4 point group symmetry was applied throughout. The final subset of 17,229 particles was refined in bin8 and in bin2 (pixel size 3.36 Å, box size 128 pixels), giving a 6.8 Å resolution map and 13,358 particles left after removal of the duplicates. Refine3D in bin1 (pixel size 1.68 Å, box size 256 pixels), followed by Polishing and CTF refinement, resulted in the 5.3 Å map. Two successive rounds of Refine3D with progressively smaller angular steps of 0.5°, and 0.2 to 0.1° (with 2x oversampling) gave a 4.94 Å map. The final round of Polishing, CTF refinement, and Refine3D with 0.2 to 0.1° steps resulted in the 4.89 Å consensus map, which was sharpened with the B-factor of −111 Å^2^, automatically fitted to the Guinier plot. The last two rounds of 3D refinement were run with Blush regularization^61^, which has slightly improved the resolution compared to the standard reconstruction. The second digit after the decimal point in the resolution estimate is given in the latter case to highlight the marginal improvement of the resolution by a couple of Fourier pixels.

A mask was created to encompass the TMD, CTD, and TaF domains; the particles were recentered on the center of mass of this mask and subjected to focused refinement, resulting in a 4.4 Å resolution map, sharpened with a B-factor of −61 Å^2^. The consensus particle set was symmetry-expanded to C4, and the corresponding particle trajectory STAR^60^ file from the Polishing job was adjusted to take this into account. Three masks were created, encompassing two huge portions of the cytosolic shell (NTD-A, NTD-B, NSol, SPRY1, SPRY2, SPRY3, RY1&2 plus FKBP12 and BSol with RY3&4 respectively) and the third one for the JSol and CSol domains. Particles were recentered on each of these masks’ centers of mass, with the box cropped to 200 pixels, and subjected to focused refinement. Another round of CTF refinement was performed for the first cytosolic shell part in order to account for the defocus differences, but it did not boost the resolution any further. The final focused maps had the resolutions of 4.4, 5.5, and 4.5 Å and were sharpened with B-factors of −101, −183, and −80 Å^2,^ respectively.

The consensus particle set was subjected to two parallel 3D classifications without alignment in bin2 with the whole-channel mask, 25 iterations, applied C4 symmetry, an initial low-pass filter of 15 Å, regularization T (tau2_fudge) = 4 (default), and either 4 or 8 classes. In each run, two converged classes were identified, with the cytosolic shell domains being either lower (“Down”, 4760 and 3808 particles for the 4- and 8-class runs, respectively) or higher (“Up”, 3012 and 2635 particles for the 4- and 8-class runs, respectively) with respect to the SR membrane plane, when compared to the consensus reconstruction. The particles that overlapped between each class of the two classification jobs were taken, resulting in 3346 particles (64% overlap) in the “Down” conformation and 1541 particles (37.5% overlap) in the “Up” conformation. Both subsets were refined in bin1 with fine angular sampling and Blush regularization^61^, resulting in the 4.9 Å resolution map sharpened with the B-factor of −110 Å^2^ for the “Down” state, and the 6.8 Å resolution map sharpened with the B-factor of −115 Å^2^ for the “Up” state. Pixel size was calibrated with an atomic model. The global resolution was estimated from FSC with a 0.143 threshold.

To obtain the RyR1-RyR1 dimer reconstruction, the clustering of particles was analysed by the script adapted from the ref. ^59^. A histogram of neighbouring particles’ locations in the reference frame, analogous to the neighbour 3D heat map, showed four distinct C4 symmetry-related peaks. The subset of 704 particles was selected with neighbours located only within the peak regions by applying a mask on the histogram. This particle subset was then symmetry-expanded to C4 (2816 particles), reconstructed with a big enough box size to accommodate two channels (bin4 pixel size 6.72 Å/pixel, box size 256), re-centered, and subjected to two rounds of 3D Refinement with a focused mask on the neighbour density, followed by 3D classification without alignment into 8 classes. The best class with 1035 particles showing a RyR1 density within the neighbor mask was selected and refined with a full dimer mask in bin4 and then bin2. The map was reoriented to align the C2 symmetry axis with the Z axis, but the C2 symmetry was not applied in order not to bias the interface density.

### SPA data processing

Most of the image processing steps were performed in cryoSPARC^62^ unless stated otherwise. Movie frames were aligned with the Patch Motion job. After CTF estimation with Patch CTF, low-quality micrographs with high ice thickness, large motion, and low CTF fit resolution were removed from each dataset. RyR1 particles were picked with a template-based particle picker. For the first dataset that was processed, the particles were first picked with blob picking, and the templates were selected from the 2D classes. Low-resolution ab initio models were generated from the previously obtained cryo-ET map of RyR1 in the SR membrane. Particles were first extracted in bin4 (pixel size 5.52 Å/pixel) with a large box size of 128 pixels (would correspond to 512 pixels in bin1) to account for the membrane signal. Iterative rounds of 2D classification and heterogeneous refinement further removed particles that do not contribute to high-resolution reconstructions. 2D classifications were run with 200 classes, 40 online-EM iterations, and a batch size of 400. Heterogeneous refinements were run with 4 references, of which only one was a “good” one, and the other three were “decoys” with either an empty membrane or blob density. After cleaning up the particle sets for each dataset, global consensus reconstructions of RyR1 were carried out with Non-Uniform refinement^63^ and C4 symmetry enforced in bin1 (pixel size 1.38 Å/pixel, box size 440 pixels), with minimization over per-particle scale, defocus, and global CTF refinements. In global homogeneous/NU refinement^63^ maps, the cytoplasmic domain of RyR1, especially BSol, exhibits a significant degree of motion suggested by smeared or unresolved local map quality. To improve the local resolution, four local soft masks were generated using UCSF ChimeraX^64^. The particle stacks were C4 symmetry-expanded and were subjected to focused refinements with local masks (Supplementary Figs. 3–8). The local maps from focused refinements of C4-expanded particles with different local masks around FKBP12/NTD-AB/NSol/SPRY1-3/RY1&2, BSol/RY3&4, JSol/CSol, and TaF/CTD/TMD domain regions were aligned to the global consensus maps. The local maps were then combined in UCSF ChimeraX^64^ by taking the maximum value at each map voxel. Classifications without alignment were performed either in cryoSPARC^62^ or RELION4^51,65^. In CryoSPARC^62^, classifications were run with 10 classes, C4 symmetry, 6 Å filter resolution, 10 Å initial low-pass filter, input per-particle scale, and hard classification on. Particles were imported into RELION4^51,65^ with pyEM^66^, and 3D classification without alignment in bin2 was done for the ActMix-SPA dataset, with two parallel runs with either 4 or 8 classes. “Up” and “Down” classes were identified both for the closed and the open pore states and subjected to Refine3D in bin1 with local angular searches. Pixel size was calibrated with an atomic model^14^. The global resolution was estimated with a 0.143 threshold, and the local resolutions were estimated from FSC with a 0.5 threshold. See Supplementary Figs. 35, 36 for the processing numbers of micrographs, particles, and other details.

To obtain the dimer structures from the SPA data, all particles were symmetry-expanded, cropped to a bigger box, and subjected to multiple rounds of 3D classification with the neighbor-only mask and the dimer map obtained from tomography data as the initial reference. After the clean dimer particle subset was obtained, the same refinement strategy was utilized to get the final reconstruction. Models obtained from the monomer maps were then rigid-body docked into the dimer maps, and the interface sidechain conformations were optimized with a short simulation run in ISOLDE^67^. The relaxed interface models were analysed with the PISA^68^ web server.

### Model building

First, the pixel size for each map was calibrated iteratively with the *fitmap* command in ChimeraX^64^ until the global minimum was found by monitoring the convergence for different domains. All models were built into the composite maps starting from 7TZC^69^ as the initial model. For each map, the model of one protomer was split by domains, and each domain was independently rigid-body fit into the density. The peptide bonds between domains were manually added with ISOLDE^67^. The protomer model was copied three times, rigid-body fit into symmetry-related density, and combined into a single model. The full model was flexibly fit in ISOLDE^67^ with intra-subunit position and angular restraints. The fitted model was then real-space-refined with PHENIX^70^. Pore profiles were calculated with HOLE2^71^.

### Analysis of structures

All model analysis and measurements were done in ChimeraX^64^. All publicly available models selected for the analysis were fetched from the PDB (https://www.rcsb.org/) and prepared in the following way. Each model was reindexed to the same chain IDs (RyR1 protomers A, B, C, D and E, F, G, H for the corresponding FKBP12s in the counterclockwise direction when viewed from the cytosolic “top” side) and then aligned to the reference model on the most rigid part of the TMD (residues 4820–4920).

To assess relative conformational changes between domains, pairwise displacements were calculated across all structural models using UCSF ChimeraX^64^. All alignments were performed on C-alpha atoms with the *align* command. For each pair, structures were first aligned on a chosen reference domain, after which the RMSD of the target domain was measured. A second alignment of the target domain itself was then performed to obtain the baseline RMSD for that domain, and this value was subtracted from the RMSD measured after reference-domain alignment to yield a normalized displacement. This analysis was carried out for the CSol relative to the TMD and for EF1&2 relative to the CSol and S2S3′ bundle, generating a pairwise distance matrix for all models. The distance matrix was subsequently analyzed by non-metric multidimensional scaling in MATLAB with *mdscale*, and the two-dimensional embedding was visualized in Python using *matplotlib*. The code^72^ is available at https://github.com/KudryashevLab/RMSDistance.

To calculate the cytosolic shell tilt and in-plane rotation angles, the center of mass was defined for each model with the *define centroid* command. The Z and X axes were then defined from the center with *define axis*. The axis for the cytosolic shell was defined from the residue number 348 Cα atom to the residue number 984 Cα atom of chain A, to match the definition in the previous reports^15,29^. The residue number 984 was chosen instead of the original number 1052 because of some modelling discrepancies between some earlier and later models in this less resolved region. Cytosolic shell tilt was calculated as the angle of this axis relative to the Z-axis, followed by subtracting the calculated value from 90 degrees. The subtraction from 90 degrees effectively gives the angle relative to the horizontal membrane plane. The sign of the resulting angle was determined from the Flexion-axis Z-vector sign (positive if the end-point is higher than the start-point along Z, negative if the end-point is lower than the start-point along Z). Because all models were positioned upside-down, the sign was inverted to match the flexion angle convention. The resulting values for the cytosolic shell tilt angle closely match the previously reported values^15,29^ (see Supplementary Table 5 for comparison). In-plane rotation was calculated as the angle of the shell axis relative to the X-axis, followed by subtracting the calculated value from 90 degrees. The sign of the resulting angle was determined from the Flexion-axis X-vector sign (positive if the end-point is higher than the start-point along X, negative if the end-point is lower than the start-point along X). As the X-axis is arbitrary, depending on the in-plane position of a model, it can also be defined as the axis between residue number 71  Cδ atom of chain B and residue number 71 Cδ atom of chain D. Values for all the models were assembled into the STAR^60^ file and plotted with *matplotlib*.

### Neighbour clustering analysis

Individual particles were assigned to the neighbouring pairs with the script adapted from the ref. ^59^. Only the pairs of particles engaged in the RyR1-RyR1 interaction were retained based on the unique particle IDs present in both STAR^60^ files for the monomer and dimer particle stacks, as well as the corresponding XY (and Z in case of tomograms) coordinates and poses. The obtained list of pairs was subjected to duplicate removal and symmetrization. To test whether channels belonging to the same class co-occurred in RyR1 channel pairs more often than expected by chance, a permutation-based randomization test was performed separately for each class. For each particle, all neighboring channels were identified from the particle list, and the fraction of neighbors sharing the same class label was computed directly. For a given class, the test statistic was defined as the mean same-class neighbor fraction across all particles assigned to that class with at least one neighbor. Under the null hypothesis that class labels are randomly arranged over the observed particle-neighbor graph (no preferential pairing by class), class labels were randomly permuted across all particles (preserving the number of particles per class and the observed neighbor network), and the test statistic was recomputed for each of 10,000 permutations, yielding an empirical null distribution. The one-sided *P* value for each class was calculated as the proportion of permutations in which the permuted statistic was greater than or equal to the observed statistic (testing for enrichment of same-class neighbors); thus, *P* values reflect the probability of obtaining an equal or higher level of same-class clustering under random assignment of classes. For visualization, histograms of the null distributions were plotted with the observed statistic indicated as a vertical line, together with the null mean ± s.d. as a point estimate with error bars.

### Potential energy calculation

For each RyR1 functional state atomic model, including both dimeric and monomeric forms, all non-protein molecules, such as small molecules and lipids, were removed. Small, unresolved loop regions within the structures were completed using the MODELLER^73^ software package. The quality of the generated models was evaluated using the DOPE score, and the top-scoring model for each state was selected^74–76^.

A system was then established for each obtained RyR1 model, containing only the protein, and parameterized using the CHARMM27 force field^77,78^. Energy minimization was subsequently performed using the Gromacs 2024.3 software^79,80^. The system utilized the Particle-mesh Ewald scheme (PME)^81^ method for long-range electrostatics with a 1.5 Å grid spacing and a 12 Å cutoff. Van der Waals interactions were also truncated at 12 Å, managed with a force-switch function applied between 10 Å and 12 Å. All bonds involving hydrogen atoms were constrained. The minimization was carried out using the steepest descent algorithm with a step size of 0.1 Å. The process was iterated until the maximum force (FMAX) on any atom converged to a value below 1000 kJ mol^−^^1^ nm⁻^1^. Finally, the potential energy (PE) of the resulting minimized structure was calculated. For each of the models, the energy minimization was independently performed three times (*n* = 3).

Differences in potential energy between single and dimer channel states within each functional state were first assessed using two-tailed independent two-sample t-tests due to unmatched replicate measurements. This found statistical significance of energy changes upon dimerization for the Apo, intermediate Primed-Activated, and Ryanodine-locked dimer states. To test if potential energies differed significantly across the states, one-way, two-tailed Analysis of Variance (ANOVA) tests were conducted separately for the single channels (F(4,10): 26.27, p-value: 2.76e-05) and dimers (F(5,12): 11.67, *p*-value: 0.0003). In both cases, the Activated state was found significantly different from the other with Tukey’s Honest Significant Difference (HSD) post-hoc tests (adjusted p-values: 0.0009 (Apo), 0.0001 (ActCa), 0.0 (ActMix-Primed), 0.0001 (Ryanodine-locked) for the single channel and 0.0014 (Apo), 0.0005 (ActCa), 0.0003 (ActMix-Primed), 0.0038 (ActMix-Primed-Activated), 0.0019 (Ryanodine-Locked) for the dimer). A one-way ANOVA was also applied to the difference values (dimer minus the sum of two single channels), showing no significant change in the magnitude of dimerization energy gains across states (F(5,12): 1.36, *p*-value: 0.3). All statistical analyses were conducted in Python using the SciPy and Statsmodels libraries. A significance threshold of *p* < 0.05 was applied throughout.

### Statistics & reproducibility

No statistical method was used to predetermine sample size. No data were excluded from the analyses. The experiments were not randomized. The Investigators were not blinded to allocation during experiments and outcome assessment. Statistical analyses of neighbor pairing (functional coupling) within the RyR1 lattice were performed using a one-sided, non-parametric permutation test with 10,000 permutations to assess the enrichment of same-class neighbors, with a null hypothesis that class labels are randomly arranged over the observed graph. P-values were calculated as the proportion of permutations in which the permuted test statistic was greater than or equal to the observed value.

For thermodynamic and potential energy calculations, energy minimization was performed independently three times (*n* = 3) for each model using the steepest descent algorithm in GROMACS 2024.3^79,80^ with the CHARMM27 force field^77,78^. Potential energy differences between single channels and dimers were assessed using two-tailed, independent two-sample *t*-tests. Variations across different functional states were evaluated using one-way, two-tailed ANOVA tests, followed by Tukey’s Honest Significant Difference post-hoc tests for pairwise comparisons. A significance threshold of *p* < 0.05 was applied throughout, and no adjustment for multiple comparisons was made.

To ensure reproducibility, structural reconstructions and classifications were validated across multiple independent datasets, including cryo-ET and single-particle cryo-EM, and multiple grid preparation trials. The details are described in the methods section and in Supplementary Table 1. Subtomogram averaging and single-particle analysis protocols utilized reported available complementary image processing workflows to ensure the consistency of structural states.

### Reporting summary

Further information on research design is available in the Nature Portfolio Reporting Summary linked to this article.

## Supplementary information

Supplementary information

Reporting Summary

Transparent Peer Review file

## Source data

Source Data for Figures

## Data Availability

Previously reported atomic coordinates of RyR1 used for analysis in this study are available in the Protein Data Bank (PDB) under accession codes: 5TB0 [https://doi.org/10.2210/pdb5tb0/pdb] (Apo-state RyR1, EGTA, consensus); 5TB1 [https://doi.org/10.2210/pdb5tb1/pdb] (Apo-state RyR1, EGTA, class 1); 5TB2 [https://doi.org/10.2210/pdb5tb2/pdb] (Apo-state RyR1, EGTA, class 2); 5TB3 [https://doi.org/10.2210/pdb5tb3/pdb] (Apo-state RyR1, EGTA, class 3); 5TB4 [https://doi.org/10.2210/pdb5tb4/pdb] (Apo-state RyR1. EGTA, class 4); 7K0T [https://doi.org/10.2210/pdb7k0t/pdb] (Apo-state RyR1, ACP); 8SEN [https://doi.org/10.2210/pdb8sen/pdb] (Apo-state RyR1, EGTA); 7TDG [https://doi.org/10.2210/pdb7TDG/pdb] (Inactivated-state RyR1, consensus); 7TDJ [https://doi.org/10.2210/pdb7TDJ/pdb] (Inactivated-state RyR1, class 1); 7TDI [https://doi.org/10.2210/pdb7TDI/pdb] (Inactivated-state RyR1, class 2); 7TDK [https://doi.org/10.2210/pdb7TDK/pdb] (Inactivated-state RyR1, class 3); 5T15 [https://doi.org/10.2210/pdb5T15/pdb] (Primed-state RyR1, 30 uM Ca2 + , consensus); 5T9M [https://doi.org/10.2210/pdb5T9M/pdb] (Primed-state RyR1, 30 uM Ca2 + , class 1); 5T9N [https://doi.org/10.2210/pdb5T9N/pdb] (Primed-state RyR1, 30 uM Ca2 + , class 2); 5T9R [https://doi.org/10.2210/pdb5T9R/pdb] (Primed-state RyR1, 30 uM Ca2 + , class 3); 5T9S [https://doi.org/10.2210/pdb5T9S/pdb] (Primed-state RyR1, 30 uM Ca2 + , class 4); 5TAP [https://doi.org/10.2210/pdb5TAP/pdb] (Primed-state RyR1, CaffATP/EGTA, consensus); 5TAS [https://doi.org/10.2210/pdb5TAS/pdb] (Primed-state RyR1, CaffATP/EGTA, class 1); 5TAT [https://doi.org/10.2210/pdb5TAT/pdb] (Primed-state RyR1, CaffATP/EGTA, class 2); 5TAU [https://doi.org/10.2210/pdb5TAU/pdb] (Primed-state RyR1, CaffATP/EGTA, class 3); 5TAV [https://doi.org/10.2210/pdb5TAV/pdb] (Primed-state RyR1, CaffATP/EGTA, class 4); 5TAN [https://doi.org/10.2210/pdb5TAM/pdb] (Primed-state RyR1, Caffeine/ATP/Ca2+ dataset, class 3); 5TAM [https://doi.org/10.2210/pdb5TAN/pdb] (Primed-state RyR1, Caffeine/ATP/Ca2+ dataset, class 4); 5TAQ [https://doi.org/10.2210/pdb5TAQ/pdb] (Primed-state RyR1, Caffeine/ATP/Ca2+ dataset, class 3&4); 7M6A [https://doi.org/10.2210/pdb7M6A/pdb] (Primed-state RyR1, proteoliposomes); 5T9V (Activated-state RyR1, Caffeine/ATP/Ca2+ dataset, class 1); 5TA3 (Activated-state RyR1, Caffeine/ATP/Ca2+ dataset, class 2); 5TAL (Activated-state RyR1, Caffeine/ATP/Ca2+ dataset, class 1&2); 6M2W (Activated-state RyR1, Ca2 + /Caffeine/ATP/CaM1234/CHL dataset); 7CF9 (Activated-state RyR1, Ca2 + /CHL dataset); 7M6L (Activated-state RyR1, proteoliposomes); 7T65 (Activated-state RyR1, Y523S mutant); 7TDH (Activated-state RyR1, with AMP-PCP and high Ca2 + ); 5TAW [https://doi.org/10.2210/pdb5TAW/pdb] (Locked-state, ryanodine dataset, consensus); 5TAX [https://doi.org/10.2210/pdb5TAX/pdb] (Locked-state, ryanodine dataset, class 1); 5TAY [https://doi.org/10.2210/pdb5TAY/pdb] (Locked-state, ryanodine dataset, class 2); 5TAZ [https://doi.org/10.2210/pdb5TAZ/pdb] (Locked-state, ryanodine dataset, class 3); For the structures of RyR1 in native SR membrane determined in this study, atomic coordinates have been deposited in the Protein Data Bank (PDB) and cryo-EM maps in the Electron Microscopy Data Bank (EMDB) under accession codes: 9S5F [https://doi.org/10.2210/pdb9S5F/pdb] (Apo-state RyR1, EGTA-ET dataset, consensus); EMD-54597 [https://www.ebi.ac.uk/pdbe/entry/emdb/EMD-54597] (Apo-state RyR1, EGTA-ET dataset, composite map); EMD-54587 [https://www.ebi.ac.uk/pdbe/entry/emdb/EMD-54587] (Apo-state RyR1, EGTA-ET dataset, focused map); EMD-54589 [https://www.ebi.ac.uk/pdbe/entry/emdb/EMD-54589] (Apo-state RyR1, EGTA-ET dataset, focused map); EMD-54592 [https://www.ebi.ac.uk/pdbe/entry/emdb/EMD-54592] (Apo-state RyR1, EGTA-ET dataset, consensus map); EMD-54596 [https://www.ebi.ac.uk/pdbe/entry/emdb/EMD-54596] (Apo-state RyR1, EGTA-ET dataset, dimer map); 9S5A [https://doi.org/10.2210/pdb9S5A/pdb] (Apo-state RyR1, EGTA-ET dataset, “Down” class); EMD-54593 [https://www.ebi.ac.uk/pdbe/entry/emdb/EMD-54593] (Apo-state RyR1, EGTA-ET dataset, “Down” class); 9S5C [https://doi.org/10.2210/pdb9S5C/pdb] (Apo-state RyR1, EGTA-ET dataset, “Up” class); EMD-54594 [https://www.ebi.ac.uk/pdbe/entry/emdb/EMD-54594] (Apo-state RyR1, EGTA-ET dataset, “Up” class); 9S5E [https://doi.org/10.2210/pdb9S5E/pdb] (Apo-state RyR1, EGTA-ET dataset, “Tight” class); EMD-54595 [https://www.ebi.ac.uk/pdbe/entry/emdb/EMD-54595] (Apo-state RyR1, EGTA-ET dataset, “Tight” class); 9S16 [https://doi.org/10.2210/pdb9S16/pdb] (Apo-state RyR1, EGTA dataset, consensus); EMD-54445 [https://www.ebi.ac.uk/pdbe/entry/emdb/EMD-54445] (Apo-state RyR1, EGTA dataset, composite map); EMD-54431 [https://www.ebi.ac.uk/pdbe/entry/emdb/EMD-54431] (Apo-state RyR1, EGTA dataset, focused map); EMD-54433 [https://www.ebi.ac.uk/pdbe/entry/emdb/EMD-54433] (Apo-state RyR1, EGTA dataset, focused map); EMD-54435 [https://www.ebi.ac.uk/pdbe/entry/emdb/EMD-54435] (Apo-state RyR1, EGTA dataset, focused map); EMD-54437 [https://www.ebi.ac.uk/pdbe/entry/emdb/EMD-54437] (Apo-state RyR1, EGTA dataset, focused map); EMD-54438 [https://www.ebi.ac.uk/pdbe/entry/emdb/EMD-54438] (Apo-state RyR1, EGTA dataset, consensus map); 9S1H [https://doi.org/10.2210/pdb9S1H/pdb] (Apo-state RyR1, ACP dataset, consensus); EMD-54454 [https://www.ebi.ac.uk/pdbe/entry/emdb/EMD-54454] (Apo-state RyR1, ACP dataset, composite map); EMD-54450 [https://www.ebi.ac.uk/pdbe/entry/emdb/EMD-54450] (Apo-state RyR1, ACP dataset, focused map); EMD-54451 [https://www.ebi.ac.uk/pdbe/entry/emdb/EMD-54451] (Apo-state RyR1, ACP dataset, focused map); EMD-54452 [https://www.ebi.ac.uk/pdbe/entry/emdb/EMD-54452] (Apo-state RyR1, ACP dataset, focused map); EMD-54453 [https://www.ebi.ac.uk/pdbe/entry/emdb/EMD-54453] (Apo-state RyR1, ACP dataset, focused map); EMD-54449 [https://www.ebi.ac.uk/pdbe/entry/emdb/EMD-54449] (Apo-state RyR1, ACP dataset, consensus map); 9S2S [https://doi.org/10.2210/pdb9S2S/pdb] (Primed-state RyR1, ActCa dataset, consensus); EMD-54509 [https://www.ebi.ac.uk/pdbe/entry/emdb/EMD-54509] (Primed-state RyR1, ActCa dataset, composite map); EMD-54501 [https://www.ebi.ac.uk/pdbe/entry/emdb/EMD-54501] (Primed-state RyR1, ActCa dataset, focused map); EMD-54502 [https://www.ebi.ac.uk/pdbe/entry/emdb/EMD-54502] (Primed-state RyR1, ActCa dataset, focused map); EMD-54503 [https://www.ebi.ac.uk/pdbe/entry/emdb/EMD-54503] (Primed-state RyR1, ActCa dataset, focused map); EMD-54504 [https://www.ebi.ac.uk/pdbe/entry/emdb/EMD-54504] (Primed-state RyR1, ActCa dataset, focused map); EMD-54505 [https://www.ebi.ac.uk/pdbe/entry/emdb/EMD-54505] (Primed-state RyR1, ActCa dataset, consensus map); EMD-54507 [https://www.ebi.ac.uk/pdbe/entry/emdb/EMD-54507] (Primed-state RyR1, ActCa dataset, dimer map); 9S2R [https://doi.org/10.2210/pdb9S2R/pdb] (Primed-state RyR1, ActCa dataset, “Up” class); EMD-54508 [https://www.ebi.ac.uk/pdbe/entry/emdb/EMD-54508] (Primed-state RyR1, ActCa dataset, “Up” class); 9S5Z [https://doi.org/10.2210/pdb9S5Z/pdb] (Primed-state RyR1, ActMix-ET dataset, consensus); EMD-54617 [https://www.ebi.ac.uk/pdbe/entry/emdb/EMD-54617] (Primed-state RyR1, ActMix-ET dataset, composite map); EMD-54598 [https://www.ebi.ac.uk/pdbe/entry/emdb/EMD-54598] (Primed-state RyR1, ActMix-ET dataset, focused map); EMD-54600 [https://www.ebi.ac.uk/pdbe/entry/emdb/EMD-54600] (Primed-state RyR1, ActMix-ET dataset, focused map); EMD-54601 [https://www.ebi.ac.uk/pdbe/entry/emdb/EMD-54601] (Primed-state RyR1, ActMix-ET dataset, focused map); EMD-54611 [https://www.ebi.ac.uk/pdbe/entry/emdb/EMD-54611] (Primed-state RyR1, ActMix-ET dataset, focused map); EMD-54612 [https://www.ebi.ac.uk/pdbe/entry/emdb/EMD-54612] (Primed-state RyR1, ActMix-ET dataset, consensus map); EMD-54614 [https://www.ebi.ac.uk/pdbe/entry/emdb/EMD-54614] (Primed-state RyR1, ActMix-ET dataset, dimer map); 9S5W [https://doi.org/10.2210/pdb9S5W/pdb] (Primed-state RyR1, ActMix-ET dataset, “Down” class); EMD-54615 [https://www.ebi.ac.uk/pdbe/entry/emdb/EMD-54615] (Primed-state RyR1, ActMix-ET dataset, “Down” class); 9S5Y [https://doi.org/10.2210/pdb9S5Y/pdb] (Primed-state RyR1, ActMix-ET dataset, “Up” class); EMD-54616 [https://www.ebi.ac.uk/pdbe/entry/emdb/EMD-54616] (Primed-state RyR1, ActMix-ET dataset, “Up” class); 9S3V [https://doi.org/10.2210/pdb9S3V/pdb] (Primed-state RyR1, ActMix dataset, consensus); EMD-54553 [https://www.ebi.ac.uk/pdbe/entry/emdb/EMD-54553] (Primed-state RyR1, ActMix dataset, composite map); EMD-54512 [https://www.ebi.ac.uk/pdbe/entry/emdb/EMD-54512] (Primed-state RyR1, ActMix dataset, focused map); EMD-54513 [https://www.ebi.ac.uk/pdbe/entry/emdb/EMD-54513] (Primed-state RyR1, ActMix dataset, focused map); EMD-54514 [https://www.ebi.ac.uk/pdbe/entry/emdb/EMD-54514] (Primed-state RyR1, ActMix dataset, focused map); EMD-54515 [https://www.ebi.ac.uk/pdbe/entry/emdb/EMD-54515] (Primed-state RyR1, ActMix dataset, focused map); EMD-54516 [https://www.ebi.ac.uk/pdbe/entry/emdb/EMD-54516] (Primed-state RyR1, ActMix dataset, consensus map); EMD-54517 [https://www.ebi.ac.uk/pdbe/entry/emdb/EMD-54517] (Activated and Primed-state RyR1, ActMix dataset, dimer map); 9S3U [https://doi.org/10.2210/pdb9S3U/pdb] (Primed-state RyR1, ActMix dataset, “Down” class); EMD-54552 [https://www.ebi.ac.uk/pdbe/entry/emdb/EMD-54552] (Primed-state RyR1, ActMix dataset, “Down class”); 9S4N [https://doi.org/10.2210/pdb9S4N/pdb] (Activated-state RyR1, ActMix dataset, consensus); EMD-54570 [https://www.ebi.ac.uk/pdbe/entry/emdb/EMD-54570] (Activated-state RyR1, ActMix dataset, composite map); EMD-54554 [https://www.ebi.ac.uk/pdbe/entry/emdb/EMD-54554] (Activated-state RyR1, ActMix dataset, focused map); EMD-54555 [https://www.ebi.ac.uk/pdbe/entry/emdb/EMD-54555] (Activated-state RyR1, ActMix dataset, focused map); EMD-54557 [https://www.ebi.ac.uk/pdbe/entry/emdb/EMD-54557] (Activated-state RyR1, ActMix dataset, focused map); EMD-54562 [https://www.ebi.ac.uk/pdbe/entry/emdb/EMD-54562] (Activated-state RyR1, ActMix dataset, focused map); EMD-54568 [https://www.ebi.ac.uk/pdbe/entry/emdb/EMD-54568] (Activated-state RyR1, ActMix dataset, consensus map); 9S4L [https://doi.org/10.2210/pdb9S4L/pdb] (Activated-state RyR1, ActMix dataset, “Down” class); EMD-54569 [https://www.ebi.ac.uk/pdbe/entry/emdb/EMD-54569] (Activated-state RyR1, ActMix dataset, “Down class”); 9S55 [https://doi.org/10.2210/pdb9S55/pdb] (Locked-state RyR1, Ryanodine dataset, consensus); EMD-54590 [https://www.ebi.ac.uk/pdbe/entry/emdb/EMD-54590] (Locked-state RyR1, Ryanodine dataset, composite map); EMD-54571 [https://www.ebi.ac.uk/pdbe/entry/emdb/EMD-54571] (Locked-state RyR1, Ryanodine dataset, focused map); EMD-54572 [https://www.ebi.ac.uk/pdbe/entry/emdb/EMD-54572] (Locked-state RyR1, Ryanodine dataset, focused map); EMD-54573 [https://www.ebi.ac.uk/pdbe/entry/emdb/EMD-54573] (Locked-state RyR1, Ryanodine dataset, focused map); EMD-54574 [https://www.ebi.ac.uk/pdbe/entry/emdb/EMD-54574] (Locked-state RyR1, Ryanodine dataset, focused map); EMD-54575 [https://www.ebi.ac.uk/pdbe/entry/emdb/EMD-54575] (Locked-state RyR1, Ryanodine dataset, consensus map); EMD-54580 [https://www.ebi.ac.uk/pdbe/entry/emdb/EMD-54580] (Locked-state RyR1, Ryanodine dataset, dimer map); 9S54 [https://doi.org/10.2210/pdb9S54/pdb] (Locked-state RyR1, Ryanodine dataset, “Down” class); EMD-54588 [https://www.ebi.ac.uk/pdbe/entry/emdb/EMD-54588] (Locked-state RyR1, Ryanodine dataset, “Down” class); The raw cryo‑EM image data supporting this study have been deposited in the Electron Microscopy Public Image Archive (EMPIAR) under accession codes: EMPIAR‑13487 [https://doi.org/10.6019/EMPIAR-13487] (EGTA dataset); EMPIAR‑13647 [https://doi.org/10.6019/EMPIAR-13647] (ACP dataset); EMPIAR‑13646 [https://doi.org/10.6019/EMPIAR-13646] (ActCa dataset); EMPIAR-13783 [https://doi.org/10.6019/EMPIAR-13783] (ActMix dataset); EMPIAR-13784 [https://doi.org/10.6019/EMPIAR-13784] (Ryanodine dataset); EMPIAR-13785 [https://doi.org/10.6019/EMPIAR-13785] (EGTA-ET dataset); EMPIAR-13786 [https://doi.org/10.6019/EMPIAR-13786] (ActMix-ET dataset).

## Code availability

The code for calculating multidimensional scaling is available at [https://github.com/KudryashevLab/RMSDistance]. The archived version is available under the following reference: Mikirtumov, V. & Kudryashev, M. (2026). RMSDistance: The release for the publication (Version v1.0.1) [Computer software]. Zenodo. https://doi.org/10.5281/zenodo.21395997.
