# Supplementary material for: Ligand-induced activation of RyR1 in native membranes
Source: Nat Commun. 2026 Sep 24;17:10075. doi: 10.1038/s41467-026-75504-9 (PMC13612463; doi:10.1038/s41467-026-75504-9)
Supplement: Supplementary file 1 [file 41467_2026_75504_MOESM1_ESM.pdf]

SUPPLEMENTAL INFORMATION FOR

Ligand-induced activation of RyR1 in native membranes

Vasilii Mikirtumov[1,2,†], Sabrina Golusik[1], Ruifeng Huo[1,2], Thiemo Sprink[3,4], Nikita Balyschew[5], Wen Yang[6], Christoph Diebolder[3,4], Shuguang Yuan[7,8], Abhay Kotecha[6,9] & Mikhail Kudryashev[1,10*]

1. *In situ* Structural Biology, Max Delbrück Center for Molecular Medicine in the Helmholtz Association (MDC), Berlin, Germany.

2. Institute of Chemistry and Biochemistry, Freie Universität Berlin, Takustraße 6, 14195 Berlin, Germany

3. Charité-Universitätsmedizin Berlin, corporate member of Freie Universität Berlin and Humboldt Universität zu Berlin, Core Facility for cryo-EM, Berlin, Germany

4. Technology Platform for Cryo-EM, Max Delbrück Center for Molecular Medicine in the Helmholtz Association (MDC), Berlin, Germany

5. Max Planck Institute of Biophysics, Frankfurt am Main, Germany

6. Materials and Structural Analysis Division, Thermo Fisher Scientific, Eindhoven, The Netherlands.

7. AlphaMol Science Ltd, 200120 Shanghai, China

8. Research Center for Medicinal Structural Biology, National Research Center for Translational Medicine at Shanghai, State Key Laboratory of Medical Genomics, Ruijin Hospital, Shanghai Jiao Tong University School of Medicine, Shanghai, 200025, China

9. Present address: Center of Excellence for Structural Cell Biology, Department of Structural Biology, St. Jude Children's Research Hospital, Memphis, TN 38105, USA

10. Institute of Medical Physics and Biophysics, Charité-Universitätsmedizin Berlin, Germany.

†Present address: Institute of Medical Physics and Biophysics, Charité-Universitätsmedizin Berlin, Germany.
*Correspondence to: M.K. (mikhail.kudryashev@mdc-berlin.de)

This file contains:
Supplementary Tables 1-9
Supplementary Figures 1-36

Supplementary Table 1. Overview of RyR1 structures in native membranes.

Summary of datasets, ligand conditions, method, state assignment, pore status, particle numbers, and resolutions for each RyR1 conformation.

Dataset	EGTA Dataset 1	EGTA Dataset 2	EGTA/ACP	Activating [free Ca^{2+}]	Ca^{2+}/ACP/caffeine ("Activation Mix")			Ryanodine
EMPIAR ID	EMPIAR-13785	EMPIAR-13487	EMPIAR-13647	EMPIAR-13646	EMPIAR-13783		EMPIAR-13786	EMPIAR-13784
Dataset Short Name	EGTA-ET	EGTA	ACP	ActCa	ActMix		ActMix-ET	Ryanodine
Condition	5 mM EGTA	5 mM EGTA	2 mM ACP	30 uM Ca^{2+}	30 uM Ca^{2+}<br>2 mM ACP<br>5 mM caffeine			5 uM Ryanodine
Names	Apo1-4	Apo5	ACP	ActCa1-2	ActMix1-2	ActMix3-4	ActMix5-7	Ry1-2
Method	StA	SPA	SPA	SPA	SPA		StA	SPA
State	Apo	Apo	Apo	Primed	Primed	Activated	Primed	Locked
Pore	closed	closed	closed	closed	closed	open	closed	dilated
Number of particles	13,726	25,786	27,861	82,421	69,502	37,771	13,275	157,550
Global resolution	4.6 Å	3.7 Å	4 Å	3.9 Å	3.5 Å	3.8 Å	4.9 Å	3.8 Å
Resolution shell1/shell2/core/TMD	4 - 4 4 Å*	3.7 4.7 3.9 3.6 Å	3.9 5.1 4.2 4.1 Å	3.8 4.7 4 3.8 Å	3.3 4.1 3.5 3.2 Å	3.7 5 3.8 3.5 Å	4.4 5.5 4.5 4.4 Å	3.7 4.5 3.8 3.7 Å
Structures/ Classes +%	Consensus, "Down" 32%, "Up" 26%, "Tight" 8%	Consensus,-	Consensus,-	Consensus,"Up" 14.6%	Consensus, "Down" 27.5%	Consensus, "Down" 43.3%	Consensus,"Down" 25%, "Up" 11.5%	Consensus, "Down" 33%
EMDB ID	EMD-54597, EMD-54593,	EMD-54445	EMD-54454	EMD-54509, EMD-54508	EMD-54553, EMD-54552	EMD-54570, EMD-54569	EMD-54617, EMD-54615,	EMD-54590, EMD-54588

	EMD-54594, EMD-54595						EMD-54616	
PDB ID	9S5F, 9S5A, 9S5C, 9S5E	9S16	9S1H	9S2S, 9S2R	9S3V, 9S3U	9S4N, 9S4L	9S5Z, 9S5W, 9S5Y	9S55, 9S54
pO	0	0	0	0	0.35		0**	1
Cα aperture Diameter, Å	11.3 Å	11.4 Å	11.2 Å	11.1 Å	10.6 Å	19 Å	11.3 Å	20.1 Å
Dimer map resolution, Å	26 Å	-	-	11.7 Å	18.4 Å		20.0 Å	13.1 Å
Dimer map, number. of particles	256	-	-	36,833	16,161		1,035	44,350
Dimer map EMDB ID	EMD-54596	-	-	EMD-54507	EMD-54517		EMD-54614	EMD-54580

* Only two focused refinements of the Activation Core-TMD and N-terminal cytosolic shell parts resulted in better reconstructions.

** No open-pore classes were obtained by 3D classification, possibly due to lower overall particle count.

Supplementary Table 2. Statistical data for the analysis of the clustering of the same-class and same-state channels related to Fig. 4 (a-f). Statistics: non-parametric permutation tests on the fixed particle–neighbor graph; one-sided (testing enrichment of same-class neighbors); 10,000 permutations. For each class, the test statistic is the mean fraction of neighbors sharing the same class label across channels in that class with at least one neighbor, and P values are the proportion of permutations with a test statistic ≥ the observed value. No adjustment for multiple comparisons was applied.

Dataset	State	Observed value	n (particles)	Null mean ± standard deviation	One-tailed P-value	Class	Observed value	n (particles)	Null mean ± standard deviation	One-tailed P-value
EGTA-ET	Apo	-	668	-	-	Down	0.72	407	0.59 ± 0.03	0
						Up	0.3	187	0.29 ± 0.05	0.411
						Tight	0.56	74	0.12 ± 0.05	0
ActMix-ET	Primed	-	1908	-	-	Down	0.43	623	0.31 ± 0.03	0
						Up	0.23	247	0.13 ± 0.03	0.0009
ActMix	Activated	0.4	861	0.36 ± 0.02	0.018	Up	0.16	307	0.13 ± 0.03	0.102
						Down	0.18	299	0.13 ± 0.03	0.027
	Primed	0.65	1495	0.64 ± 0.01	0.048	Up	0.41	877	0.37 ± 0.02	0.016
						Down	0.11	216	0.09 ± 0.03	0.271
ActCa	Primed	-	20994	-	-	Down	0.57	11678	0.55 ± 0.01	0
						Up	0.2	3102	0.15 ± 0.001	0
Ryanodine	Locked	-	44024	-	-	Up	0.42	16387	0.37 ± 0.004	0
						Down	0.35	14624	0.33 ± 0.005	0

Supplementary Table 3. Analysis of the RyR1-RyR1 dimer interfaces by PISA after interface sidechain optimization with ISOLDE.

Dataset	Dimer State	Interface Area, Å^2	Number of interfacing residues
EGTA	Apo	512	44
Activating [Ca^{2+}]	Primed	602	43
Activation Mix	Primed	121	16
Activation Mix	Primed-Activated	242	23
Activation Mix	Activated	256	22
Activation Mix [cryo-ET]	Primed	174	24
Ryanodine	Locked	984	69

Supplementary Table 4. Potential energy values after energy minimization and statistical analysis. Statistics: Two-sided independent-samples t tests were used for pairwise comparisons between single-channel and dimer potential energies within each functional state, and one-way, two-sided ANOVAs followed by Tukey's Honest Significant Difference (HSD) post-hoc tests were used for comparisons of potential energy across functional states. Tukey HSD P values are multiplicity-adjusted; all other P values are unadjusted. Exact test statistics, degrees of freedom, effect sizes, and P values are reported for each comparison.

Dataset	Functional state	Potential energy for the single channel, kJ/mol (replicas of 3)	Dimer functional state	Potential energy for the dimer, kJ/mol (replicas of 3)	Single channel versus dimer difference within the group
EGTA-StA	Apo	-1.31E+06, -1.35E+06, -1.30E+06	Apo-Apo	-2.82E+06, -2.81E+06, -2.81E+06	Significant, t-statistic: -5.64, p-value: 0.0049
ActCa	Primed	-1.38E+06, -1.33E+06, -1.37E+06	Primed-Primed	-2.88E+06, -2.77E+06, -2.89E+06	t-statistic: -2.58, p-value: 0.061
ActMix	Primed	-1.39E+06, -1.41E+06, -1.36E+06	Primed-Primed	-2.98E+06, -2.83E+06, -2.81E+06	t-statistic: -1.64, p-value: 0.176
			Primed-Activated	-2.82E+06, -2.69E+06, -2.83E+06	Significant, t-statistic: -4.50, p-value: 0.01
	Activated	-1.15E+06, -1.16E+06, -1.23E+06	Activated-Activated	-2.60E+06, -2.45E+06, -2.51E+06	t-statistic: -2.40, p-value: 0.07
Ryanodine	Locked	-1.36E+06, -1.37E+06, -1.37E+06	Locked-Locked	-2.78E+06, -2.82E+06, -2.81E+06	Significant, t-statistic: -5.09, p-value: 0.007

Supplementary Table 5. Comparison of the cytosolic shell tilt angle values measured in this study with the flexion angle values available from the literature.

Model (PDB ID)	flexion angle, reported previously (reference)	Cytosolic shell tilt, this study
5tal	-3.8, estimated (Steele and Samso, JSB, 2019)	-4.1
5tb0	1.7 estimated (Steele and Samso, JSB, 2019)	1.57
7tdj	-3.6 (Nayak and Samso, eLife, 2022)	-3.52
7tdi	-4.4 (Nayak and Samso, eLife, 2022)	-4.51
7tdk	-5.6 (Nayak and Samso, eLife, 2022)	-5.44
7tdh	-5.1 (Nayak and Samso, eLife, 2022)	-5.53
7k0t	-2.2 (Nayak and Samso, eLife, 2022)	-2.64

Supplementary Table 6. Cryo-EM data collection, refinement, and validation statistics for the EGTA-ET dataset, EMPIAR-13785.

	Consensus, PDB 9S5F, EMD-54597	“Down”-class, PDB 9S5A, EMD-54593	“Up”-class, PDB 9S5C, EMD-54594	“Tight”-class, PDB 9S5E, EMD-54595
Data collection and processing				
Magnification	64000x			
Voltage (kV)	300			
Electron exposure ($e^-/Å^2$)	132			
Defocus range (μm)	-1.8 to -3.8			
Pixel size (calibrated, Å)	0.974			

Symmetry imposed	C4			
Initial particle images (no.)	144900			
Final particle images (no.)	13726	4402	3555	1088
Consensus map resolution (Å)	4.6	6	6	7
Consensus map resolution range (Å)	4.1 – 15	5.8 – 19.3	5.8 – 17.4	5.8 – 26.5
Refinement				
Initial model used (PDB code)	7TZC			
Model resolution (Å)	4.1/3.4*	5.9/5.7	5.9/5.8	6.8/5.7
FSC threshold	0.5/0.143			
Consensus map sharpening B factor ($Å^2$)	-101	-200	-200	-200
Model composition				
Nonhydrogen atoms	285968	285964	285964	285964
Protein residues	18044			
Ligands	ZN: 4	-	-	-
B factors ($Å^2$)				
Protein	139.94	123.49	123.49	123.49
Ligand	71.29	-	-	-
R.m.s.d.				
Bond lengths (Å)	0.004	0.012	0.012	0.013
Bond angles (°)	0.937	2.031	2.061	2.153

Validation				
MolProbity score	1.60	1.93	2.03	2.17
Clashscore	3.75	1.68	1.78	2.07
Poor rotamers (%)	1.46	5.14	6.35	7.54
Ramachandran plot				
Favored (%)	95.54	92.41	91.99	90.90
Allowed (%)	4.46	6.54	6.66	7.76
Disallowed (%)	0.00	1.05	1.35	1.34

Supplementary Table 7. Cryo-EM data collection, refinement, and validation statistics for the ActMix-ET dataset, EMPIAR-13786.

	Consensus, PDB 9S5Z, EMD-54617	“Down”-class, PDB 9S5W, EMD-54615	“Up”-class, PDB 9S5Y, EMD-54616
Data collection and processing			
Magnification	53000x		
Voltage (kV)	300		
Electron exposure ($e^-/Å^2$)	117		
Defocus range (μm)	-2 to -7		
Pixel size (Å)	1.68		
Symmetry imposed	C4		
Initial particle images (no.)	525283		
Final particle images (no.)	13356	3346	1541
Consensus map resolution (Å)	4.9	4.9	6.8
Consensus map resolution	4.2 – 13.5	4.2 – 14.6	4.8 – 16.5

range (Å)			
Refinement			
Initial model used (PDB code)	7TZC		
Model resolution (Å)	4.5/3.8	4.8/4.2	7.0/4.4
FSC threshold	0.5/0.143		
Consensus map sharpening B factor (Å^2)	-111	-110	-115
Model composition			
Nonhydrogen atoms	286244	285964	285964
Protein residues	18044		
Ligands	CFF: 4<br>ZN: 4<br>CA: 4<br>ACP: 4	ZN: 4	ZN: 4
B factors (Å^2)			
Protein	59.96	106.65	161.32
Ligand	119.33	71.29	71.29
R.m.s.d.			
Bond lengths (Å)	0.010	0.004	0.004
Bond angles (°)	1.102	0.931	0.949
Validation			
MolProbity score	1.43	1.48	1.71
Clashscore	2.93	2.74	3.98
Poor rotamers (%)	0.00	0.00	0.00
Ramachandran plot			
Favored (%)	94.93	93.67	90.84

Allowed (%)	5.00	6.22	8.98
Disallowed (%)	0.07	0.11	0.18

Supplementary Table 8. Cryo-EM data collection, refinement, and validation statistics for the EGTA (EMPIAR-13487), ACP (EMPIAR-13647), and ActCa (EMPIAR-13646) SPA datasets.

	Apo/EGTA-Consensus, PDB 9S16, EMD-54445	Apo/ACP-Consensus, PDB 9S1H, EMD-54454	Primed/ActCa-Consensus, PDB 9S2S, EMD-54509	Primed/ActCa-"Up"-class, PDB 9S2R, EMD-54508
Data collection and processing				
Magnification	64000x			
Voltage (kV)	300			
Electron exposure (e$^-$/Å^2)	60.9	59.9	60.9	
Defocus range (μm)	-0.5 to -4			
Pixel size (calibrated, Å)	1.366			
Symmetry imposed	C4			
Initial particle images (no.)	1480995	2031101	1655849	
Final particle images (no.)	25786	27861	82421	12029
Consensus map resolution (Å)	3.7	4	3.8	4.2
Consensus map resolution range (Å)	3 – 10.1	3.6 – 10.1	3.5 – 9	3 – 10.2
Refinement				
Initial model used (PDB code)	7TZC			
Model resolution (Å)	3.8/2.8	4.1/2.9	3.9/2.9	4.7/4.2
FSC threshold	0.5/0.143			
Consensus map sharpening B factor (Å^2)	-45.9	-54.6	-85.8	-34.2
Model composition				

Nonhydrogen atoms	287016	286124	287020	285948
Protein residues	18044			
Ligands	ZN: 4<br>POV: 8	ZN: 4<br>ACP: 4	ZN: 4<br>POV: 8<br>CA: 4	ZN: 4<br>CA: 4
B factors (Å^2)				
Protein	109.41	87.65	74.44	223.99
Ligand	1.17	82.95	1.45	50.83
R.m.s.d.				
Bond lengths (Å)	0.010	0.009	0.009	0.003
Bond angles (°)	1.129	1.082	1.098	0.909
Validation				
MolProbity score	1.15	1.34	1.10	1.11
Clashscore	1.90	2.49	1.83	1.57
Poor rotamers (%)	0.94	1.10	0.59	0.00
Ramachandran plot				
Favored (%)	96.85	96.01	97.09	96.70
Allowed (%)	3.13	3.95	2.88	3.21
Disallowed (%)	0.02	0.04	0.02	0.09

Supplementary Table 9. Cryo-EM data collection, refinement, and validation statistics for the ActMix (EMPIAR-13783) and Ryanodine (EMPIAR-13784) SPA datasets.

	Primed/ActMix-Consensus, PDB 9S3V, EMD-54553	Primed/ActMix-"Down"-class, PDB 9S3U, EMD-54552	Activated/ActMix-Consensus, PDB 9S4N, EMD-54570	Activated/ActMix-"Down"-class, PDB 9S4L, EMD-54569	Locked/Ryanodine-Consensus, PDB 9S55, EMD-54590	Locked/Ryanodine-"Down"-class, PDB 9S54, EMD-54588
Data collection and processing						
Magnification	64000x					
Voltage (kV)	300					
Electron exposure (e^-/Å^2)	67.44					
Defocus range (μm)	-0.5 to -4					
Pixel size (calibrated, Å)	1.366					
Symmetry imposed	C4					
Initial particle images (no.)	2288891				2171564	
Final particle images (no.)	69502	19082	37771	16369	157550	52057
Consensus map resolution (Å)	3.5	4.8	3.8	4.9	3.8	4
Consensus map resolution range (Å)	3 – 9.1	4.2 – 18.4	3 – 9.9	4.2 – 19.5	3 – 8.8	3.5 – 9.4
Refinement						
Initial model used (PDB code)	7TZC					
Model resolution (Å)	3.4/2.2	4.7/4.0	3.8/3.0	6.7/4.1	3.8/2.4	5.0/4.1
FSC threshold	0.5/0.143					

Consensus map sharpening B factor (Å^2)	-60.6	-	-44.4	-	-104.2	-69.6
Model composition						
Nonhydrogen atoms	287304	285944	287300	285960	287024	285952
Protein residues	18044					
Ligands	CFF: 4<br>POV: 8<br>ZN: 4<br>CA: 4<br>ACP: 4	-	CFF: 4<br>POV: 8<br>ZN: 4<br>CA: 4<br>ACP: 4	-	POV: 8<br>ZN: 4	ZN: 4
B factors (Å^2)						
Protein	59.54	79.98	128.95	132.33	57.59	57.59
Ligand	14.81	-	17.85	-	1.17	71.29
R.m.s.d.						
Bond lengths (Å)	0.012	0.012	0.012	0.012	0.013	0.012
Bond angles (°)	1.962	1.948	1.930	1.970	1.986	1.922
Validation						
MolProbity score	0.83	1.20	0.99	1.34	1.00	1.15
Clashscore	0.11	0.24	0.12	0.29	0.15	0.16
Poor rotamers (%)	1.00	2.04	1.58	2.76	1.37	2.02
Ramachandran plot						
Favored (%)	95.81	94.50	95.73	94.05	95.15	94.83
Allowed (%)	3.92	5.06	3.80	5.35	4.50	4.75
Disallowed (%)	0.27	0.44	0.47	0.60	0.36	0.42

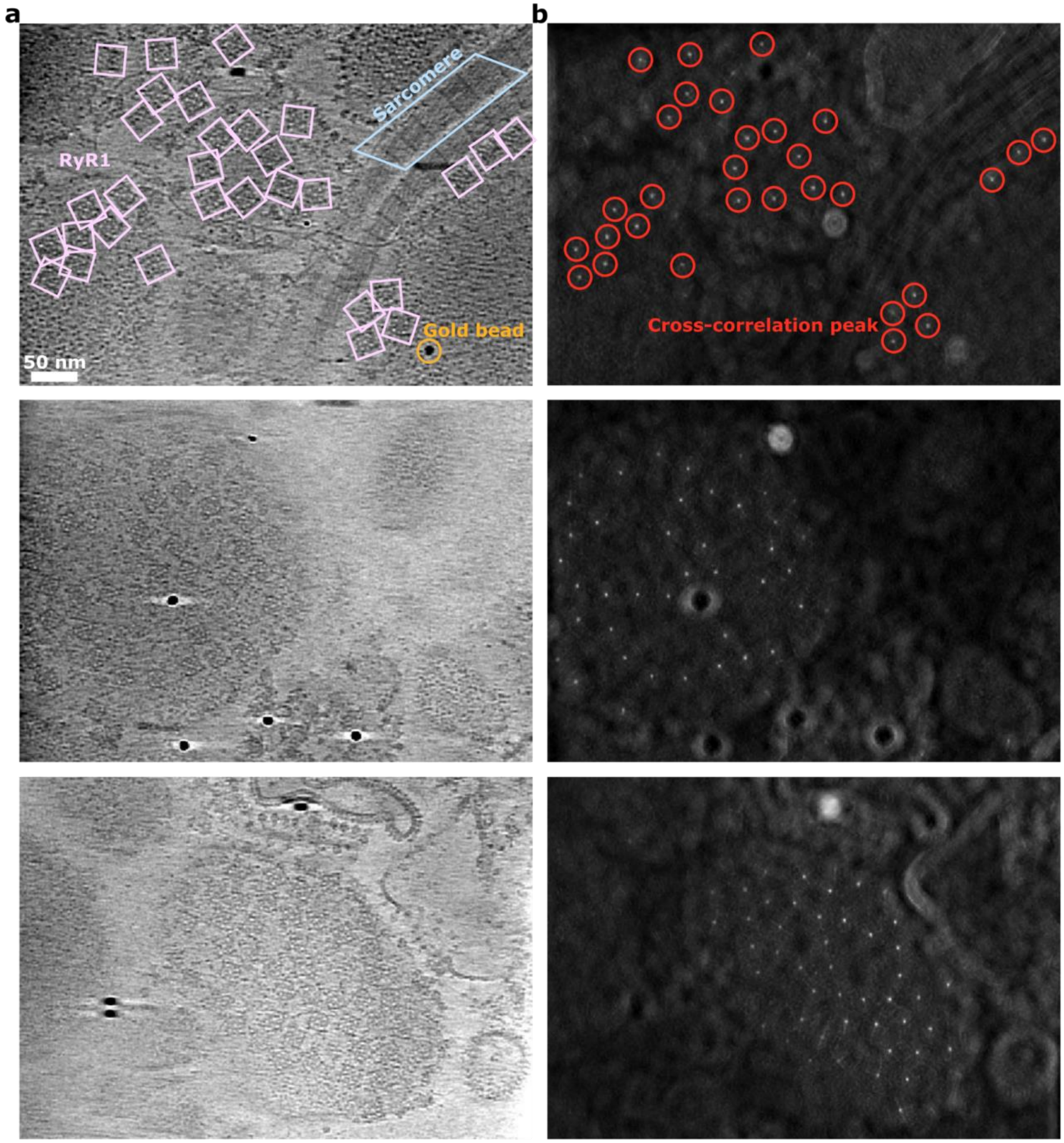

Supplementary Figure 1. RyR1 arrays on the SR vesicle surface visualized by cryo-ET.

- **a.** Representative tomogram slices showing SR vesicles with RyR1 arrays on the surface. Slices of regions from 3 out of 182 tomograms from the ActMix-ET dataset are shown.
- **b.** The corresponding slices through the cross-correlation volumes after template matching; the bright white peaks show RyR1 localization.

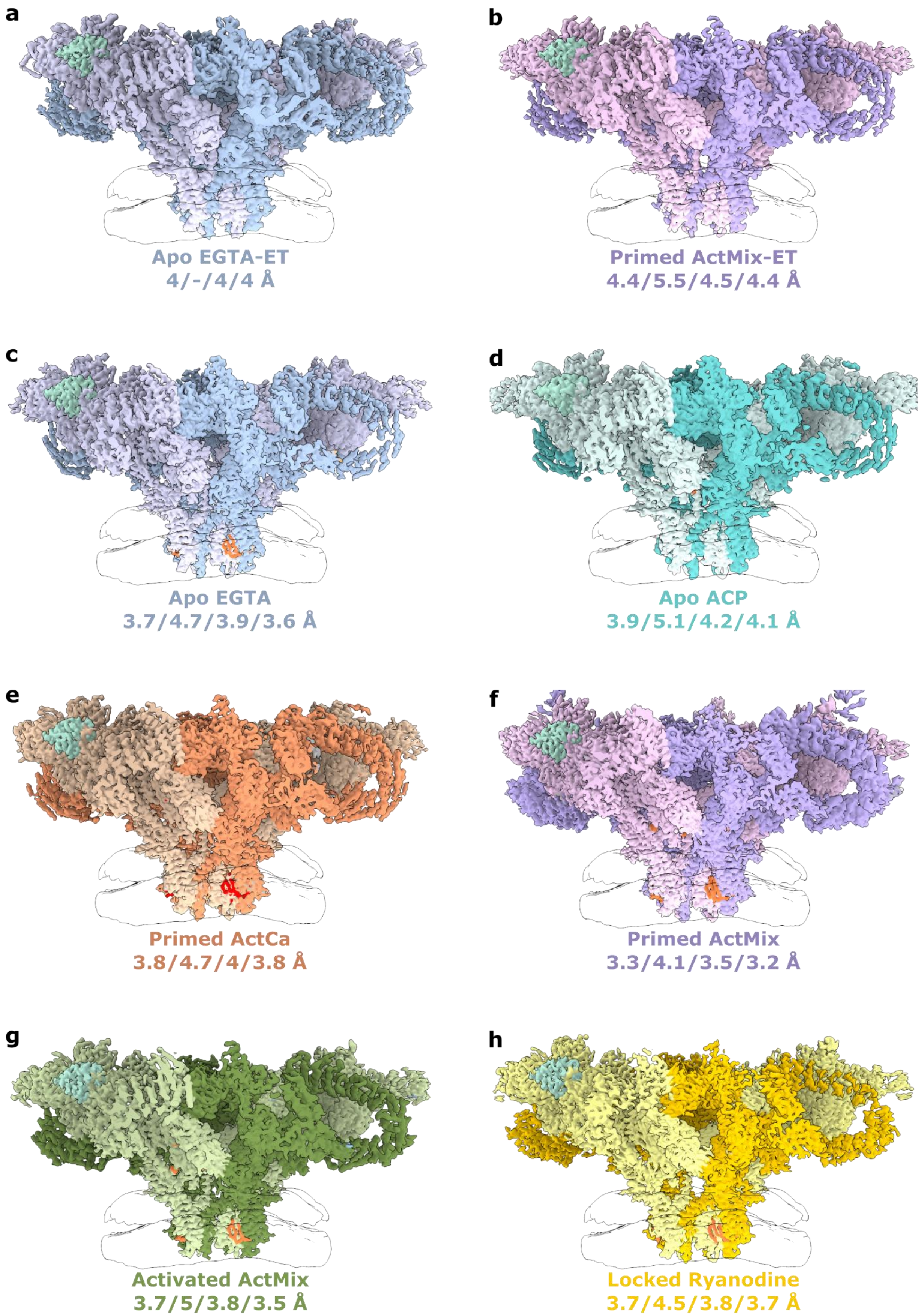
a
Apo EGTA-ET
4/-/4/4 Å
b
Primed ActMix-ET
4.4/5.5/4.5/4.4 Å
c
Apo EGTA
3.7/4.7/3.9/3.6 Å
d
Apo ACP
3.9/5.1/4.2/4.1 Å
e
Primed ActCa
3.8/4.7/4/3.8 Å
f
Primed ActMix
3.3/4.1/3.5/3.2 Å
g
Activated ActMix
3.7/5/3.8/3.5 Å
h
Locked Ryanodine
3.7/4.5/3.8/3.7 Å

Supplementary Figure 2. Density maps of RyR1 determined by SPA and StA.

- **a.** Composite map of the Apo-state structure obtained from the EGTA-ET dataset. Resolutions correspond to the individual focused parts, namely N-terminal cytosolic shell, BSol, JSol, and CSol activation core and TMD, TaF, and CTD parts, respectively.
- **b.** Composite map of the Primed-state with Ca^{2+}/ACP/caffeine from the ActMix-ET dataset.
- **c.** Composite map of the Apo-state structure obtained from the EGTA dataset.
- **d.** Composite map of the Apo-state in complex with ACP.
- **e.** Composite map of the Primed-state with Activating [Ca^{2+}].
- **f.** Composite map of the Primed-state with Ca^{2+}/ACP/caffeine.
- **g.** Composite map of the Activated-state with Ca^{2+}/ACP/caffeine.
- **h.** Composite map of the Locked-state with ryanodine.

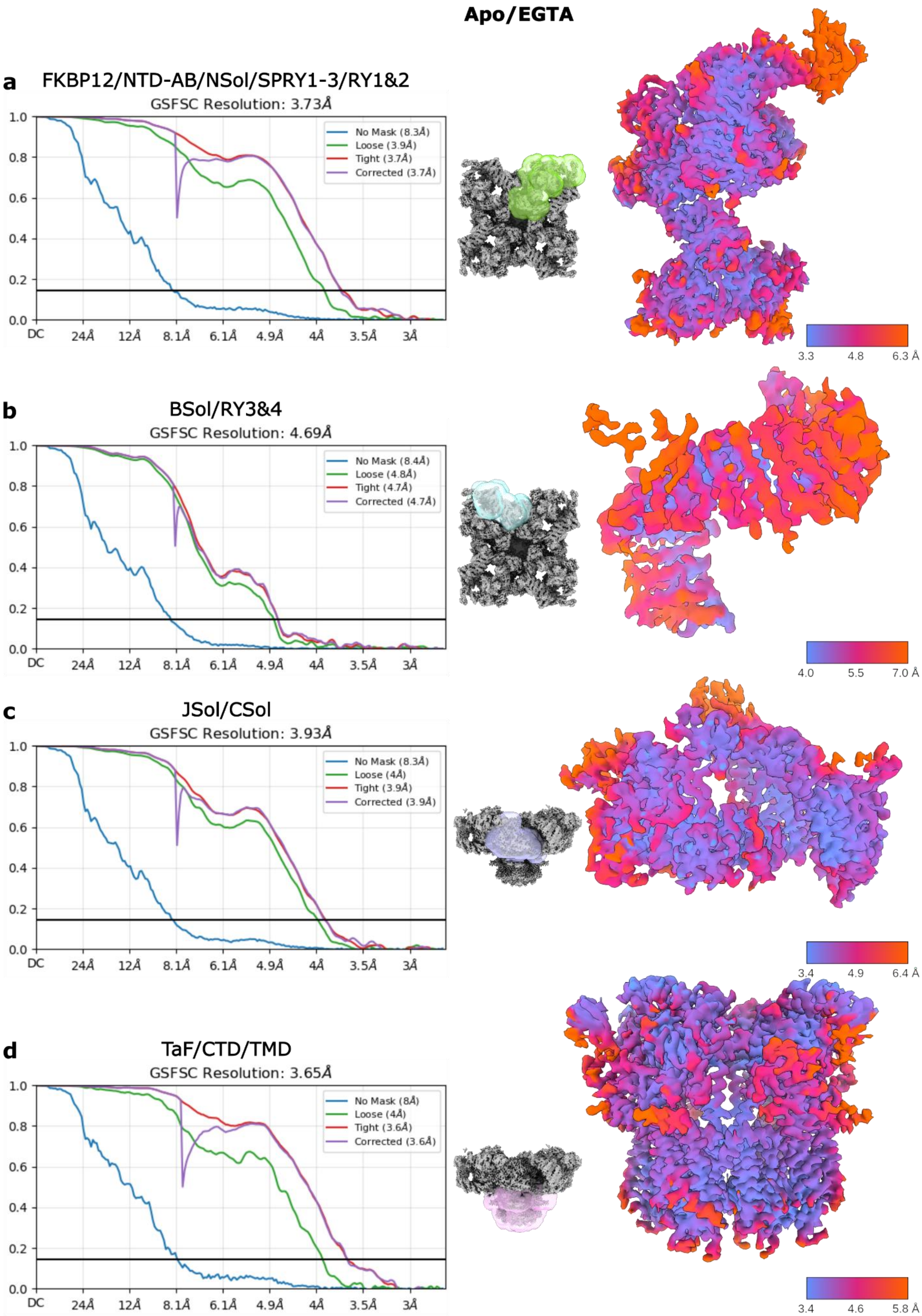
Apo/EGTA
a FKBP12/NTD-AB/NSol/SPRY1-3/RY1&2
GSFSC Resolution: 3.73Å
No Mask (8.3Å)
Loose (3.9Å)
Tight (3.7Å)
Corrected (3.7Å)
1.0 0.8 0.6 0.4 0.2 0.0
DC 24Å 12Å 8.1Å 6.1Å 4.9Å 4Å 3.5Å 3Å
3.3 4.8 6.3 Å
b BSol/RY3&4
GSFSC Resolution: 4.69Å
No Mask (8.4Å)
Loose (4.8Å)
Tight (4.7Å)
Corrected (4.7Å)
1.0 0.8 0.6 0.4 0.2 0.0
DC 24Å 12Å 8.1Å 6.1Å 4.9Å 4Å 3.5Å 3Å
4.0 5.5 7.0 Å
c JSol/CSol
GSFSC Resolution: 3.93Å
No Mask (8.3Å)
Loose (4Å)
Tight (3.9Å)
Corrected (3.9Å)
1.0 0.8 0.6 0.4 0.2 0.0
DC 24Å 12Å 8.1Å 6.1Å 4.9Å 4Å 3.5Å 3Å
3.4 4.9 6.4 Å
d TaF/CTD/TMD
GSFSC Resolution: 3.65Å
No Mask (8Å)
Loose (4Å)
Tight (3.6Å)
Corrected (3.6Å)
1.0 0.8 0.6 0.4 0.2 0.0
DC 24Å 12Å 8.1Å 6.1Å 4.9Å 4Å 3.5Å 3Å
3.4 4.6 5.8 Å

Supplementary Figure 3. Focused refinement of the Apo-state/EGTA dataset.

a. The FSC curve and a map colored according to the local resolution, and the mask for the N-terminal cytosolic shell part, comprising FKBP12, NTDA-B, NSol, SPRY1-3, and RY1&2 domains, performed on the symmetry-expanded particle set.

b. The FSC curve and a map colored according to the local resolution, and the mask for the BSol cytosolic shell part, comprising BSol and RY3&4 domains, performed on the symmetry-expanded particle set.

c. The FSC curve and a map colored according to the local resolution, and the mask for the Activation core part, comprising JSol and CSol domains, performed on the symmetry-expanded particle set.

d. The FSC curve and a map colored according to the local resolution and the mask for the TMD part, comprising TaF, S2S3, pVSD, Pore, and CTD domains, performed with an imposed C4 symmetry.

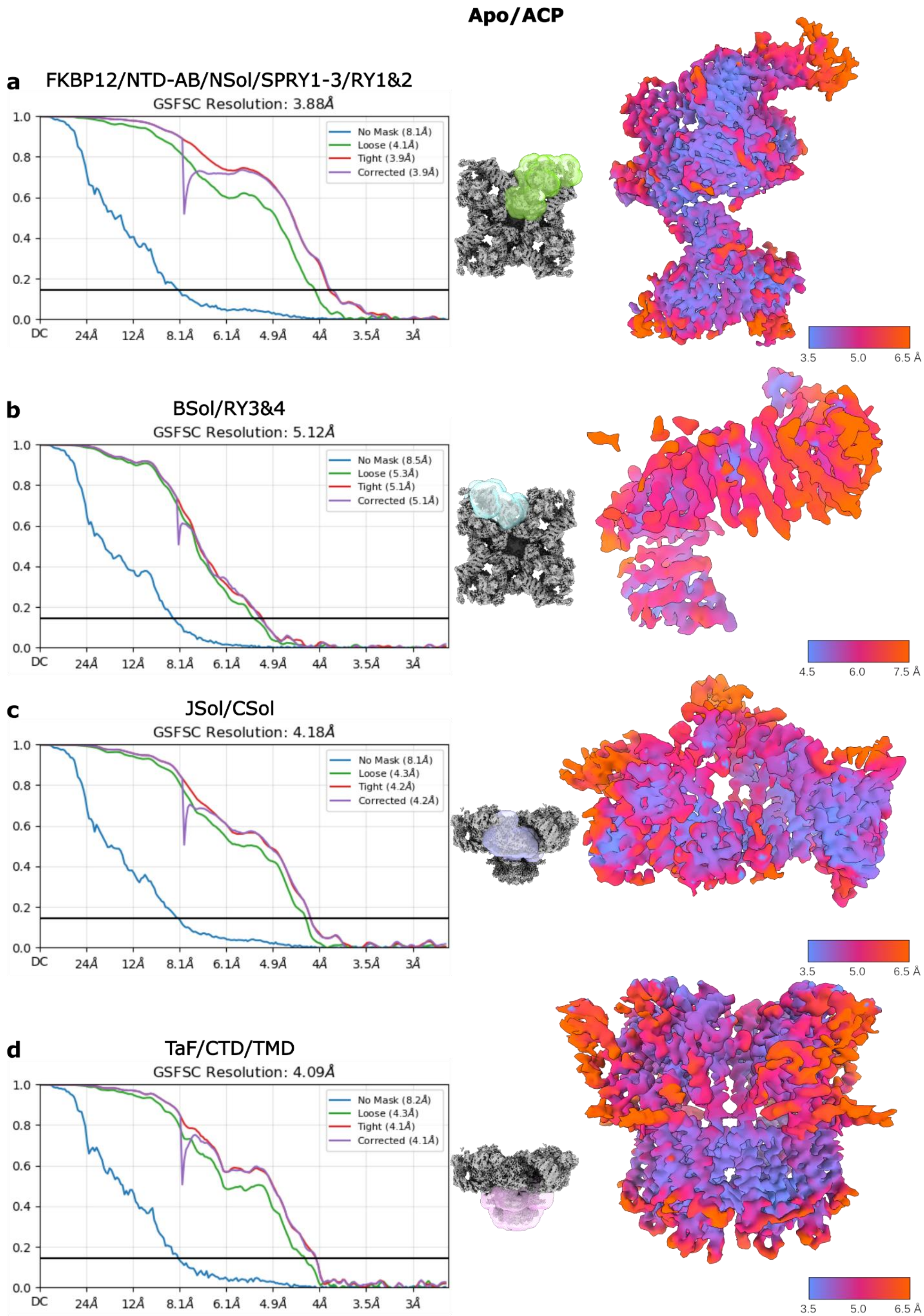
Apo/ACP
a
FKBP12/NTD-AB/NSol/SPRY1-3/RY1&2
GSFSC Resolution: 3.88Å
No Mask (8.1Å)
Loose (4.1Å)
Tight (3.9Å)
Corrected (3.9Å)
3.5 5.0 6.5 Å
b
BSol/RY3&4
GSFSC Resolution: 5.12Å
No Mask (8.5Å)
Loose (5.3Å)
Tight (5.1Å)
Corrected (5.1Å)
4.5 6.0 7.5 Å
c
JSol/CSol
GSFSC Resolution: 4.18Å
No Mask (8.1Å)
Loose (4.3Å)
Tight (4.2Å)
Corrected (4.2Å)
3.5 5.0 6.5 Å
d
TaF/CTD/TMD
GSFSC Resolution: 4.09Å
No Mask (8.2Å)
Loose (4.3Å)
Tight (4.1Å)
Corrected (4.1Å)
3.5 5.0 6.5 Å
DC 24Å 12Å 8.1Å 6.1Å 4.9Å 4Å 3.5Å 3Å

Supplementary Figure 4. Focused refinement of the Apo-state/ACP dataset.

a. The FSC curve and a map colored according to the local resolution and the mask for the N-terminal cytosolic shell part, comprising FKBP12, NTDA-B, NSol, SPRY1-3, and RY1&2 domains, performed on the symmetry-expanded particle set.

b. The FSC curve and a map colored according to the local resolution, and the mask for the BSol cytosolic shell part, comprising BSol and RY3&4 domains, performed on the symmetry-expanded particle set.

c. The FSC curve and a map colored according to the local resolution, and the mask for the Activation core part, comprising JSol and CSol domains, performed on the symmetry-expanded particle set.

d. The FSC curve and a map colored according to the local resolution, and the mask for the TMD part, comprising TaF, S2S3, pVSD, Pore, and CTD domains, performed with an imposed C4 symmetry.

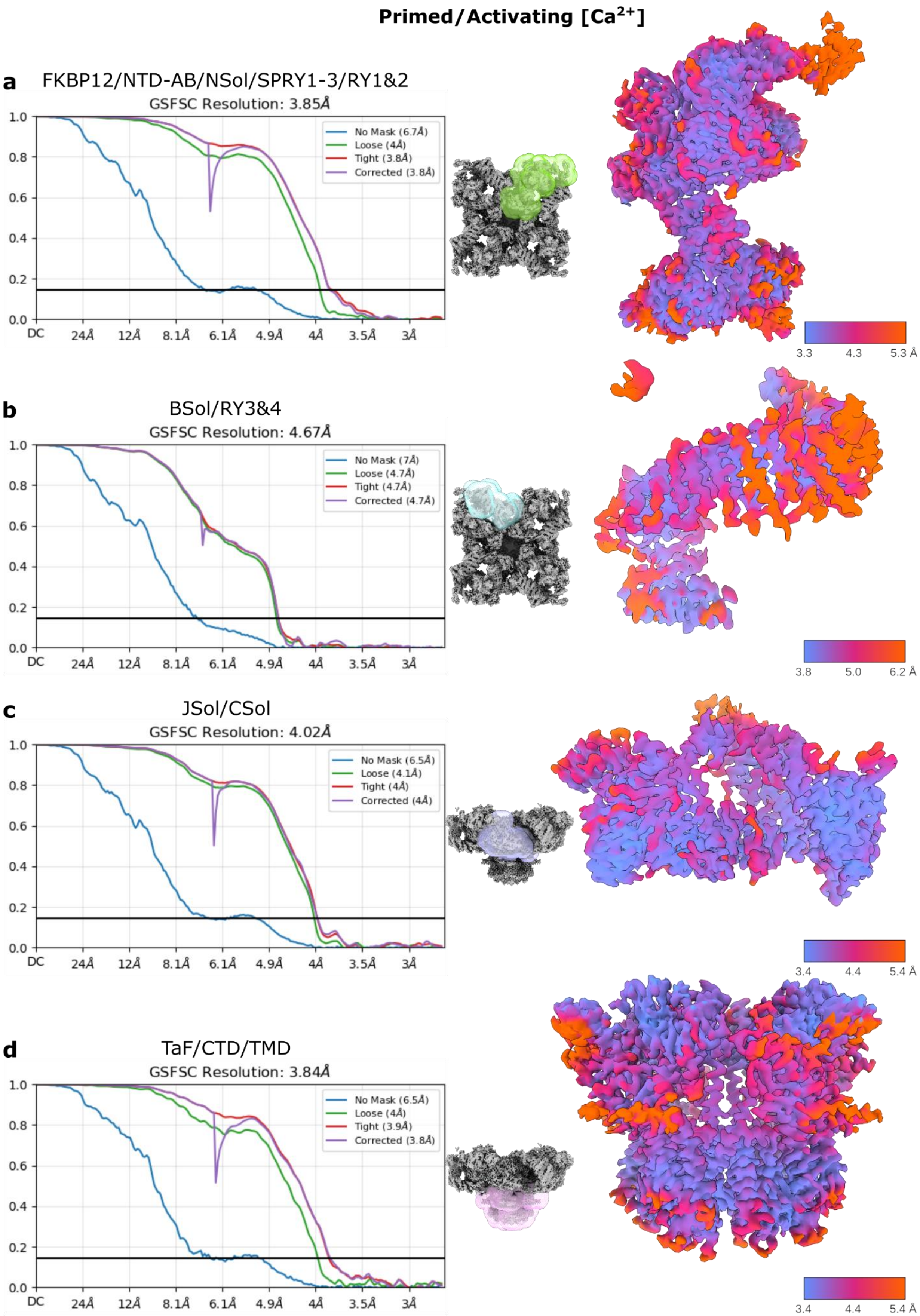
Primed/Activating [Ca2+]
a
FKBP12/NTD-AB/NSol/SPRY1-3/RY1&2
GSFSC Resolution: 3.85Å
No Mask (6.7Å)
Loose (4Å)
Tight (3.8Å)
Corrected (3.8Å)
1.0
0.8
0.6
0.4
0.2
0.0
DC
24Å
12Å
8.1Å
6.1Å
4.9Å
4Å
3.5Å
3Å
3.3
4.3
5.3 Å
b
BSol/RY3&4
GSFSC Resolution: 4.67Å
No Mask (7Å)
Loose (4.7Å)
Tight (4.7Å)
Corrected (4.7Å)
3.8
5.0
6.2 Å
c
JSol/CSol
GSFSC Resolution: 4.02Å
No Mask (6.5Å)
Loose (4.1Å)
Tight (4Å)
Corrected (4Å)
3.4
4.4
5.4 Å
d
TaF/CTD/TMD
GSFSC Resolution: 3.84Å
No Mask (6.5Å)
Loose (4Å)
Tight (3.9Å)
Corrected (3.8Å)
3.4
4.4
5.4 Å

Supplementary Figure 5. Focused refinement of the Primed-state/Activating [Ca^{2+}] dataset.

- **a.** The FSC curve and a map colored according to the local resolution and the mask for the N-terminal cytosolic shell part, comprising FKBP12, NTDA-B, NSol, SPRY1-3, and RY1&2 domains, performed on the symmetry-expanded particle set.
- **b.** The FSC curve and a map colored according to the local resolution, and the mask for the BSol cytosolic shell part, comprising BSol and RY3&4 domains, performed on the symmetry-expanded particle set.
- **c.** The FSC curve and a map colored according to the local resolution, and the mask for the Activation core part, comprising JSol and CSol domains, performed on the symmetry-expanded particle set.
- **d.** The FSC curve and a map colored according to the local resolution, and the mask for the TMD part, comprising TaF, S2S3, pVSD, Pore, and CTD domains, performed with an imposed C4 symmetry.

Primed/ActMix

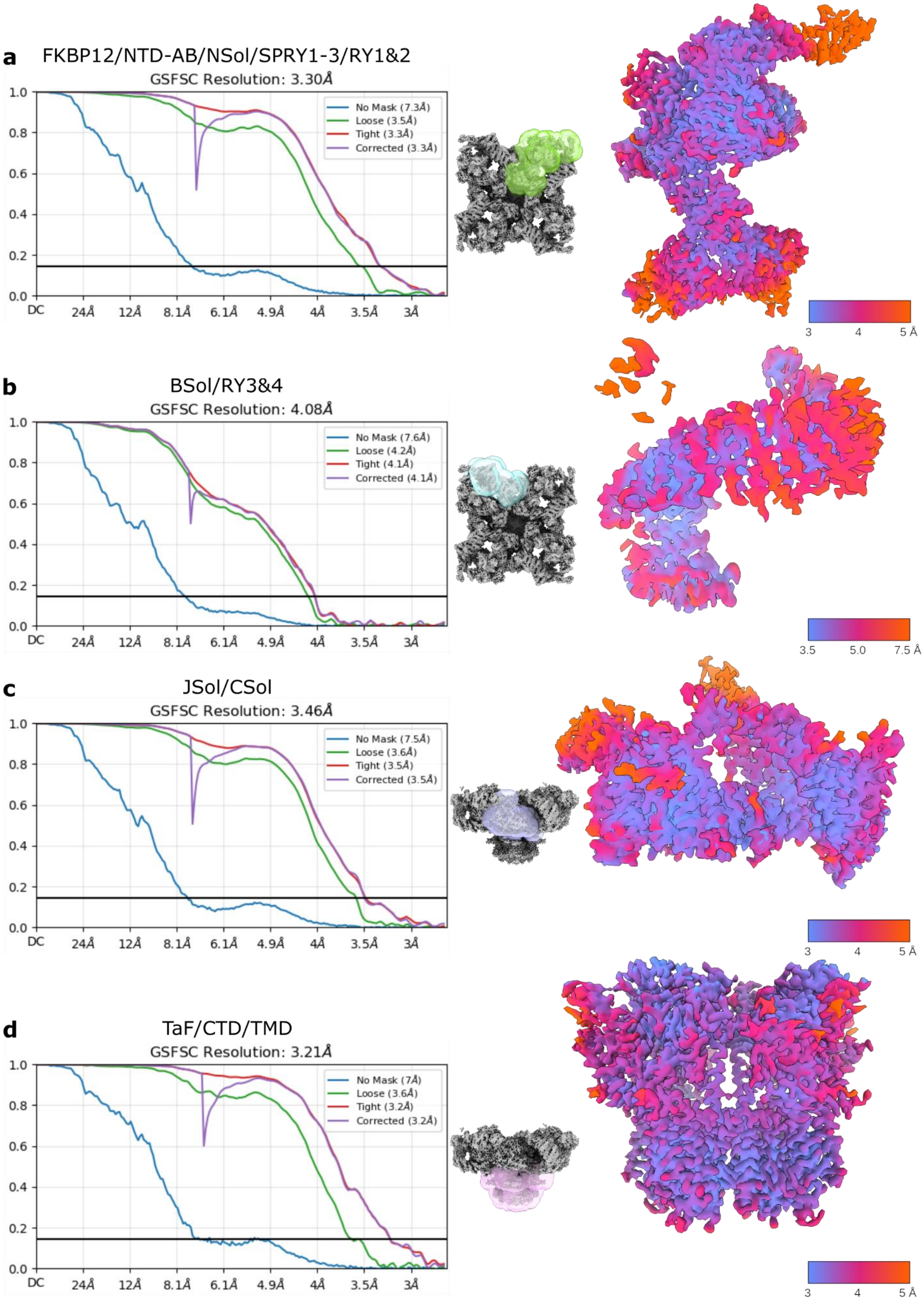

Supplementary Figure 6. Focused refinement of the Primed-state/Activation Mixture Ca^{2+}/ACP/caffeine dataset.

a. The FSC curve and a map colored according to the local resolution and the mask for the N-terminal cytosolic shell part, comprising FKBP12, NTDA-B, NSol, SPRY1-3, and RY1&2 domains, performed on the symmetry-expanded particle set.

b. The FSC curve and a map colored according to the local resolution, and the mask for the BSol cytosolic shell part, comprising BSol and RY3&4 domains, performed on the symmetry-expanded particle set.

c. The FSC curve and a map colored according to the local resolution, and the mask for the Activation core part, comprising JSol and CSol domains, performed on the symmetry-expanded particle set.

d. The FSC curve and a map colored according to the local resolution and the mask for the TMD part, comprising TaF, S2S3, pVSD, Pore, and CTD domains, performed with an imposed C4 symmetry.

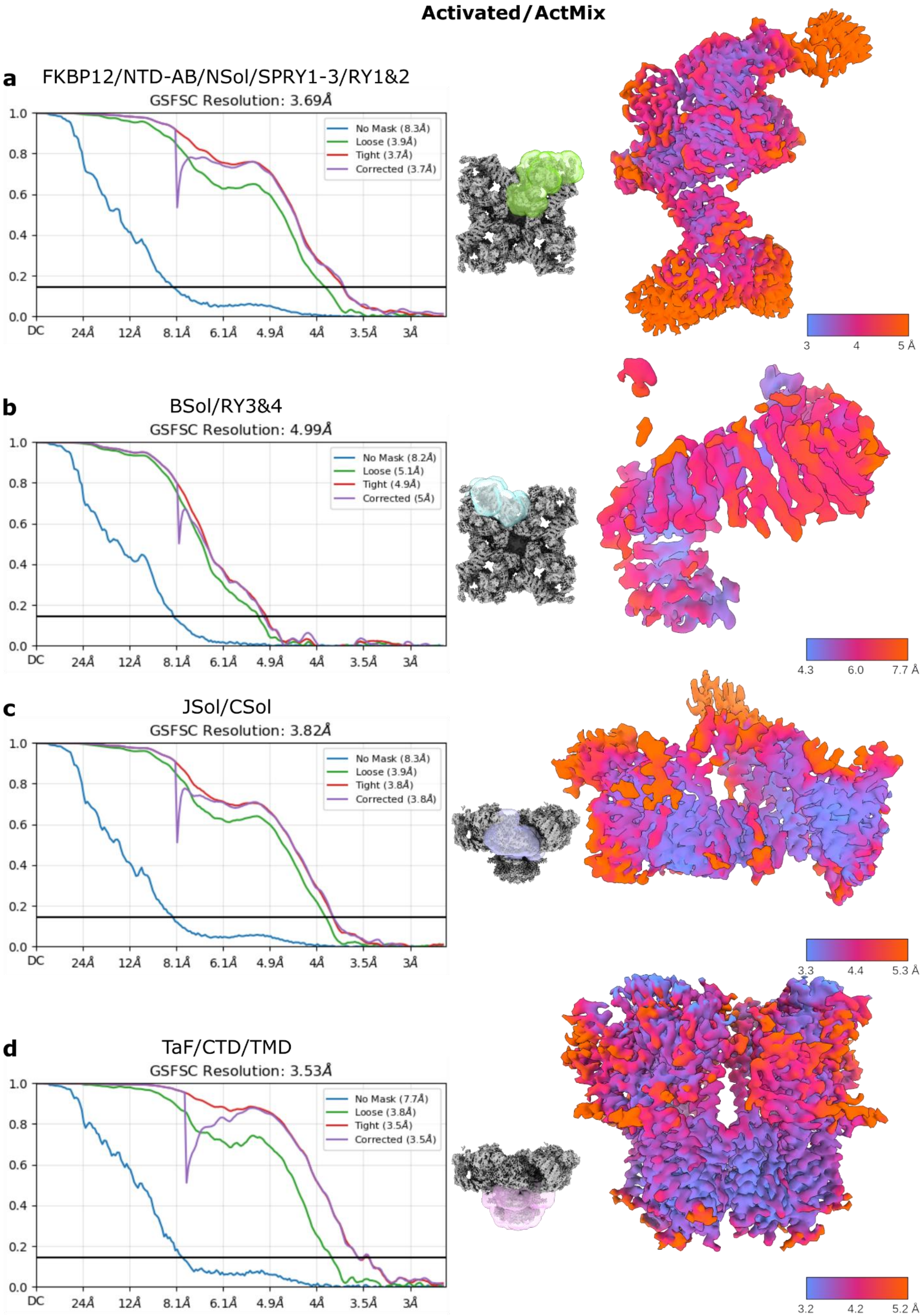
Activated/ActMix
a
FKBP12/NTD-AB/NSol/SPRY1-3/RY1&2
GSFSC Resolution: 3.69Å
No Mask (8.3Å)
Loose (3.9Å)
Tight (3.7Å)
Corrected (3.7Å)
1.0
0.8
0.6
0.4
0.2
0.0
DC
24Å
12Å
8.1Å
6.1Å
4.9Å
4Å
3.5Å
3Å
3
4
5 Å
b
BSol/RY3&4
GSFSC Resolution: 4.99Å
No Mask (8.2Å)
Loose (5.1Å)
Tight (4.9Å)
Corrected (5Å)
4.3
6.0
7.7 Å
c
JSol/CSol
GSFSC Resolution: 3.82Å
No Mask (8.3Å)
Loose (3.9Å)
Tight (3.8Å)
Corrected (3.8Å)
3.3
4.4
5.3 Å
d
TaF/CTD/TMD
GSFSC Resolution: 3.53Å
No Mask (7.7Å)
Loose (3.8Å)
Tight (3.5Å)
Corrected (3.5Å)
3.2
4.2
5.2 Å

Supplementary Figure 7. Focused refinement of the Activated-state/Activation Mixture Ca^{2+}/ACP/caffeine dataset.

a. The FSC curve and map colored according to the local resolution and the mask for the N-terminal cytosolic shell part, comprising FKBP12, NTDA-B, NSol, SPRY1-3, and RY1&2 domains, performed on the symmetry-expanded particle set.

b. The FSC curve and a map colored according to the local resolution, and the mask for the BSol cytosolic shell part, comprising BSol and RY3&4 domains, performed on the symmetry-expanded particle set.

c. The FSC curve and a map colored according to the local resolution, and the mask for the Activation core part, comprising JSol and CSol domains, performed on the symmetry-expanded particle set.

d. The FSC curve and a map colored according to the local resolution, and the mask for the TMD part, comprising TaF, S2S3, pVSD, Pore, and CTD domains, performed with an imposed C4 symmetry.

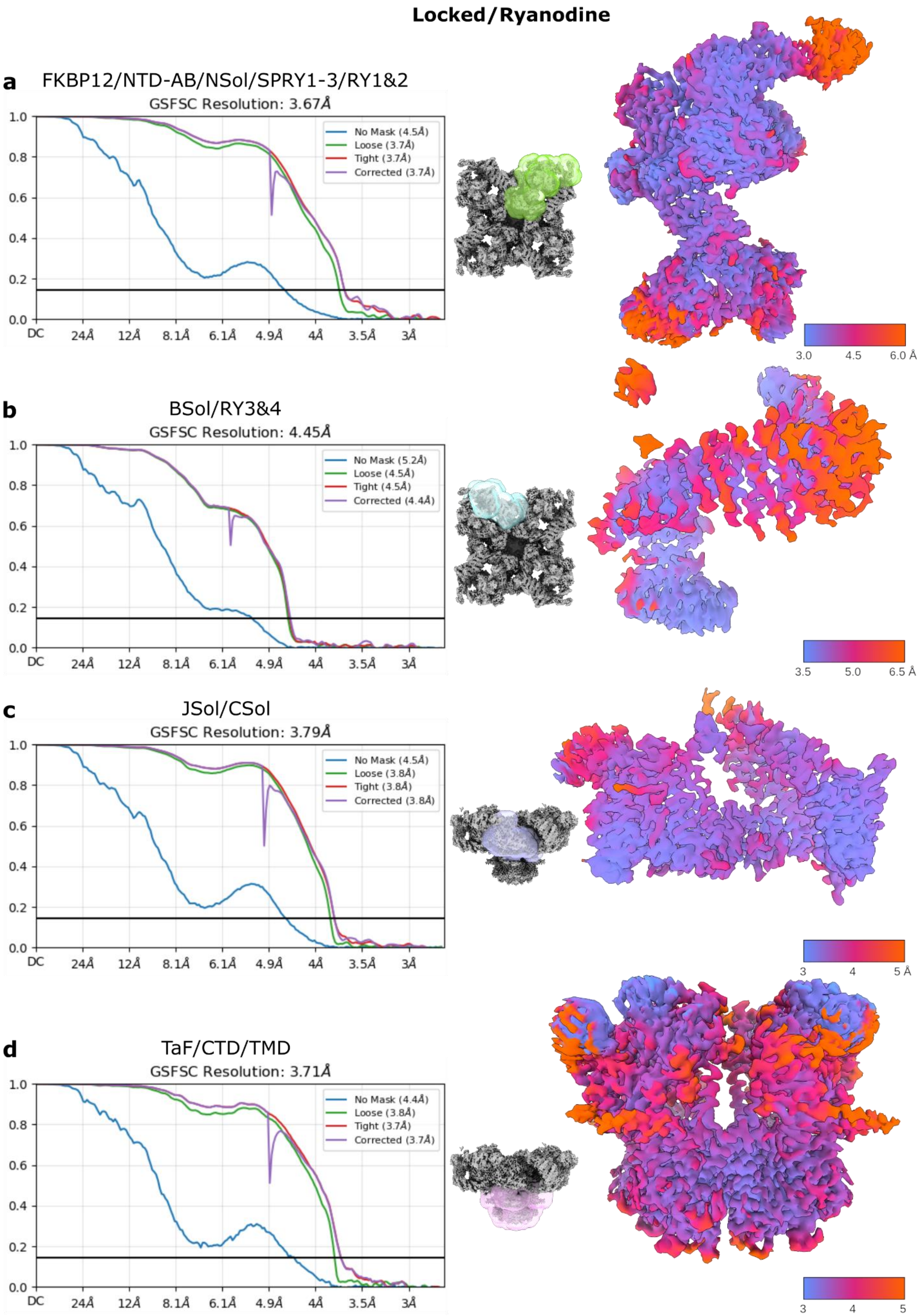

Locked/Ryanodine
a
FKBP12/NTD-AB/NSol/SPRY1-3/RY1&2
GSFSC Resolution: 3.67Å
No Mask (4.5Å)
Loose (3.7Å)
Tight (3.7Å)
Corrected (3.7Å)
3.0
4.5
6.0 Å
b
BSol/RY3&4
GSFSC Resolution: 4.45Å
No Mask (5.2Å)
Loose (4.5Å)
Tight (4.5Å)
Corrected (4.4Å)
3.5
5.0
6.5 Å
c
JSol/CSol
GSFSC Resolution: 3.79Å
No Mask (4.5Å)
Loose (3.8Å)
Tight (3.8Å)
Corrected (3.8Å)
3
4
5 Å
d
TaF/CTD/TMD
GSFSC Resolution: 3.71Å
No Mask (4.4Å)
Loose (3.8Å)
Tight (3.7Å)
Corrected (3.7Å)
3
4
5
1.0
0.8
0.6
0.4
0.2
0.0
DC
24Å
12Å
8.1Å
6.1Å
4.9Å
4Å
3.5Å
3Å

Supplementary Figure 8. Focused refinement of the Locked-state/Ryanodine dataset.

a. The FSC curve and a map colored according to the local resolution and the mask for the N-terminal cytosolic shell part, comprising FKBP12, NTDA-B, NSol, SPRY1-3, and RY1&2 domains, performed on the symmetry-expanded particle set.

b. The FSC curve and a map colored according to the local resolution, and the mask for the BSol cytosolic shell part, comprising BSol and RY3&4 domains, performed on the symmetry-expanded particle set.

c. The FSC curve and a map colored according to the local resolution, and the mask for the Activation core part, comprising JSol and CSol domains, performed on the symmetry-expanded particle set.

d. The FSC curve and a map colored according to the local resolution, and the mask for the TMD part, comprising TaF, S2S3, pVSD, Pore, and CTD domains, performed with an imposed C4 symmetry.

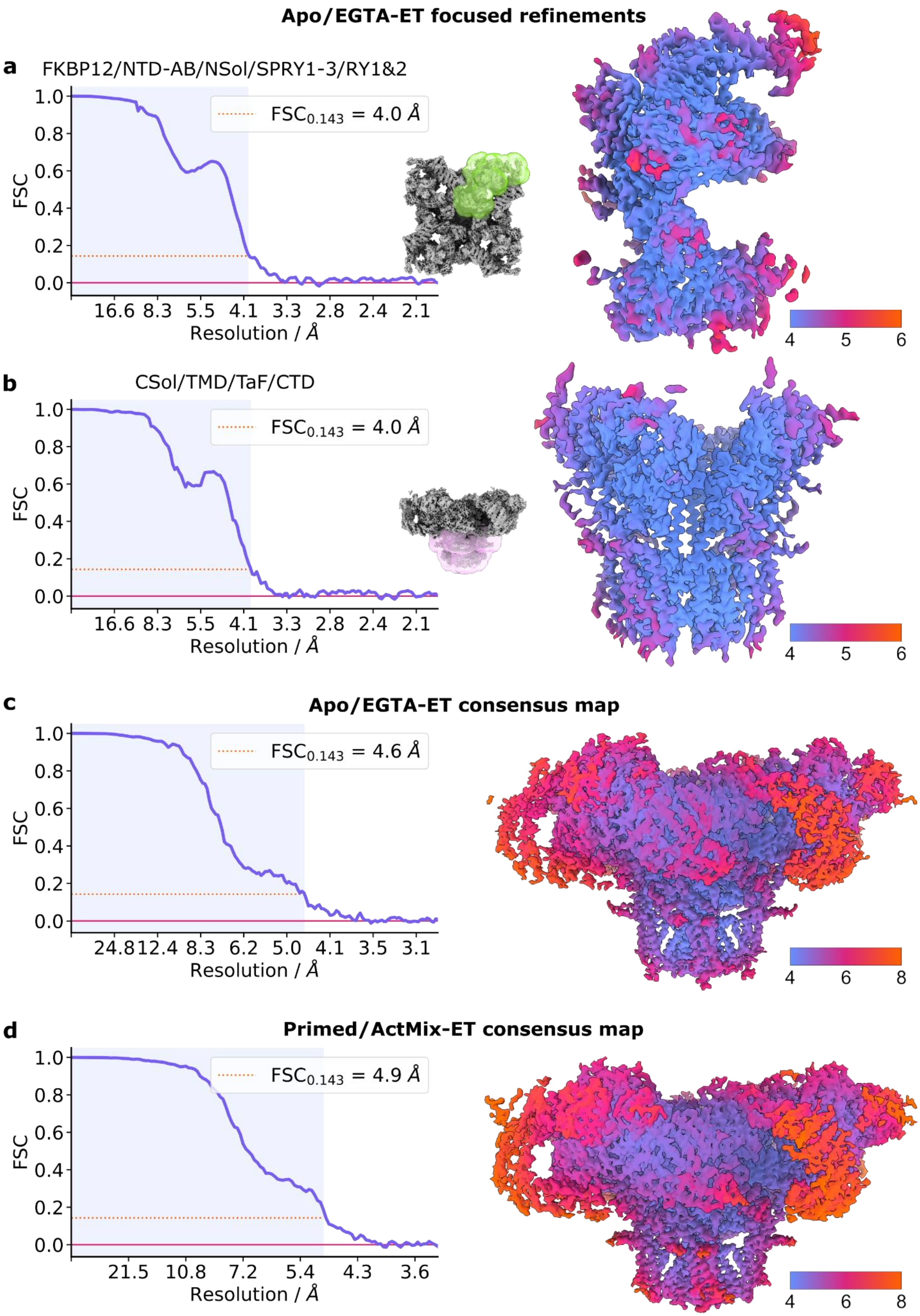
Apo/EGTA-ET focused refinements
a
FKBP12/NTD-AB/NSol/SPRY1-3/RY1&2
FSC
1.0
0.8
0.6
0.4
0.2
0.0
FSC$_{0.143}$ = 4.0 Å
16.6 8.3 5.5 4.1 3.3 2.8 2.4 2.1
Resolution / Å
4 5 6
b
CSol/TMD/TaF/CTD
FSC
1.0
0.8
0.6
0.4
0.2
0.0
FSC$_{0.143}$ = 4.0 Å
16.6 8.3 5.5 4.1 3.3 2.8 2.4 2.1
Resolution / Å
4 5 6
c
Apo/EGTA-ET consensus map
FSC
1.0
0.8
0.6
0.4
0.2
0.0
FSC$_{0.143}$ = 4.6 Å
24.8 12.4 8.3 6.2 5.0 4.1 3.5 3.1
Resolution / Å
4 6 8
d
Primed/ActMix-ET consensus map
FSC
1.0
0.8
0.6
0.4
0.2
0.0
FSC$_{0.143}$ = 4.9 Å
21.5 10.8 7.2 5.4 4.3 3.6
Resolution / Å
4 6 8

Supplementary Figure 9. Focused refinement of the Apo-state/EGTA-ET dataset and consensus maps from the EGTA-ET and the ActMix-ET datasets.

- **a.** The FSC curve and a map colored according to the local resolution and the mask for the N-terminal cytosolic shell part, comprising FKBP12, NTDA-B, NSol, SPRY1-3, and RY1&2 domains, performed on the symmetry-expanded particle set.
- **b.** The FSC curve and a map colored according to the local resolution and the mask for the CSol and TMD part, comprising CSol, TaF, S2S3, pVSD, Pore, and CTD domains, performed with an imposed C4 symmetry.
- **c.** The FSC curve and the consensus map colored according to the local resolution for the EGTA-ET dataset.
- **d.** The FSC curve and the consensus map colored according to the local resolution for the ActMix-ET dataset.

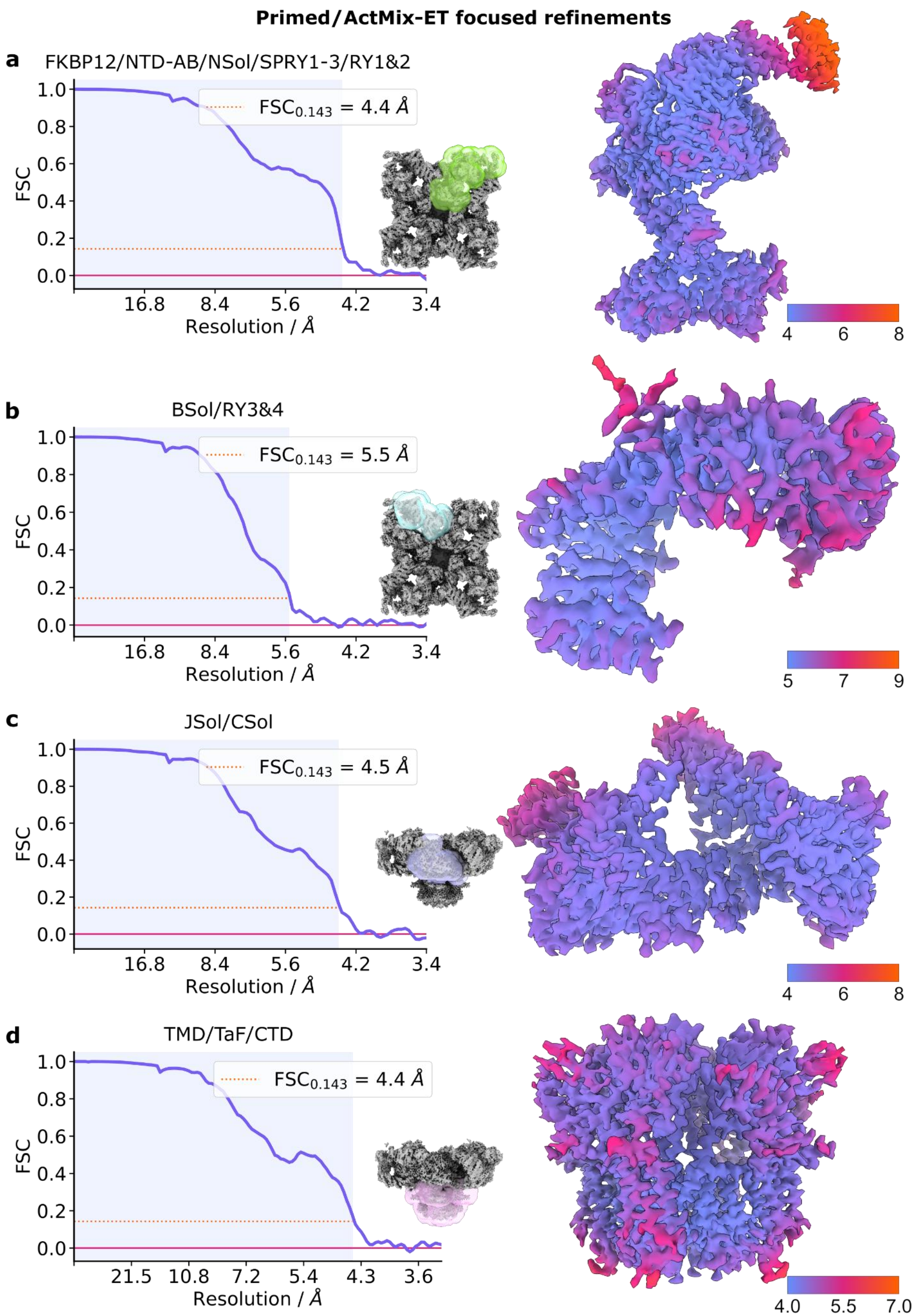
Primed/ActMix-ET focused refinements
a
FKBP12/NTD-AB/NSol/SPRY1-3/RY1&2
FSC0.143 = 4.4 Å
FSC
Resolution / Å
16.8 8.4 5.6 4.2 3.4
4 6 8
b
BSol/RY3&4
FSC0.143 = 5.5 Å
5 7 9
c
JSol/CSol
FSC0.143 = 4.5 Å
4 6 8
d
TMD/TaF/CTD
FSC0.143 = 4.4 Å
21.5 10.8 7.2 5.4 4.3 3.6
4.0 5.5 7.0

Supplementary Figure 10. Focused refinement of the ActMix-ET dataset.

- **a.** The FSC curve and a map colored according to the local resolution, and the mask for the N-terminal cytosolic shell part, comprising FKBP12, NTDA-B, NSol, SPRY1-3, and RY1&2 domains, performed on the symmetry-expanded particle set.
- **b.** The FSC curve and a map colored according to the local resolution, and the mask for the BSol cytosolic shell part, comprising BSol and RY3&4 domains, performed on the symmetry-expanded particle set.
- **c.** The FSC curve and a map colored according to the local resolution, and the mask for the Activation core part, comprising JSol and CSol domains, performed on the symmetry-expanded particle set.
- **d.** The FSC curve and a map colored according to the local resolution, and the mask for the TMD part, comprising TaF, S2S3, pVSD, Pore, and CTD domains, performed with an imposed C4 symmetry.

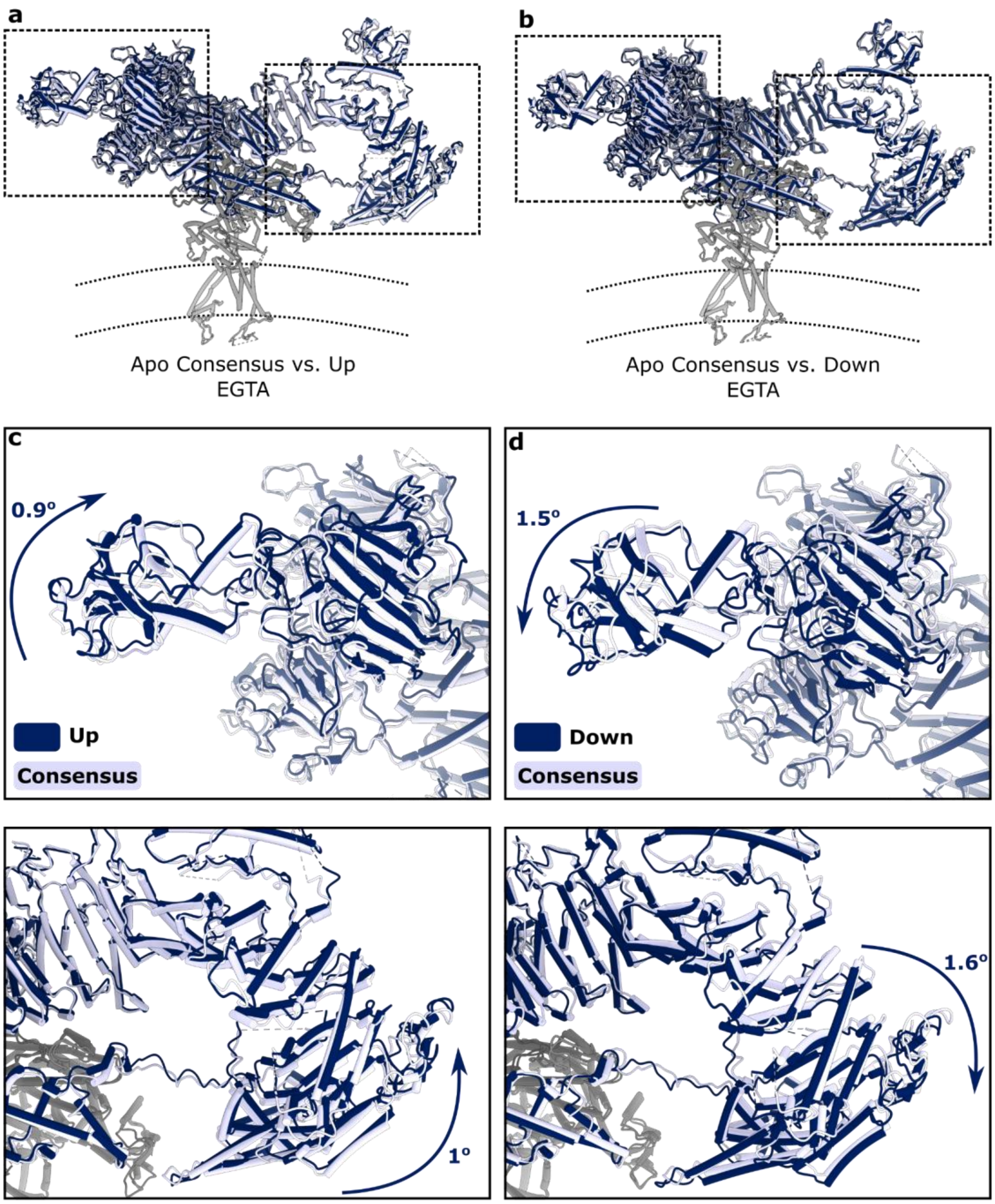

Supplementary Figure 11. Heterogeneity of the Apo-state RyR1 cytosolic shell in the native membrane, determined by StA.

- **a.** Apo-state consensus reconstruction is superimposed with the “Up” class.
- **b.** Apo-state consensus reconstruction is superimposed with the “Down” class.
- **c.** Close-up views of the two main cytosolic shell domain bundles, highlighting the differences with the “Up” class.
- **d.** Close-up views of the two main cytosolic shell domain bundles, highlighting the differences with the “Down” class.

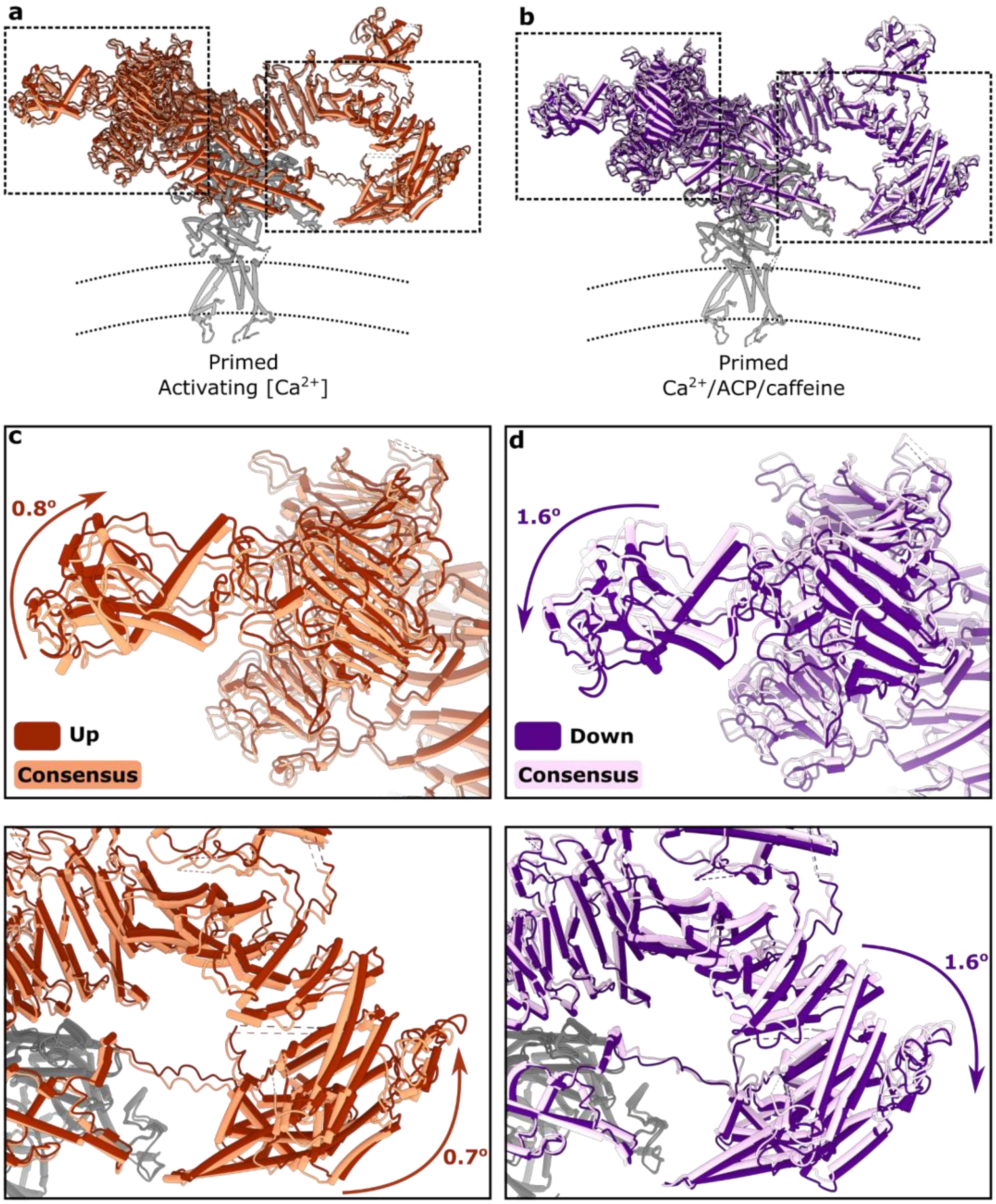

Supplementary Figure 12. Heterogeneity of the Primed-state RyR1 cytosolic shell in the native membrane, determined by SPA.

- **a.** Primed-state consensus reconstruction from the Activating [Ca^{2+}] dataset is superimposed with the "Up" class.
- **b.** Primed-state consensus reconstruction from the Activation Mix dataset is superimposed with the "Down" class.
- **c.** Close-up views of the two main cytosolic shell domain bundles, highlighting the differences with the "Up" class.
- **d.** Close-up views of the two main cytosolic shell domain bundles, highlighting the differences with the "Down" class.

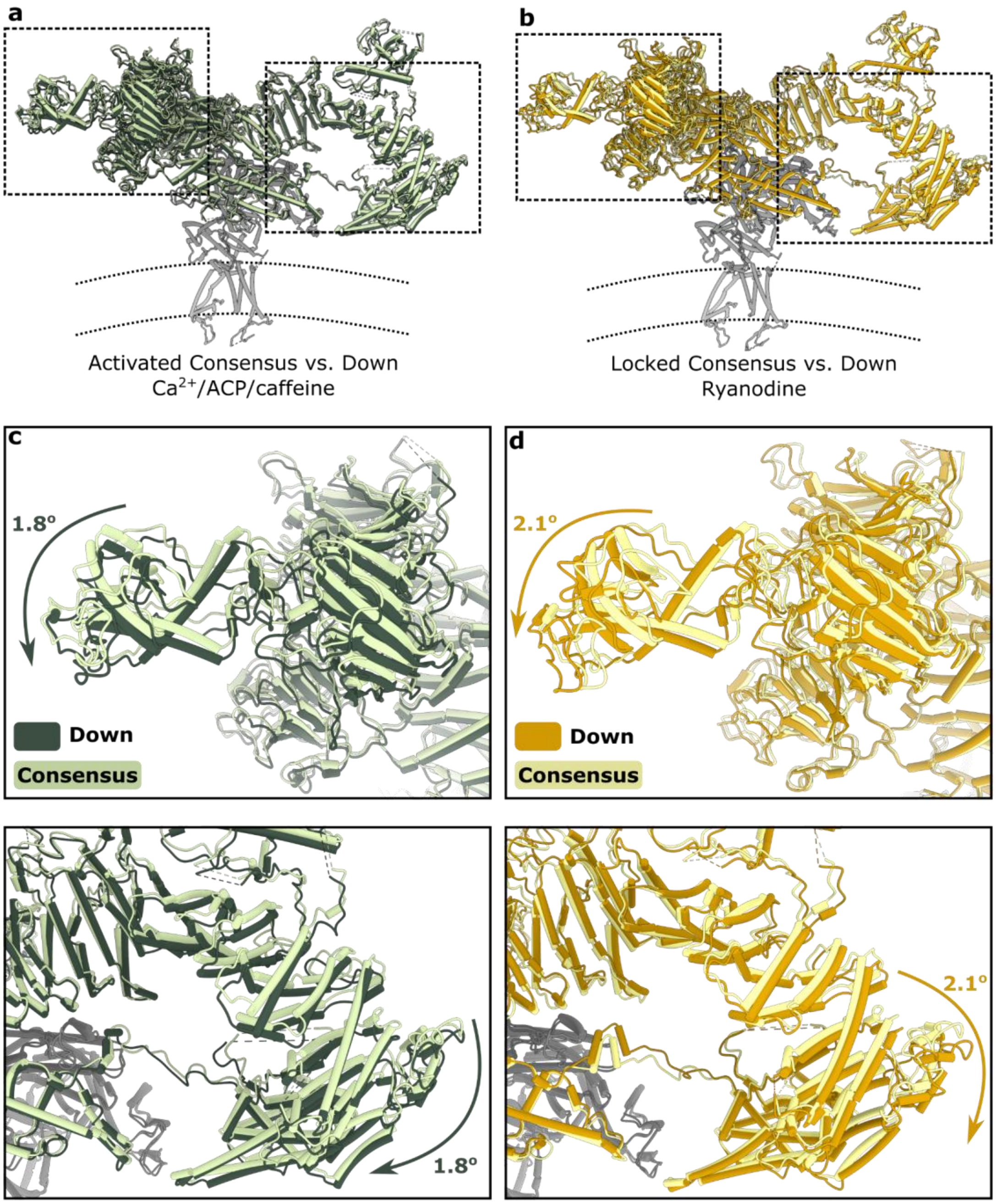

Supplementary Figure 13. Heterogeneity of the Activated and Locked-state RyR1 cytosolic shell in the native membrane, determined by SPA.

- **a.** Activated-state consensus reconstruction is superimposed with the “Down” class.
- **b.** Ryanodine Locked-state consensus reconstruction is superimposed with the “Down” class.
- **c.** Close-up views of the two main cytosolic shell domain bundles, highlighting the differences with the “Down” class.
- **d.** Close-up views of the two main cytosolic shell domain bundles, highlighting the differences with the “Down” class.

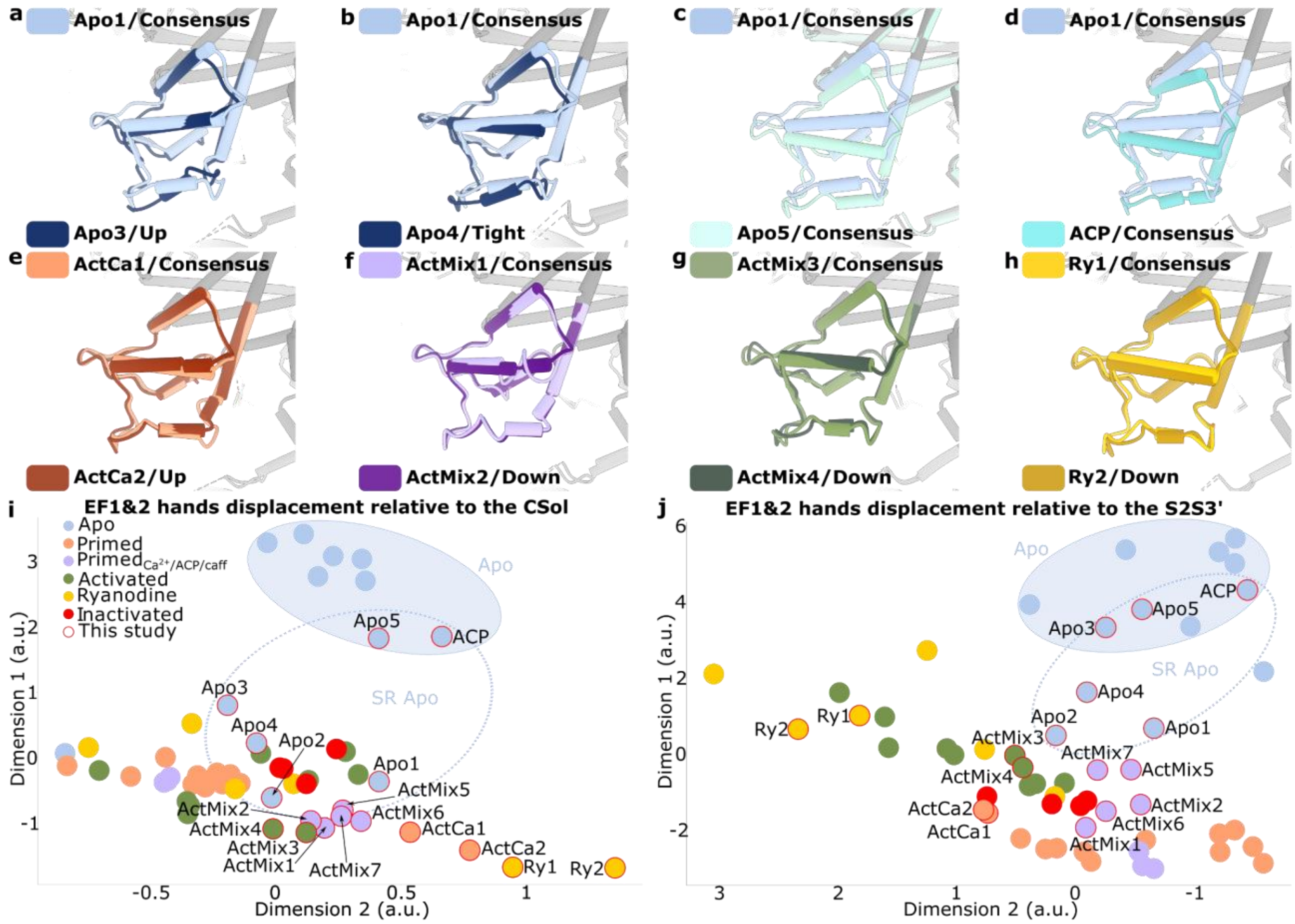

Supplementary Figure 14. Analysis of the variability of the EF1&2 hand domain in different functional states shows multiple divergent conformations of EF1&2 in the Apo state.

a. EF1&2 hands domain of the Apo-state/EGTA-ET superimposed with the “Up” class. Models are aligned on the CSol domain, which is shown in gray.

b. EF1&2 hands domain of the Apo-state/EGTA-ET superimposed with the “Tight” class.

c. EF1&2 hands domain of the Apo-state/EGTA-ET superimposed with the Apo-state/EGTA-SPA.

d. EF1&2 hands domain of the Apo-state/EGTA-ET superimposed with the Apo-state/ACP.

e. EF1&2 hands domain of the Primed-state/Activating[Ca^{2+}] superimposed with the “Up” class.

f. EF1&2 hands domain of the Primed-state/Activation Mixture Ca^{2+}/ACP/caffeine superimposed with the “Down” class.

g. EF1&2 hands domain of the Activated-state/Activating Mixture Ca^{2+}/ACP/caffeine superimposed with the “Down” class.

h. EF1&2 hands domain of the Locked-state/Ryanodine superimposed with the “Down” class.

i. Multidimensional scaling plot of the distance matrix for the EF1&2 displacement relative to the CSol. The light blue oval zone highlights the expected location of the Apo-state models on the plot. Apo1-4 models from this study cluster with other functional states instead.

j. Multidimensional scaling plot of the distance matrix for the EF1&2 displacement relative to the S2S3 helical bundle of the neighbouring protomer. The light blue oval zone highlights the

expected location of the Apo-state models on the plot. Apo1-2,4 models from this study cluster with other functional states instead.

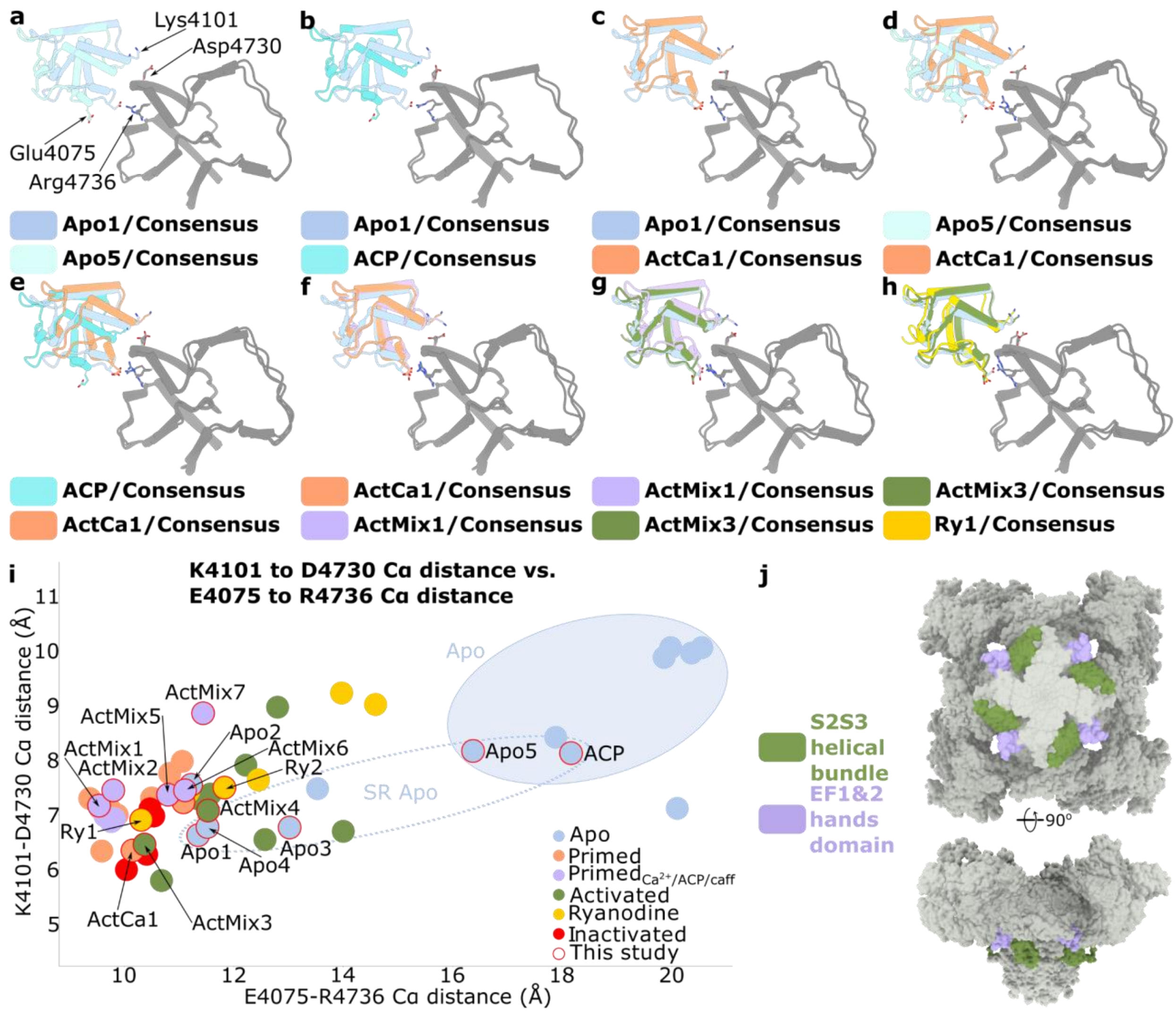

Supplementary Figure 15. Analysis of the distance between the EF1&2 hand domain and the S2S3 helical bundle of the neighbouring protomer in different functional states shows multiple divergent conformations of EF1&2 in the Apo state.

- **a.** EF1&2 hands domain of the Apo-state/EGTA-ET superimposed with the Apo-state/EGTA. Models are aligned on the S2S3 helical bundle of the neighbouring protomer, which is shown in gray. Sidechains for the K4101, D4730, E4075, and R4736 are shown in atoms.
- **b.** EF1&2 hands domain of the Apo-state/EGTA-ET superimposed with the Apo-state/ACP.
- **c.** EF1&2 hands domain of the Apo-state/EGTA-ET superimposed with the Primed-state/Activating [Ca^{2+}].
- **d.** EF1&2 hands domain of the Apo-state/EGTA superimposed with the Primed-state/Activating [Ca^{2+}].
- **e.** EF1&2 hands domain of the Apo-state/ACP superimposed with the Primed-state/Activating [Ca^{2+}].

- **f.** EF1&2 hands domain of the Primed-state/Activating [Ca^{2+}] superimposed with the Primed-state/Activating Mixture Ca^{2+}/ACP/caffeine.
- **g.** EF1&2 hands domain of the Primed-state/Activating Mixture Ca^{2+}/ACP/caffeine superimposed with the Activated-state/Activating Mixture Ca^{2+}/ACP/caffeine.
- **h.** EF1&2 hands domain of the Activated-state/Activating Mixture Ca^{2+}/ACP/caffeine, superimposed with the Locked-state/Ryanodine.
- **i.** K4101 to D4730 Calpha distance vs. E4075 to R4736 Calpha distance plot for the publicly available RyR1 models, as well as the models from this study. The light blue oval zone highlights the expected location of the Apo-state models on the plot. Apo1-4 models from this study cluster with other functional states instead.
- **j.** Localisation of the EF1&2 domain and S2S3 helical bundle is shown on the tetramer from the bottom (lumenal) and side views.

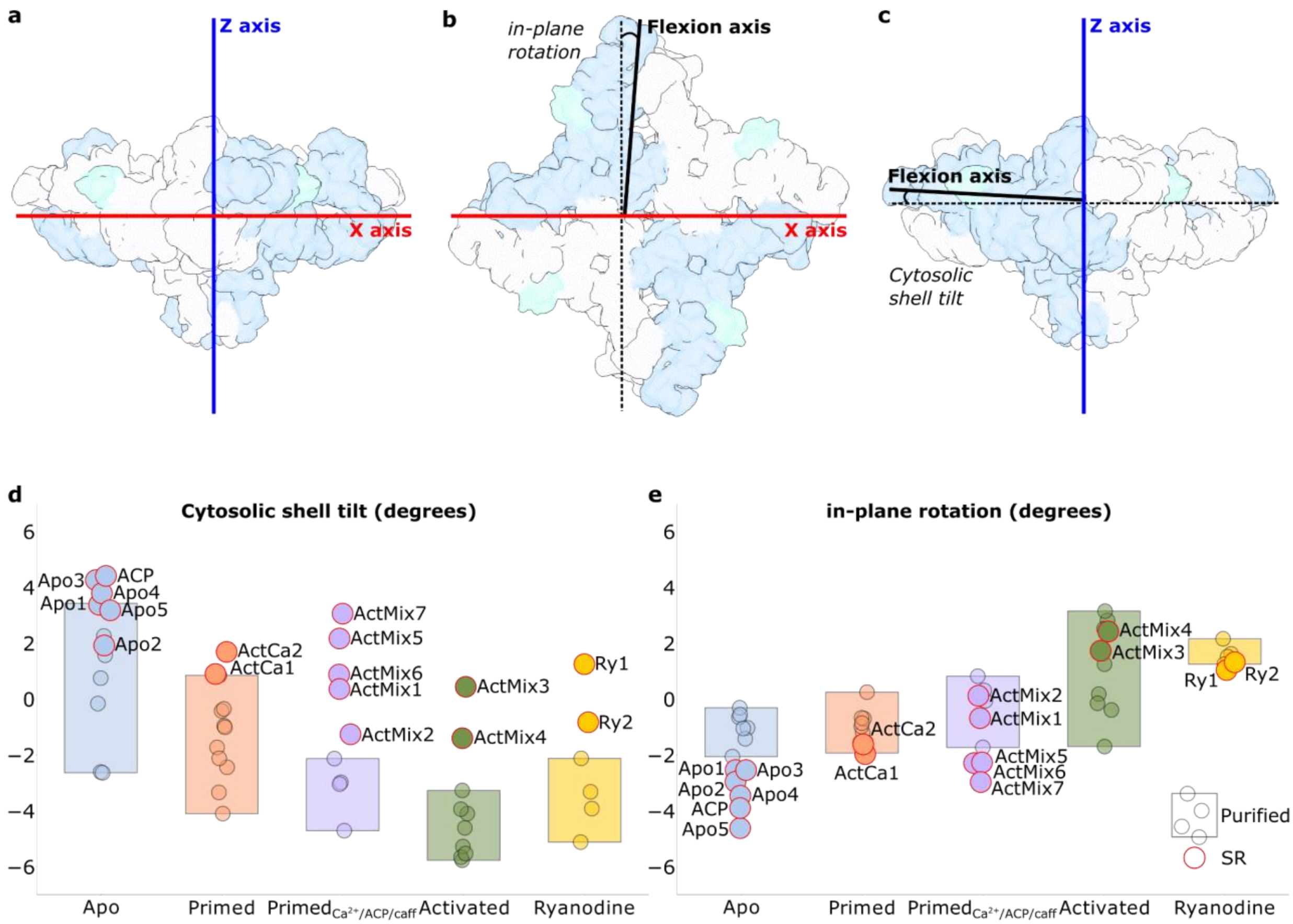

Supplementary Figure 16. Quantification of cytosolic shell tilt and in-plane rotation measurements.

- **a.** The schematic of the X and Z axes, as it was defined for the angle measurement, is shown from the channel side.
- **b.** The schematic of the Flexion axis, as it was defined for the in-plane rotation measurement against the X axis, is shown from the channel top.
- **c.** The schematic of the same Flexion axis, as it was defined for the cytosolic shell tilt measurement against the Z axis, is shown from the channel top.
- **d.** Cytosolic shell tilt angle ranges are plotted from the publicly available models, with SR models from this study scattered on top.
- **e.** In-plane rotation angle ranges are plotted from the publicly available modes, with SR models from this study scattered on top.

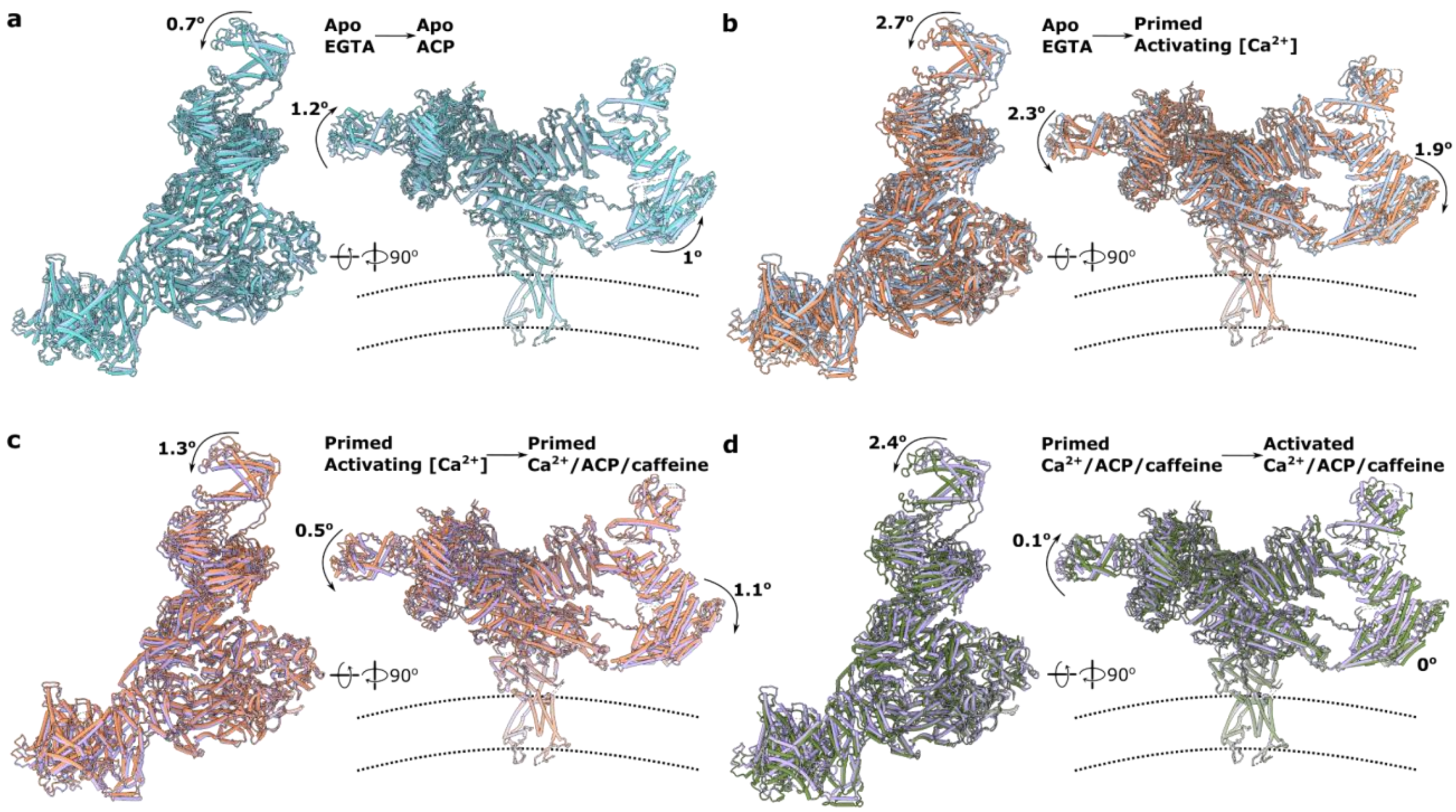

Supplementary Figure 17. Quantification of conformational changes of the cytosolic shell domains upon activation.

- **a.** Apo-state/EGTA and Apo-state/ACP superimposed with the cytoplasmic shell rotation and tilt marked with arrows on the top and side views, respectively. Only one protomer is shown for clarity; membrane location is shown with dashed lines.
- **b.** Apo-state/EGTA and Primed-state/Activating [Ca^{2+}] superimposed.
- **c.** Primed-state/Activating [Ca^{2+}] and Primed-state/Activating Mixture Ca^{2+}/ACP/caffeine superimposed.
- **d.** Primed-state/Activating Mixture Ca^{2+}/ACP/caffeine and Activated-state/Activating Mixture Ca^{2+}/ACP/caffeine superimposed.

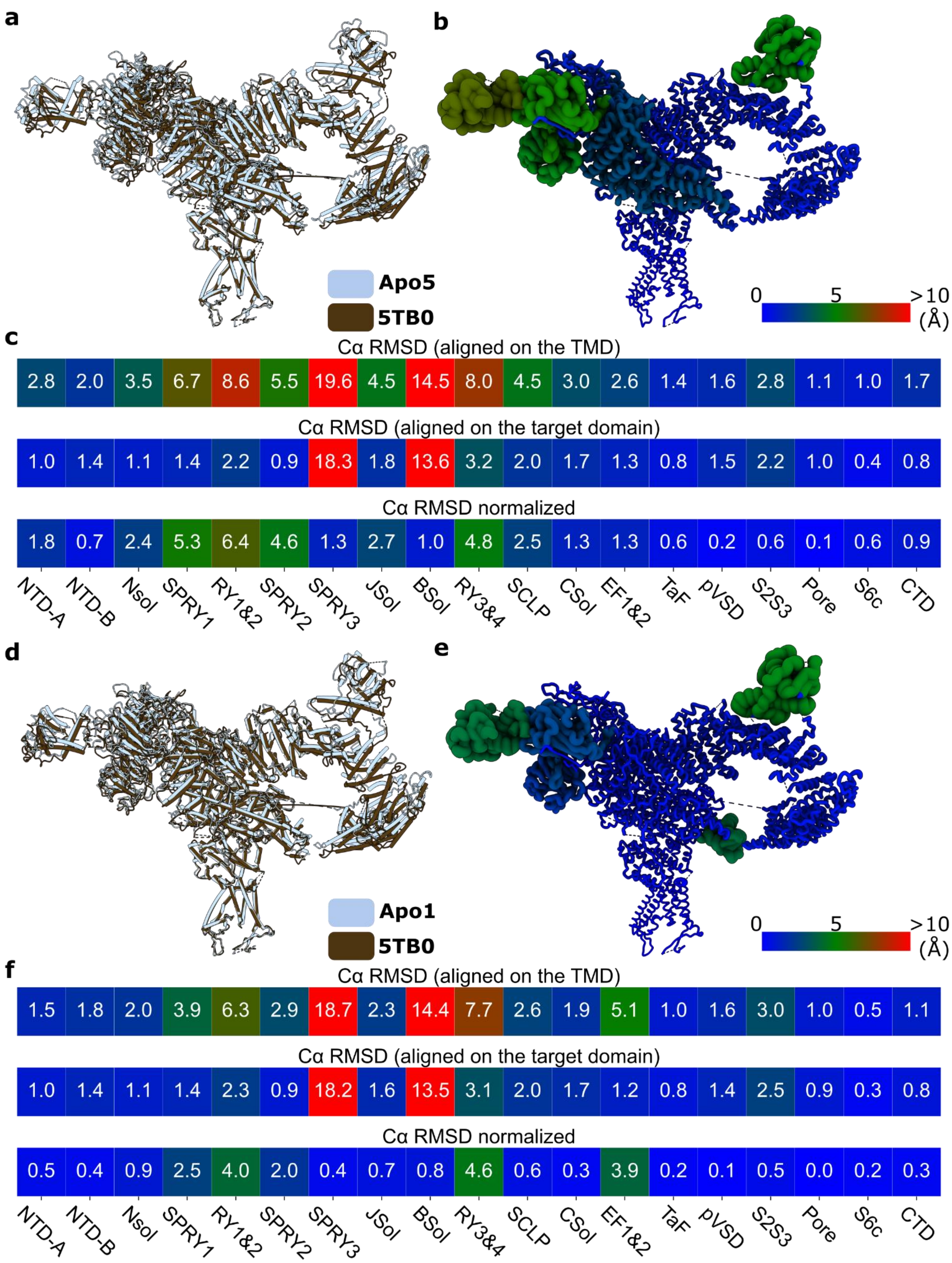

Supplementary Figure 18. Comparison of the Apo-state SR structures with the structures of purified receptors.

- **a.** The consensus model from the EGTA dataset (Apo5) superimposed with the 5TB0.
- **b.** The consensus model from the EGTA dataset is colored according to the per-domain RMSD from the 5TB0, and the cartoon representation is displayed with varying thickness depending on the RMSD value.
- **c.** Table of the per-domain RMSD values. Colored in the same range as **b**. The normalized RMSD is achieved by subtracting the RMSD of the pre-aligned target domain from the RMSD of the target domain while aligned on the TMD.
- **d.** The consensus model from the EGTA-ET dataset (Apo1) superimposed with the 5TB0.
- **e.** The consensus model from the EGTA-ET dataset is colored according to the per-domain RMSD from the 5TB0, and the cartoon representation is displayed with varying thickness depending on the RMSD value.
- **f.** Table of the per-domain RMSD values. Colored in the same range as **e**.

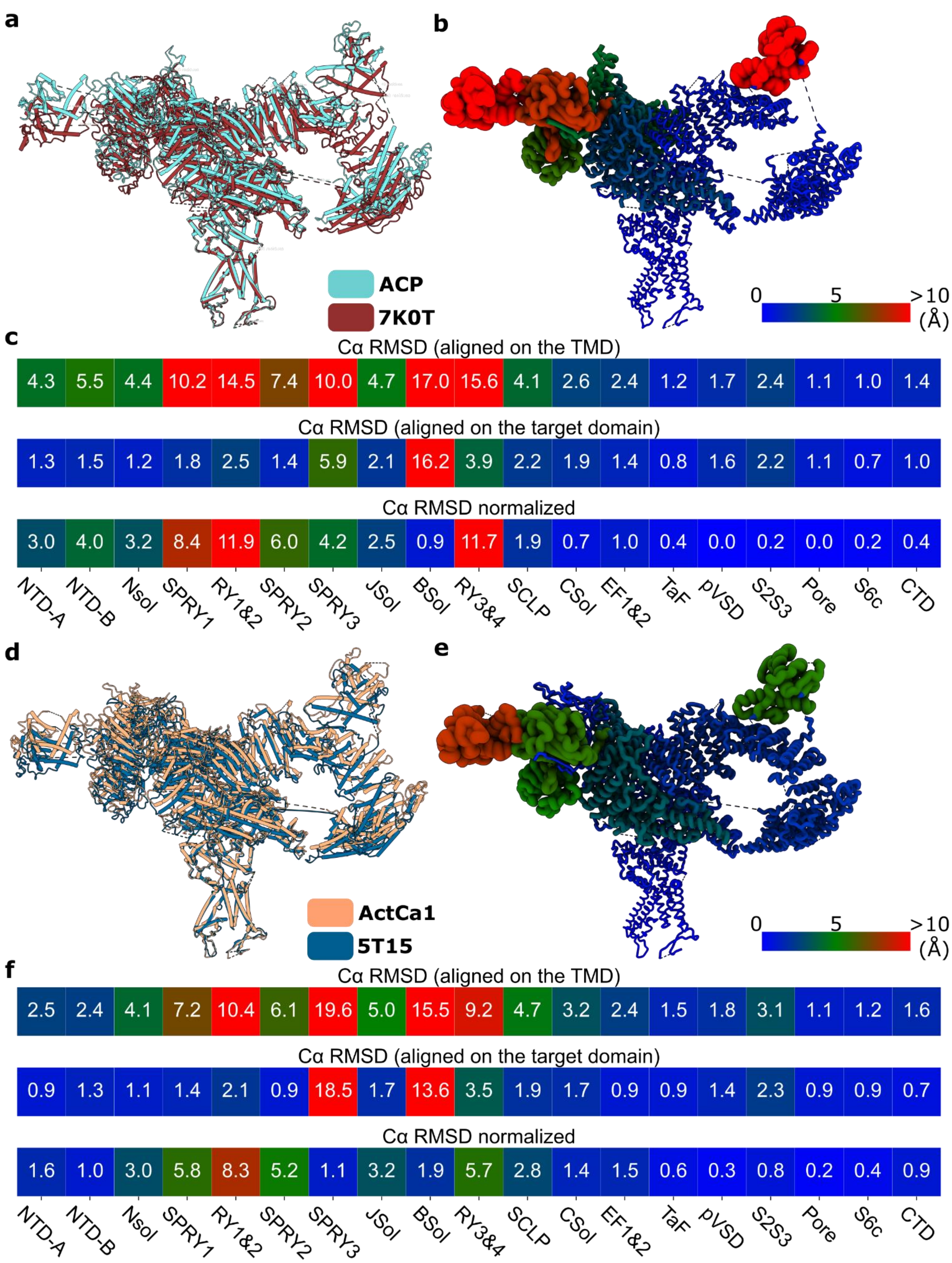

Supplementary Figure 19. Comparison of the Apo-state (ACP) and Primed-state (ActCa) SR structures with the structures of purified receptors.

- **a.** The consensus model from the ACP dataset superimposed with the 7K0T.
- **b.** The consensus model from the ACP dataset is colored according to the per-domain RMSD from the 7K0T, and the cartoon representation is displayed with varying thickness depending on the RMSD value.
- **c.** Table of the per-domain RMSD values. Colored in the same range as **b**. The normalized RMSD is achieved by subtracting the RMSD of the pre-aligned target domain from the RMSD of the target domain while aligned on the TMD.
- **d.** The consensus model from the ActCa dataset superimposed with the 5T15.
- **e.** The consensus model from the ActCa dataset is colored according to the per-domain RMSD from the 5T15, and the cartoon representation is displayed with varying thickness depending on the RMSD value.
- **f.** Table of the per-domain RMSD values. Colored in the same range as **e**.

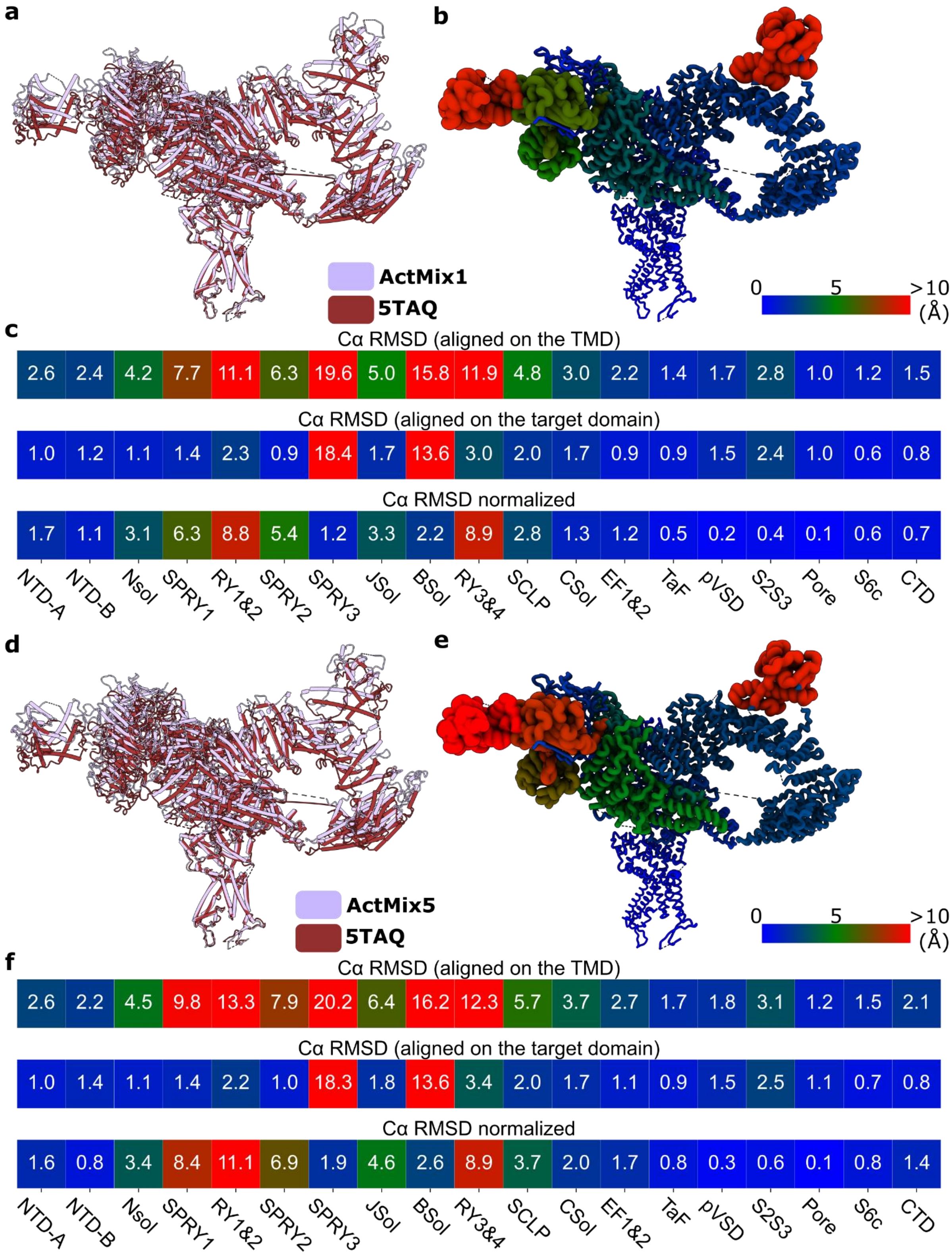

Supplementary Figure 20. Comparison of the Primed-state SR structures with the structures of purified receptors.

- **a.** The consensus model from the ActMix dataset superimposed with the 5TAQ.
- **b.** The consensus model from the ActMix dataset is colored according to the per-domain RMSD from the 5TAQ, and the cartoon representation is displayed with varying thickness depending on the RMSD value.
- **c.** Table of the per-domain RMSD values. Colored in the same range as **b**. The normalized RMSD is achieved by subtracting the RMSD of the pre-aligned target domain from the RMSD of the target domain while aligned on the TMD.
- **d.** The consensus model from the ActMix-ET dataset superimposed with the 5TAQ.
- **e.** The consensus model from the ActMix-ET dataset is colored according to the per-domain RMSD from the 5TAQ, and the cartoon representation is displayed with varying thickness depending on the RMSD value.
- **f.** Table of the per-domain RMSD values. Colored in the same range as **e**.

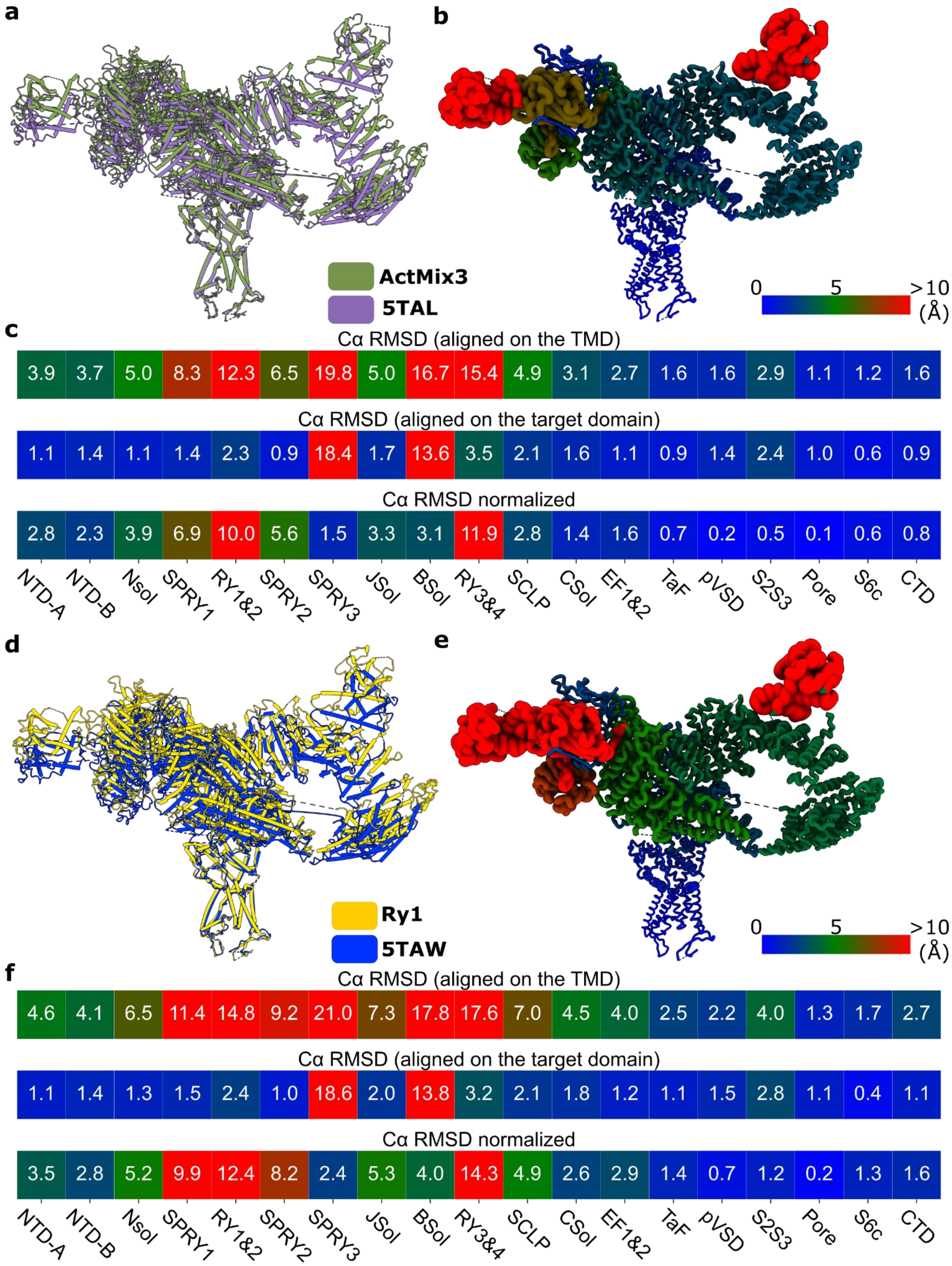

Supplementary Figure 21. Comparison of the Activated and Locked-state SR structures with the structures of purified receptors.

- **a.** The consensus model from the ActMix dataset superimposed with the 5TAL.
- **b.** The consensus model from the ActMix dataset is colored according to the per-domain RMSD from the 5TAL, and the cartoon representation is displayed with varying thickness depending on the RMSD value.
- **c.** Table of the per-domain RMSD values. Colored in the same range as **b**. The normalized RMSD is achieved by subtracting the RMSD of the pre-aligned target domain from the RMSD of the target domain while aligned on the TMD.
- **d.** The consensus model from the Ryanodine dataset superimposed with the 5TAW.
- **e.** The consensus model from the Ryanodine dataset is colored according to the per-domain RMSD from the 5TAW, and the cartoon representation is displayed with varying thickness depending on the RMSD value.
- **f.** Table of the per-domain RMSD values. Colored in the same range as **e**.

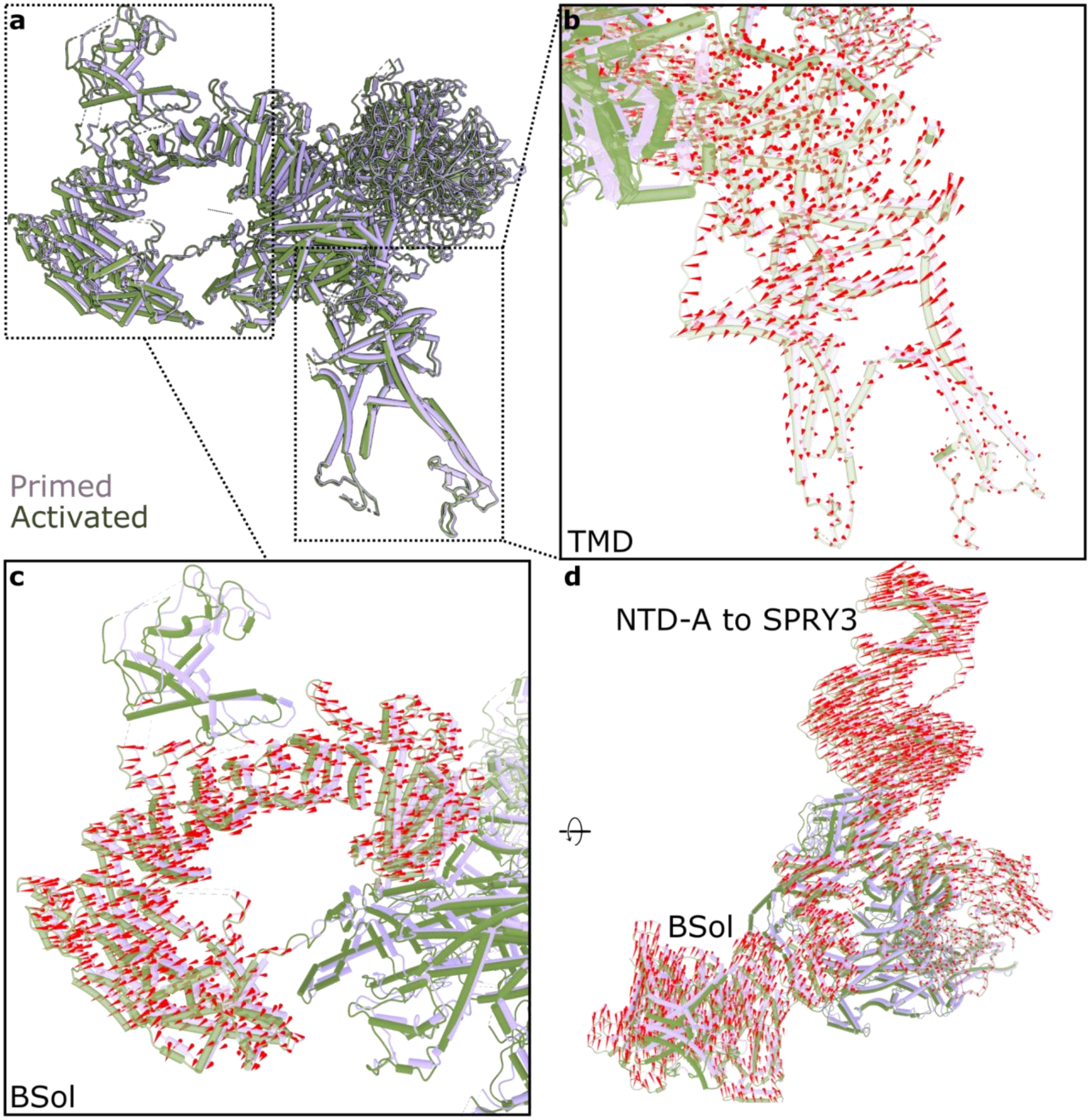

Supplementary Figure 22. Detailed view of the conformational change between the Primed and Activated states from the ActMix.

- **a.** Gating transitions are shown on the protomer.
- **b.** As the S6 helices dilate and the domains in the TMD and activation core are rearranged similarly as for the purified receptor.
- **c.** BSol goes through a plastic deformation and changes its inner conformation rather than tilting down.
- **d.** The in-plane rotation of the cytosolic shell is shown from the top.

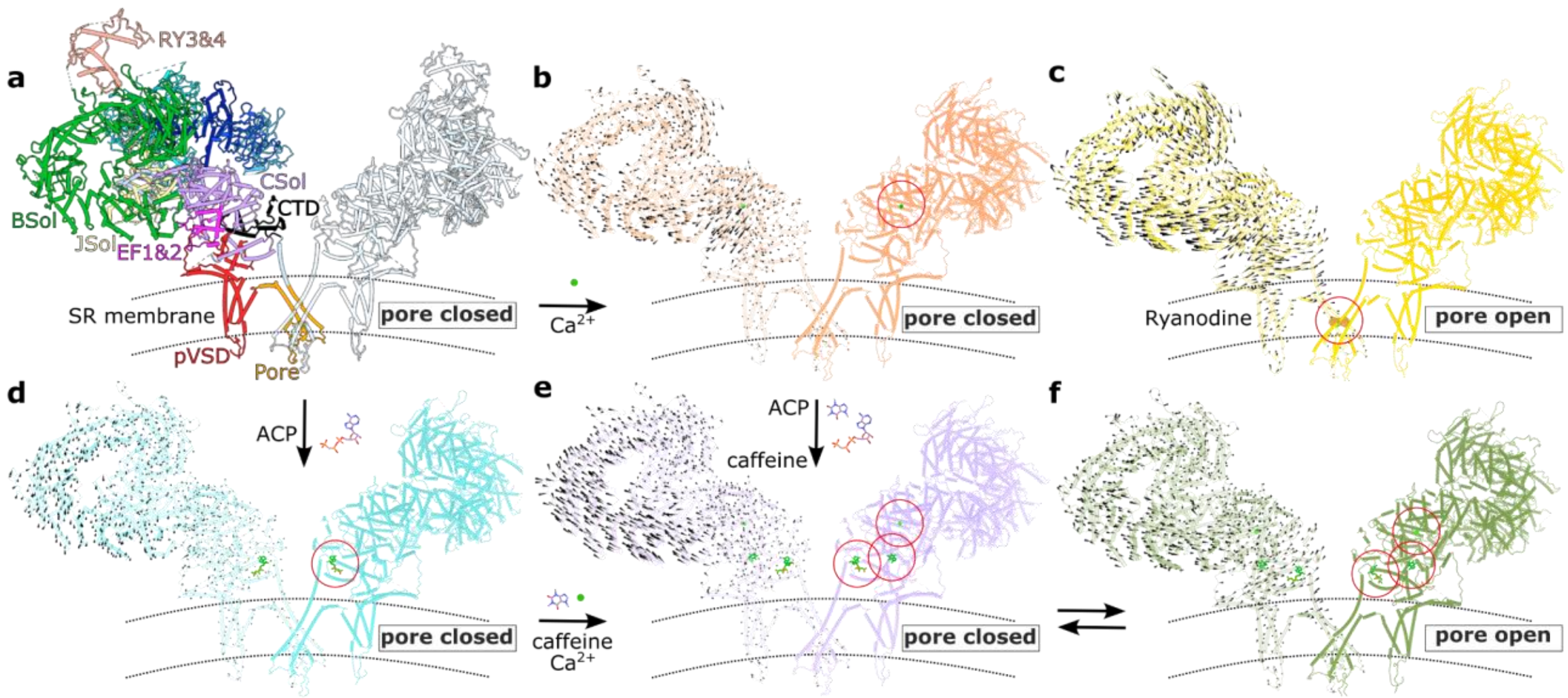

Supplementary Figure 23. Models of RyR1 in multiple functional states (Apo to Activated) reveal that activation of RyR1 is associated with large rotations of the cytosolic shell domains, as shown from the side.

- **a.** Apo-state model from the EGTA dataset with the domains labeled, shown from the side view.
- **b.** Apo-state model from the ACP dataset with vectors on one of the protomers showing the conformational change between the EGTA Apo-state and the Apo-state upon the addition of ACP, shown from the side view.
- **c.** Locked-state model from the Ryanodine dataset with vectors on one of the protomers showing the conformational change between the EGTA-ET Apo-state and the Locked-state upon the addition of Ryanodine, shown from the side view.
- **d.** Primed-state model from the Activating [Ca^{2+}] dataset with vectors on one of the protomers showing the conformational change between the EGTA-ET Apo-state and the Primed-state upon the addition of Activating [Ca^{2+}], shown from the side view.
- **e.** Primed-state model from the Ca^{2+}/ACP/caffeine dataset with vectors on one of the protomers showing the conformational change between the Activating [Ca^{2+}] Primed-state and the Primed-state upon the addition of ACP and caffeine, shown from the side view.
- **f.** Activated-state model from the Ca^{2+}/ACP/caffeine dataset with vectors on one of the protomers showing the conformational change between the Ca^{2+}/ACP/caffeine Primed-state and the Activated-state upon the opening of the pore, shown from the side view.

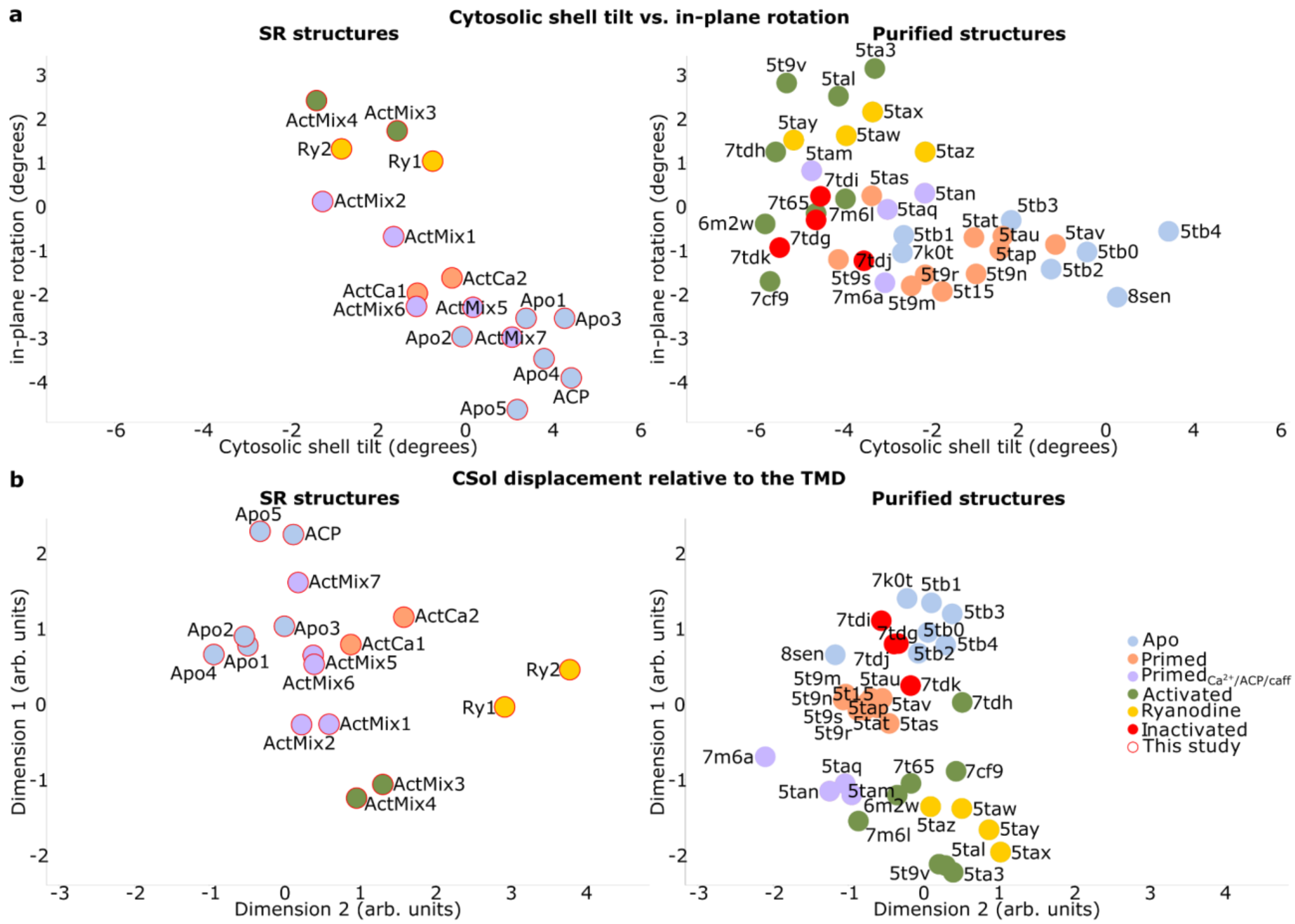

Supplementary Figure 24. Cytosolic shell tilt vs in-plane rotation and CSol displacement scatter plots with the previously reported models labeled.

- **a.** Cytosolic shell tilt against in-plane rotation plot for the publicly available RyR1 models as well as the models from this study. Models are colored by functional state, and the models from the current study are circled in red. SR structures (this study) are shown on the left, and the purified structures (previous reports) are shown on the right.
- **b.** Multidimensional scaling plot of the distance matrix for the CSol displacement relative to the TMD.

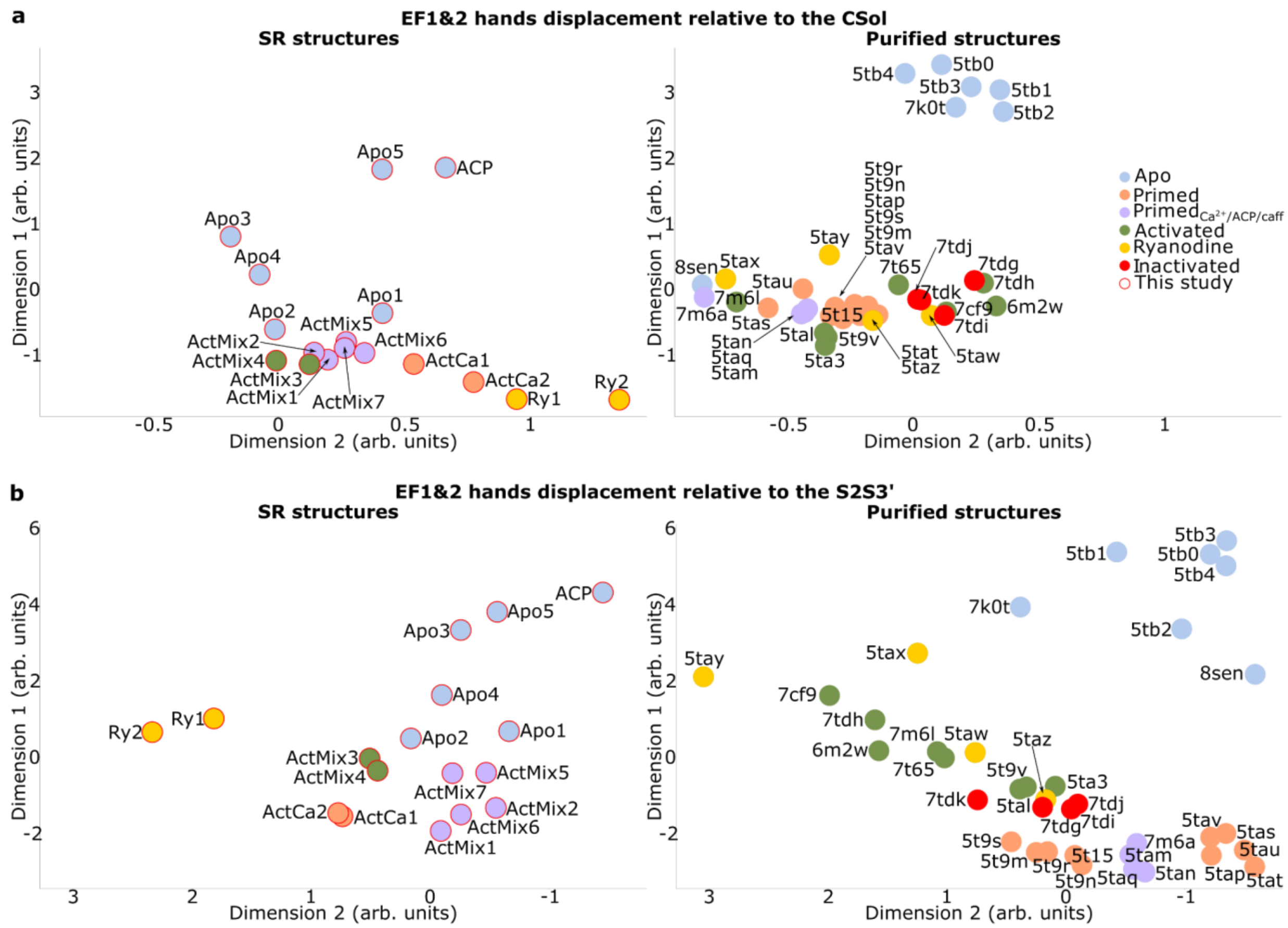

Supplementary Figure 25. EF1&2 displacement relative to the CSol and the S2S3' helical bundle of the neighboring protomer scatter plots with the previously reported models labeled.

a. Multidimensional scaling plot of the distance matrix for the EF1&2 displacement relative to the CSol. Models are colored by functional state, and the models from the current study are circled in red. SR structures (this study) are shown on the left, and the purified structures (previous reports) are shown on the right.

b. Multidimensional scaling plot of the distance matrix for the EF1&2 displacement relative to the S2S3' helical bundle of the neighboring protomer.

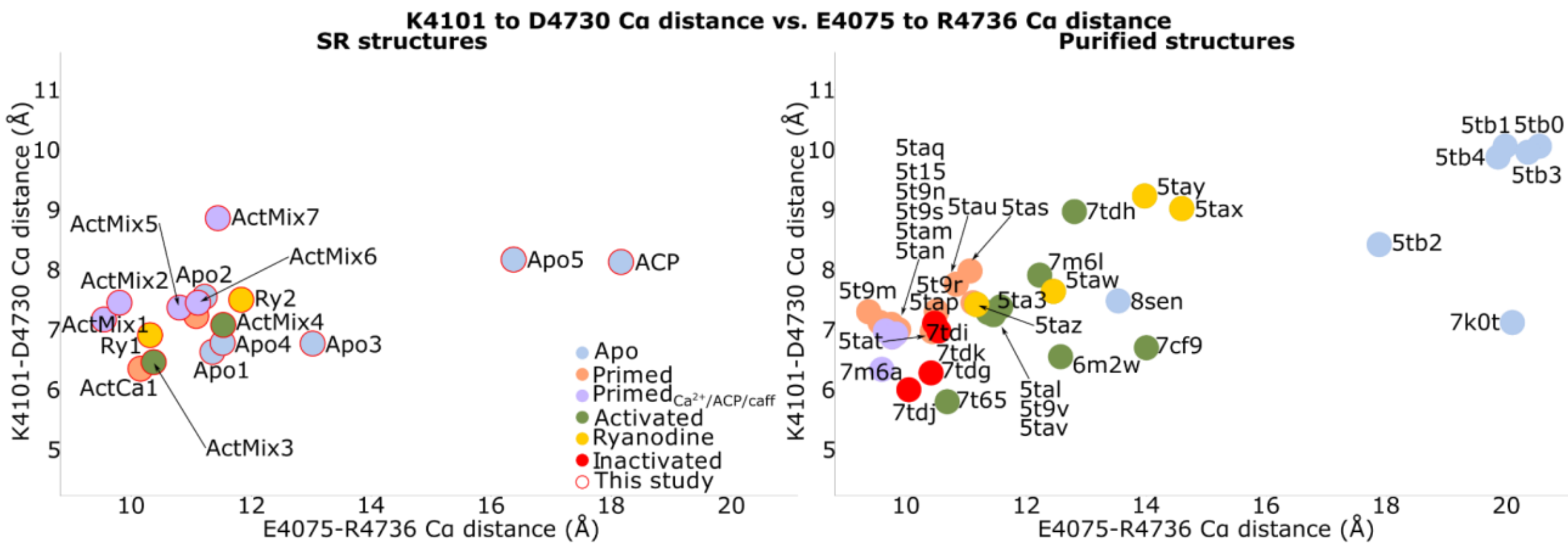

Supplementary Figure 26. K4101 to D4730 Calpha distance vs. E4075 to R4736 Calpha distance plot with the previously reported models labeled.

K4101 to D4730 C-alpha distance vs. E4075 to R4736 C-alpha distance plot for the publicly available RyR1 models, as well as the models from this study. Models are colored by functional state, and the models from the current study are circled in red. SR structures (this study) are shown on the left, and the purified structures (previous reports) are shown on the right.

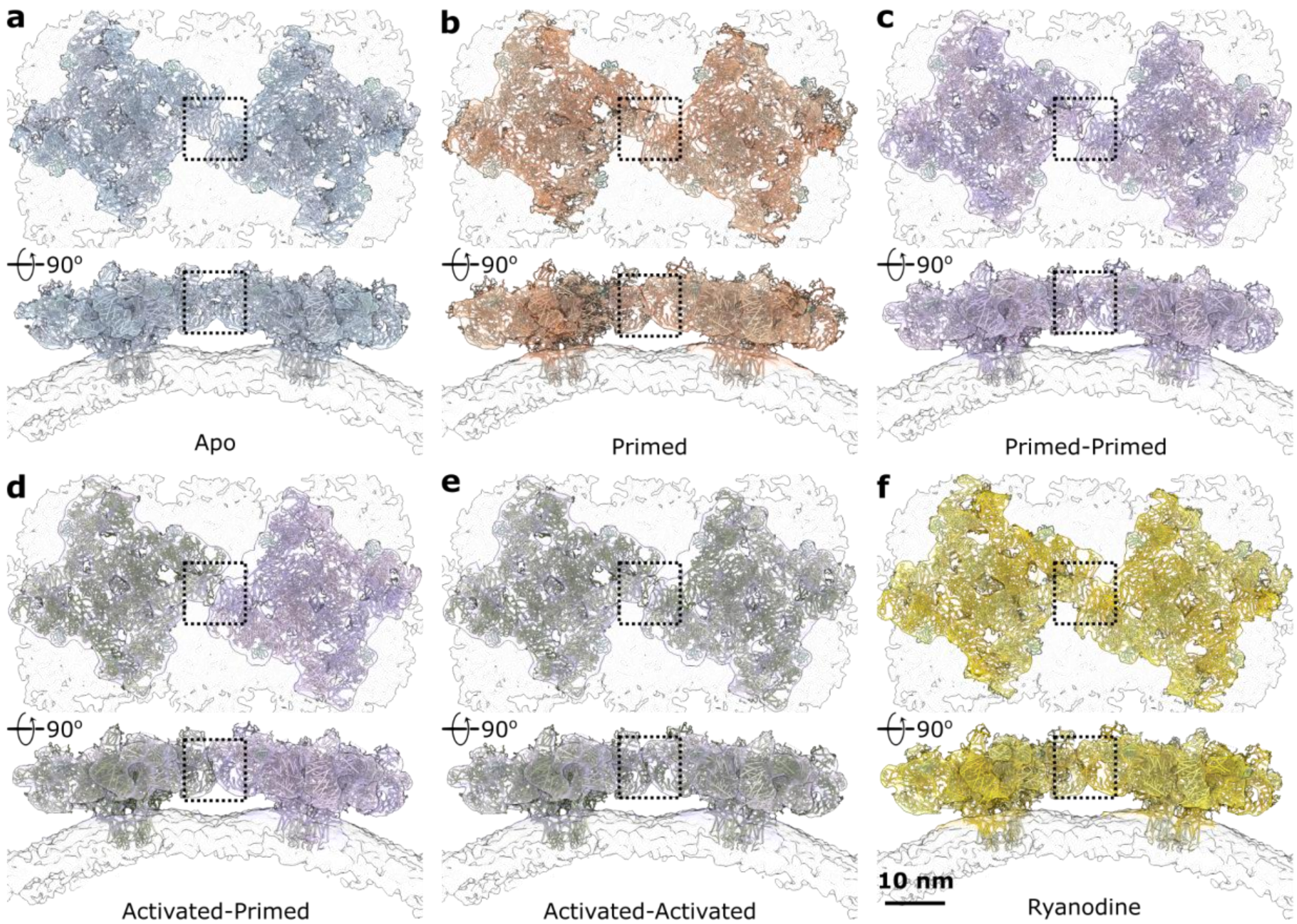

Supplementary Figure 27. RyR1-RyR1 dimer maps in native membranes in the Apo, Primed, Activated, and Ryanodine-Locked states.

- **a.** Apo-state dimer map from the EGTA-ET dataset, top and side views with the corresponding atomic models rigid-body fitted into the density.
- **b.** Primed-state dimer map from the Activating [Ca^{2+}] dataset, top and side views with the corresponding atomic models rigid-body fitted into the density.
- **c.** Dimer map from the Ca^{2+}/ACP/caffeine dataset, top and side views with the Primed and Primed-state atomic models rigid-body fitted into the density.
- **d.** Dimer map from the Ca^{2+}/ACP/caffeine dataset, top and side views with the Activated and Primed-state atomic models rigid-body fitted into the density.
- **e.** Dimer map from the Ca^{2+}/ACP/caffeine dataset, top and side views with the Activated and Activated-state atomic models rigid-body fitted into the density.
- **f.** Locked-state dimer map from the Ryanodine dataset, top and side views with the corresponding atomic models rigid-body fitted into the density.

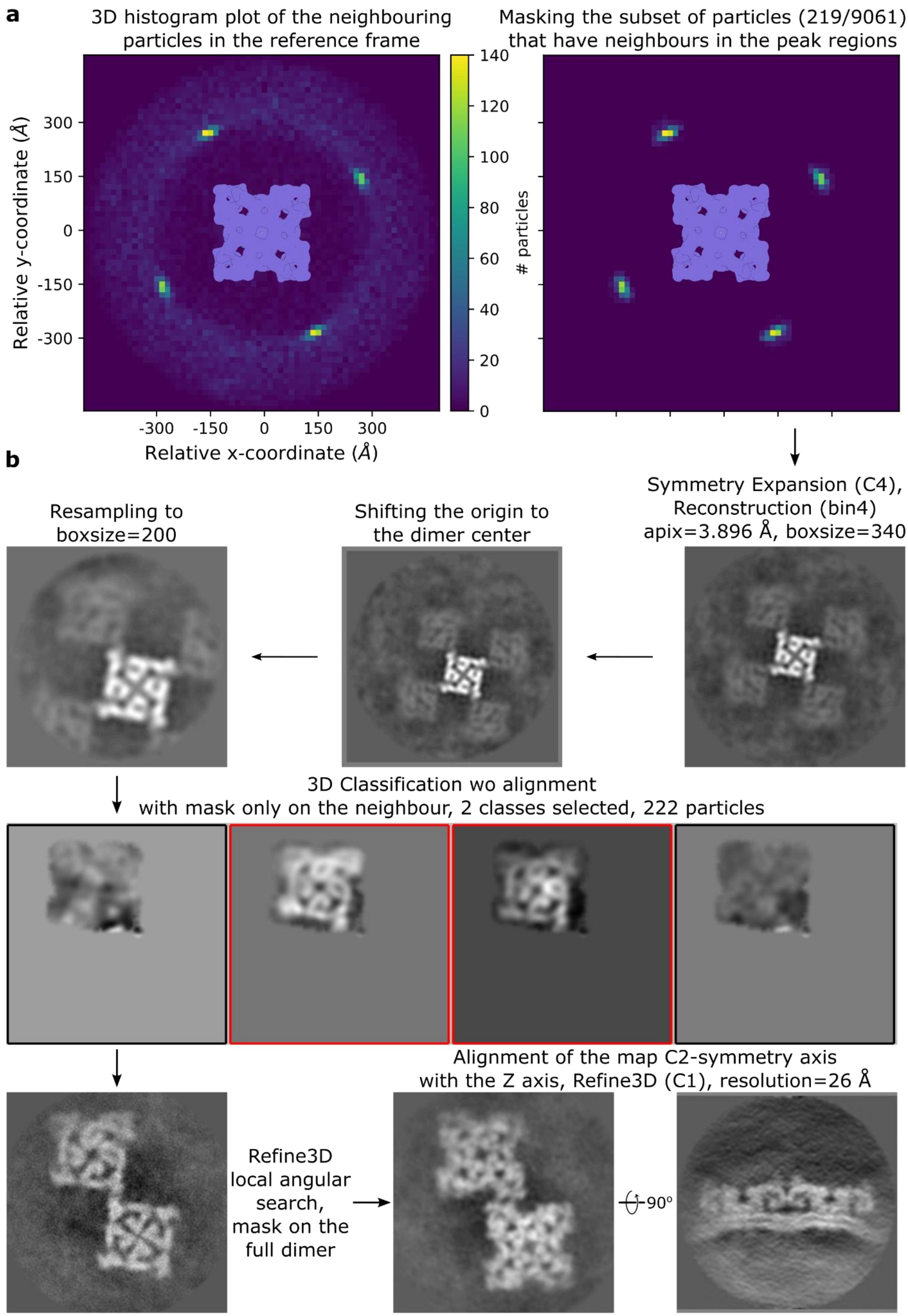
a
3D histogram plot of the neighbouring particles in the reference frame
Masking the subset of particles (219/9061) that have neighbours in the peak regions
Relative y-coordinate (Å)
Relative x-coordinate (Å)
300
150
0
-150
-300
particles
140
120
100
80
60
40
20
b
Symmetry Expansion (C4), Reconstruction (bin4) apix=3.896 Å, boxsize=340
Shifting the origin to the dimer center
Resampling to boxsize=200
3D Classification wo alignment with mask only on the neighbour, 2 classes selected, 222 particles
Refine3D local angular search, mask on the full dimer
Alignment of the map C2-symmetry axis with the Z axis, Refine3D (C1), resolution=26 Å
90º

Supplementary Figure 28. A workflow for structural determination of the RyR1-RyR1 dimer map for the EGTA-ET dataset.

- **a.** 3D histogram plot of the neighboring particles in the reference frame; a sum along the Z direction is shown. The four distinct C4-symmetry-related peaks correspond to the RyR1-RyR1 dimer interaction. The 3D histogram plot from a is masked, and only particles with neighbors in one of the four allowed regions are included.
- **b.** The StA workflow for obtaining the final dimer reconstruction from the isolated-particle subset. The initial box size was 132 nm for the bin4 volumes.

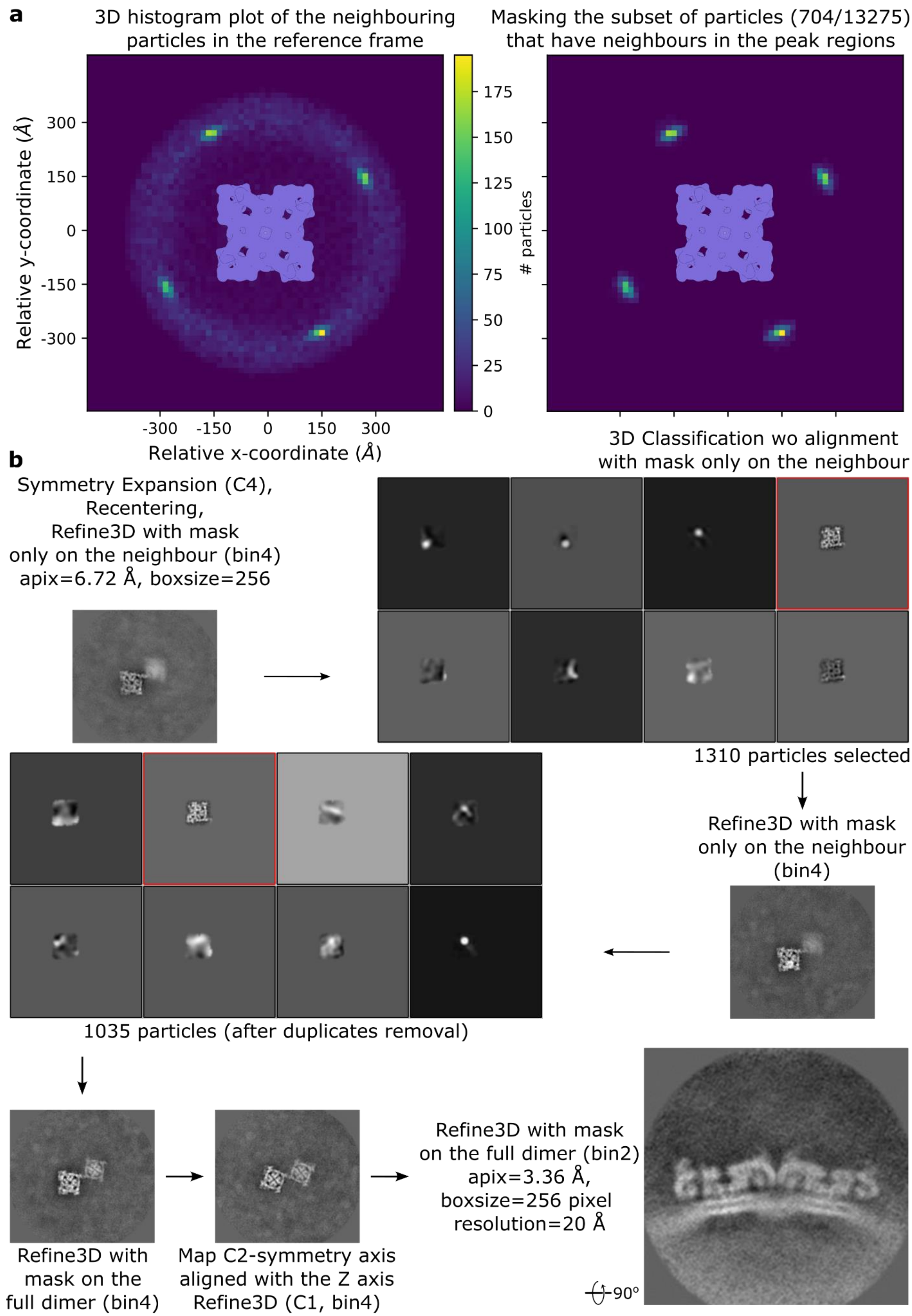
a
3D histogram plot of the neighbouring particles in the reference frame
Masking the subset of particles (704/13275) that have neighbours in the peak regions
Relative y-coordinate (Å)
300
150
0
-150
-300
-300
-150
0
150
300
Relative x-coordinate (Å)
175
150
125
100
75
50
25
0
particles
b
Symmetry Expansion (C4), Recentering, Refine3D with mask only on the neighbour (bin4) apix=6.72 Å, boxsize=256
3D Classification wo alignment with mask only on the neighbour
1310 particles selected
Refine3D with mask only on the neighbour (bin4)
1035 particles (after duplicates removal)
Refine3D with mask on the full dimer (bin4)
Map C2-symmetry axis aligned with the Z axis Refine3D (C1, bin4)
Refine3D with mask on the full dimer (bin2) apix=3.36 Å, boxsize=256 pixel resolution=20 Å
-90°

Supplementary Figure 29. Structural determination workflow for the RyR1-RyR1 dimer map for the ActMix-ET dataset.

- **a.** 3D histogram plot of the neighboring particles in the reference frame; a sum along the Z direction is shown. The four distinct C4-symmetry-related peaks correspond to the RyR1-RyR1 dimer interaction. The 3D histogram plot from a is masked, and only particles with neighbors in one of the four allowed regions are included.
- **b.** The StA workflow for obtaining the final dimer reconstruction from the isolated-particle subset. The initial box size was 172 nm for the bin4 volumes.

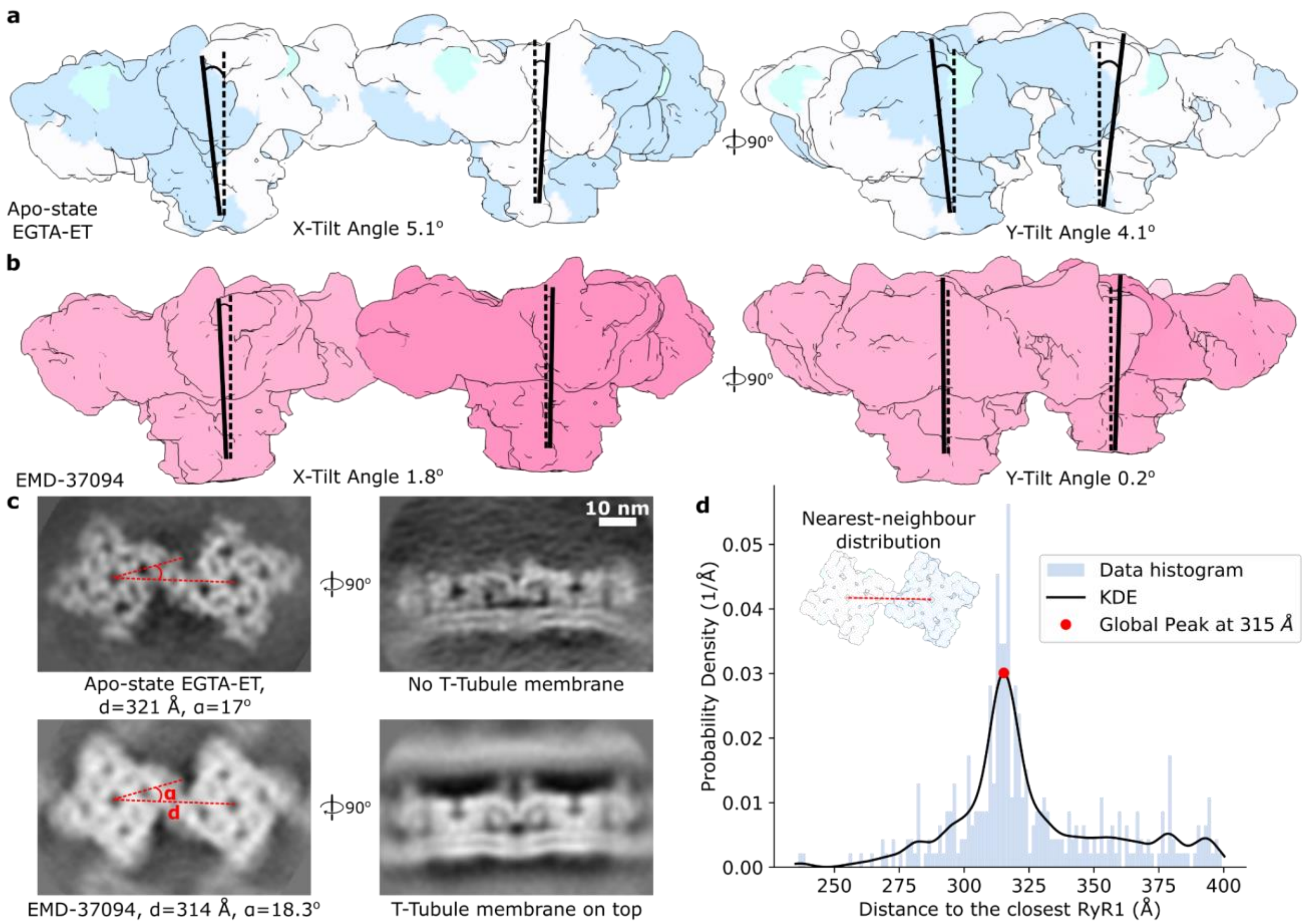

Supplementary Figure 30. Comparison of the Apo-state dimer structures from the isolated SR vesicles (this study) and the *in situ* structure from the FIB-milled triad junctions.

- **a.** SR dimer shown from X and Y side views with out-of-plane tilt angles measured in the X and Y directions.
- **b.** *In situ* dimer shown from X and Y side views with out-of-plane tilt angles measured in the X and Y directions.
- **c.** SR and in situ dimers are shown as map central slices (sum of 10) from top and side views. The distance between the channel centers and the angle between the center-to-center and the diagonal lines are measured for both structures.
- **d.** The normalized histogram (probability density) of distances between two neighboring RyR1s in the EGTA-ET dataset (n=668 particles). A peak value of 31.5 nm was found by fitting a Gaussian kernel density estimate (KDE) and taking the global maximum of the KDE curve.

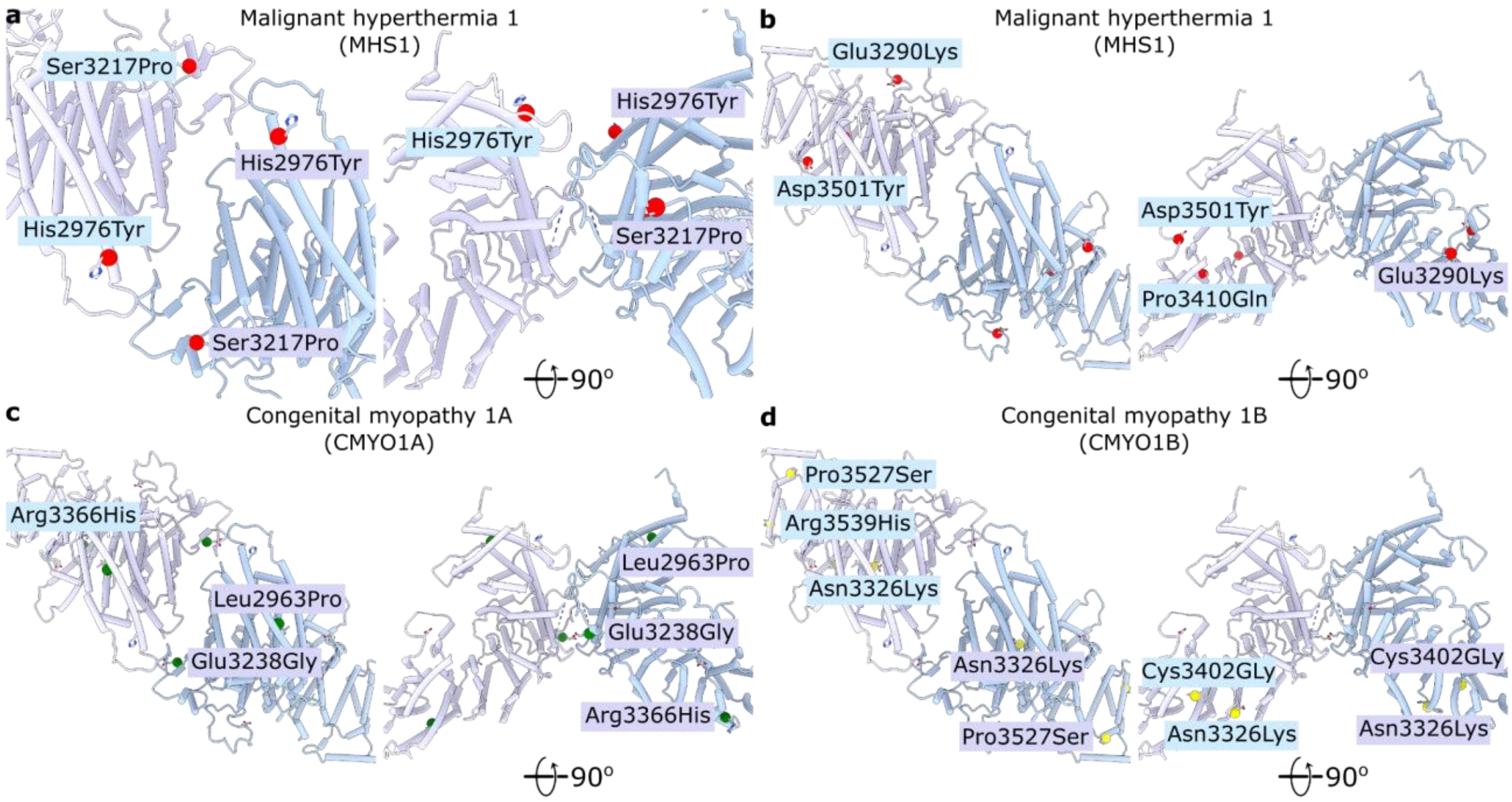

Supplementary Figure 31. Human disease-linked point mutation hotspots mapped on the RyR1-RyR1 corner-to-corner interface. Malignant hyperthermia 1 (MHS1)-related hotspots are marked with red spheres, Congenital myopathy 1A (CMYO1A) with green spheres, and Congenital myopathy 1B (CMYO1B) with yellow spheres.

a. HIS2976 and SER3217 residues close to the channel interface.

b. GLU3290, PRO3410, and ASP3501 residues in the post-RY3&4 BSol part in the receptor interface vicinity.

c. LEU2963, GLU3238, and ARG3366 residues close to the RyR1-RyR1 corner-to-corner interface.

d. ASN3326, CYS3402, PRO3527, and ARG3539 residues in the post-RY3&4 BSol part in the channels interface vicinity.

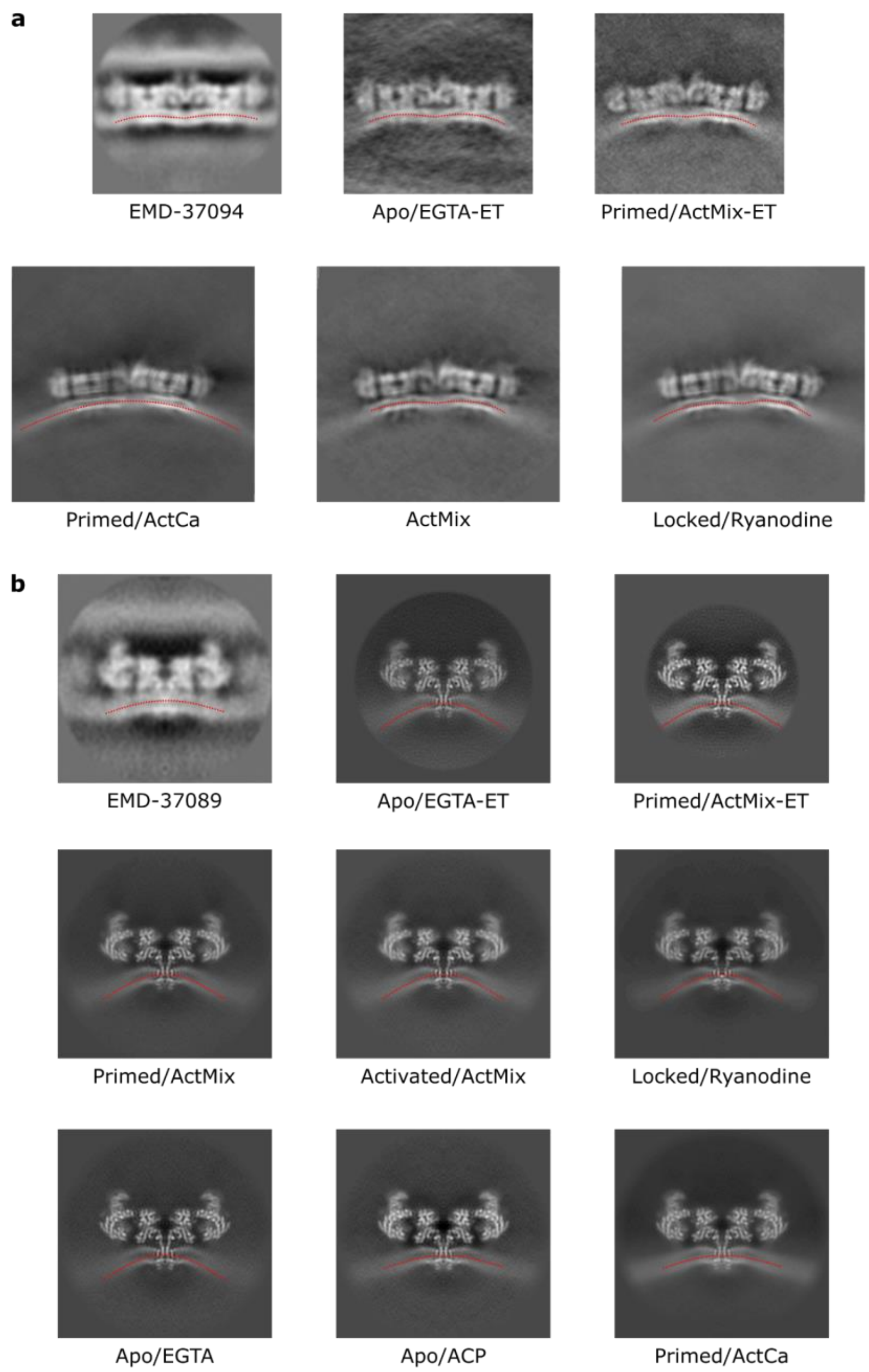

Supplementary Figure 32. SR membrane curvatures in the dimer and monomer structures.

- **a.** Consensus dimer maps are shown as central slices (sum of 10) viewed from the side; the *in situ* dimer map is also shown for comparison. Membrane curvature is depicted as a red dashed line.
- **b.** Consensus maps (low-pass filtered to 9 Å) are shown as central slices (sum of 10) viewed from the side; the *in situ* map of *Xu and colleagues* is also shown for comparison.

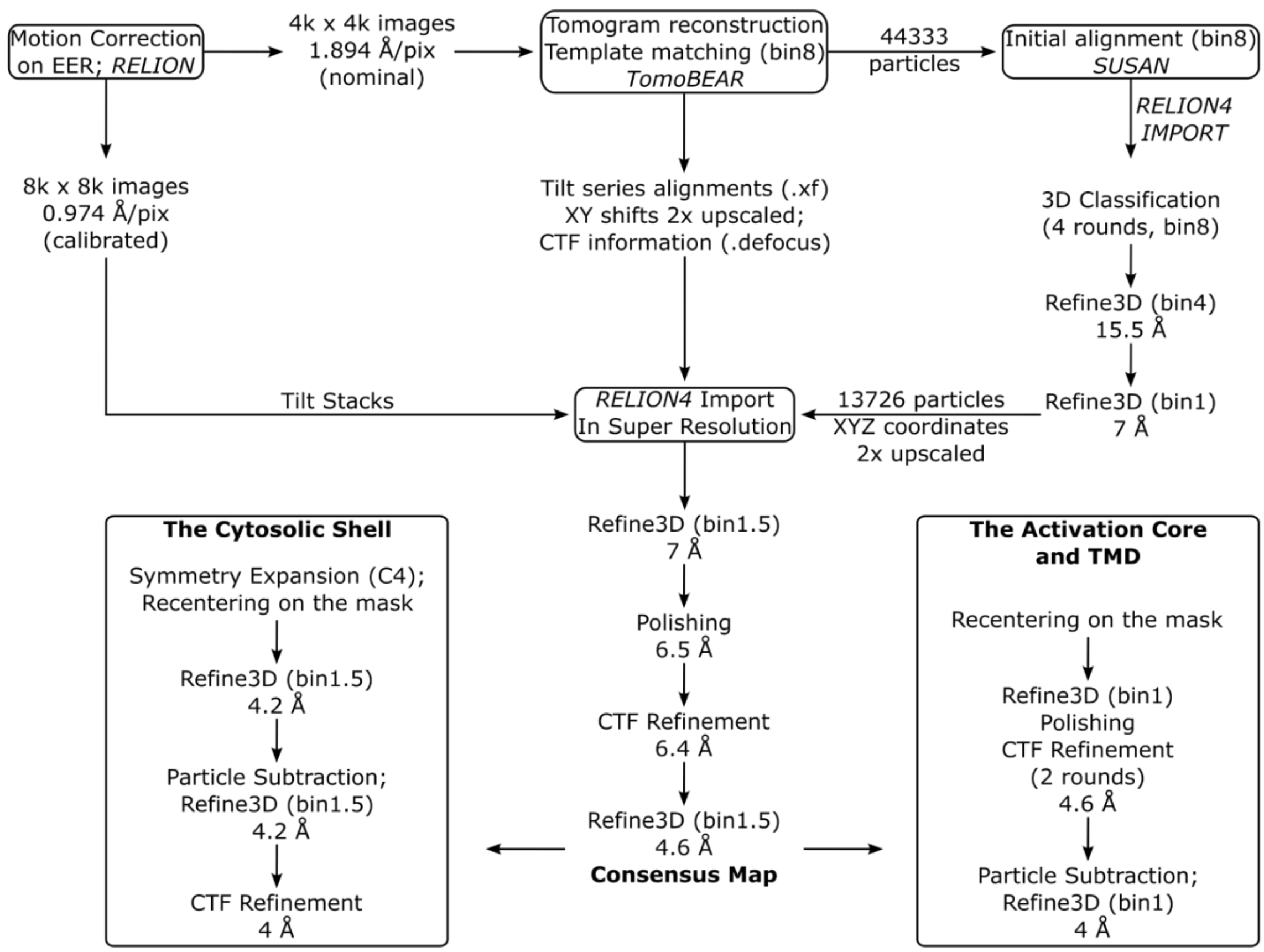

Supplementary Figure 33. Data processing flowchart for the EGTA-ET dataset.

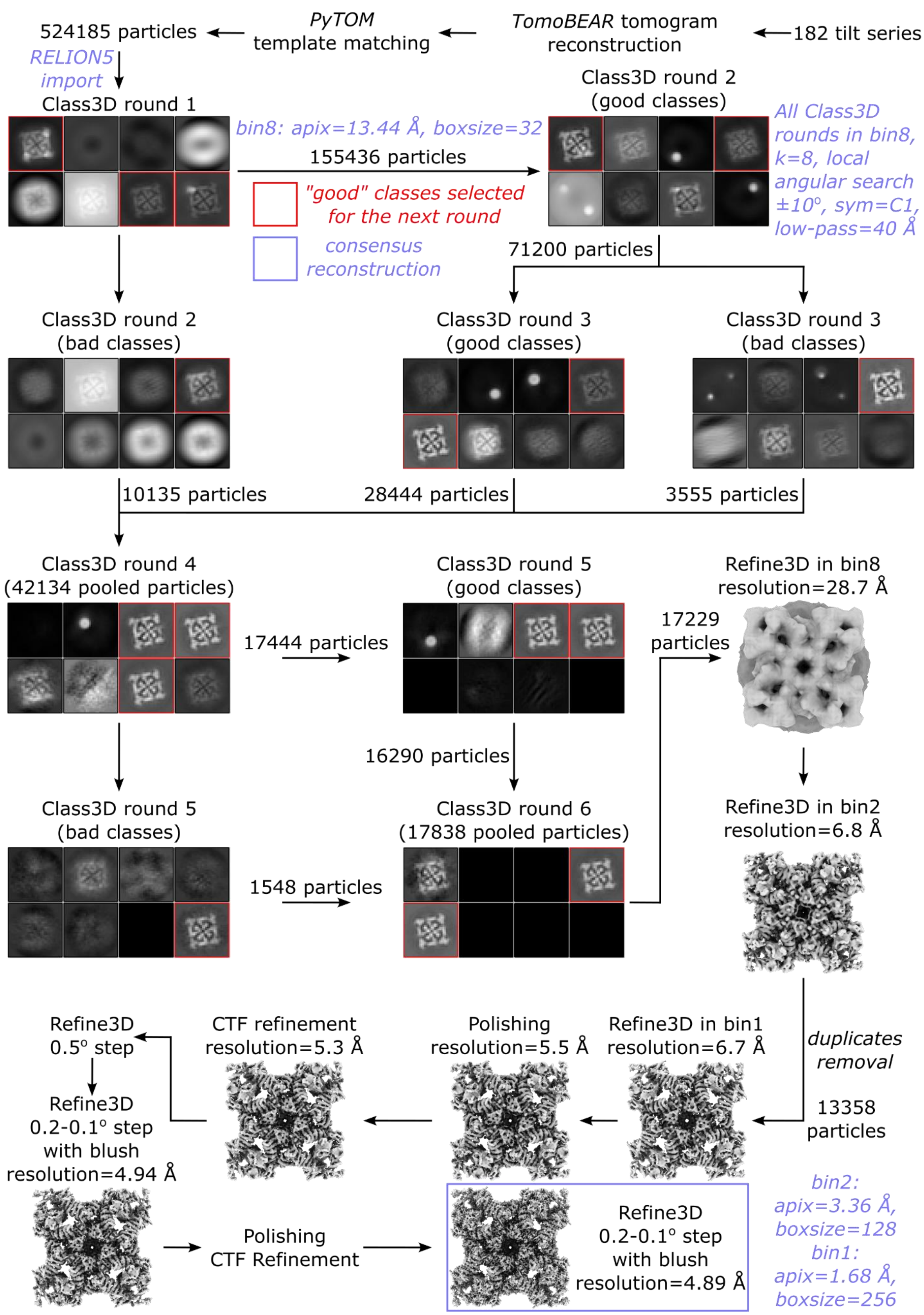

Supplementary Figure 34. Data processing flowchart for the ActMix-ET dataset.

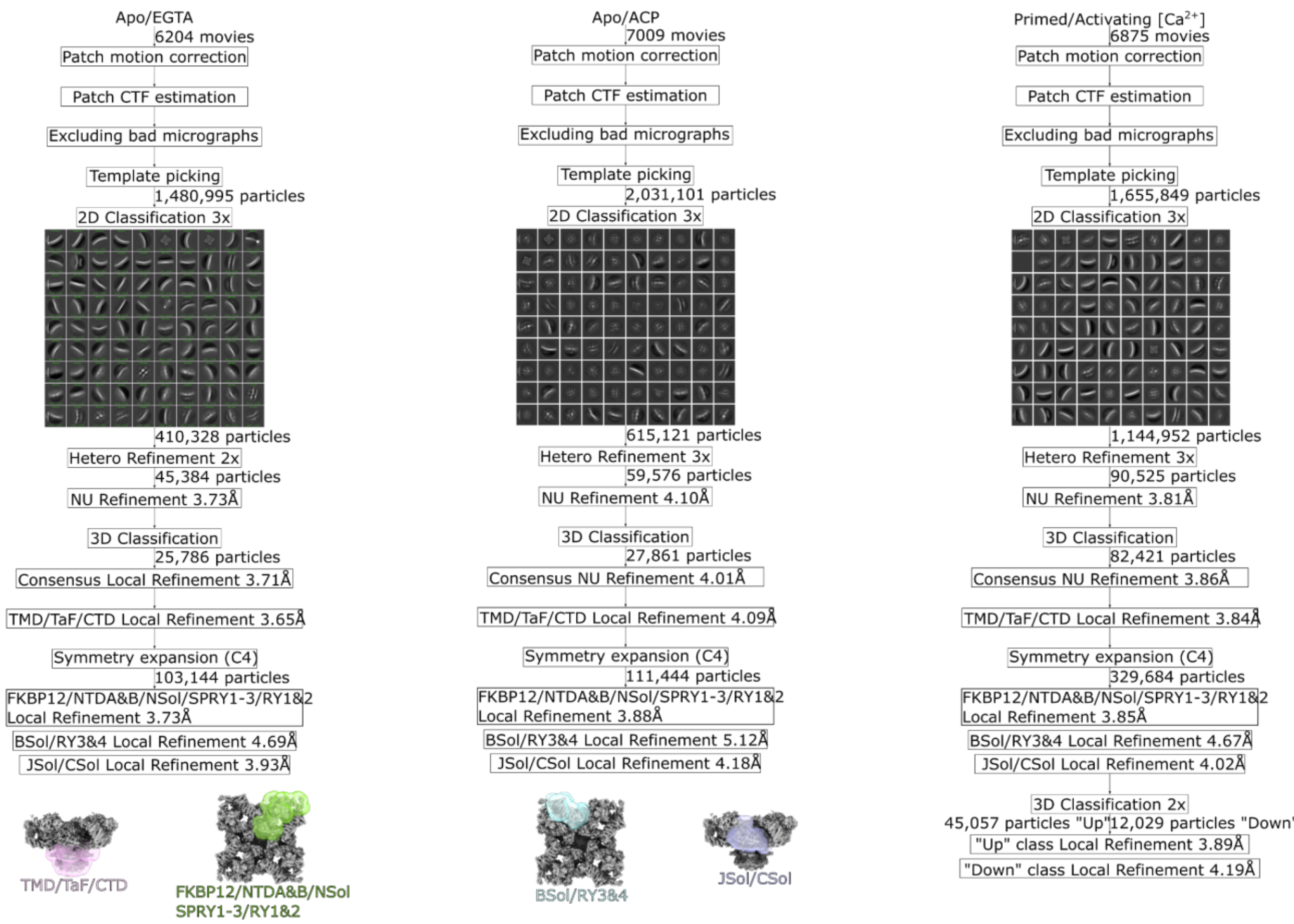

Supplementary Figure 35. Data processing flowchart for the EGTA, ACP, and Activating [Ca^{2+}] datasets. All steps were performed in CryoSPARC.

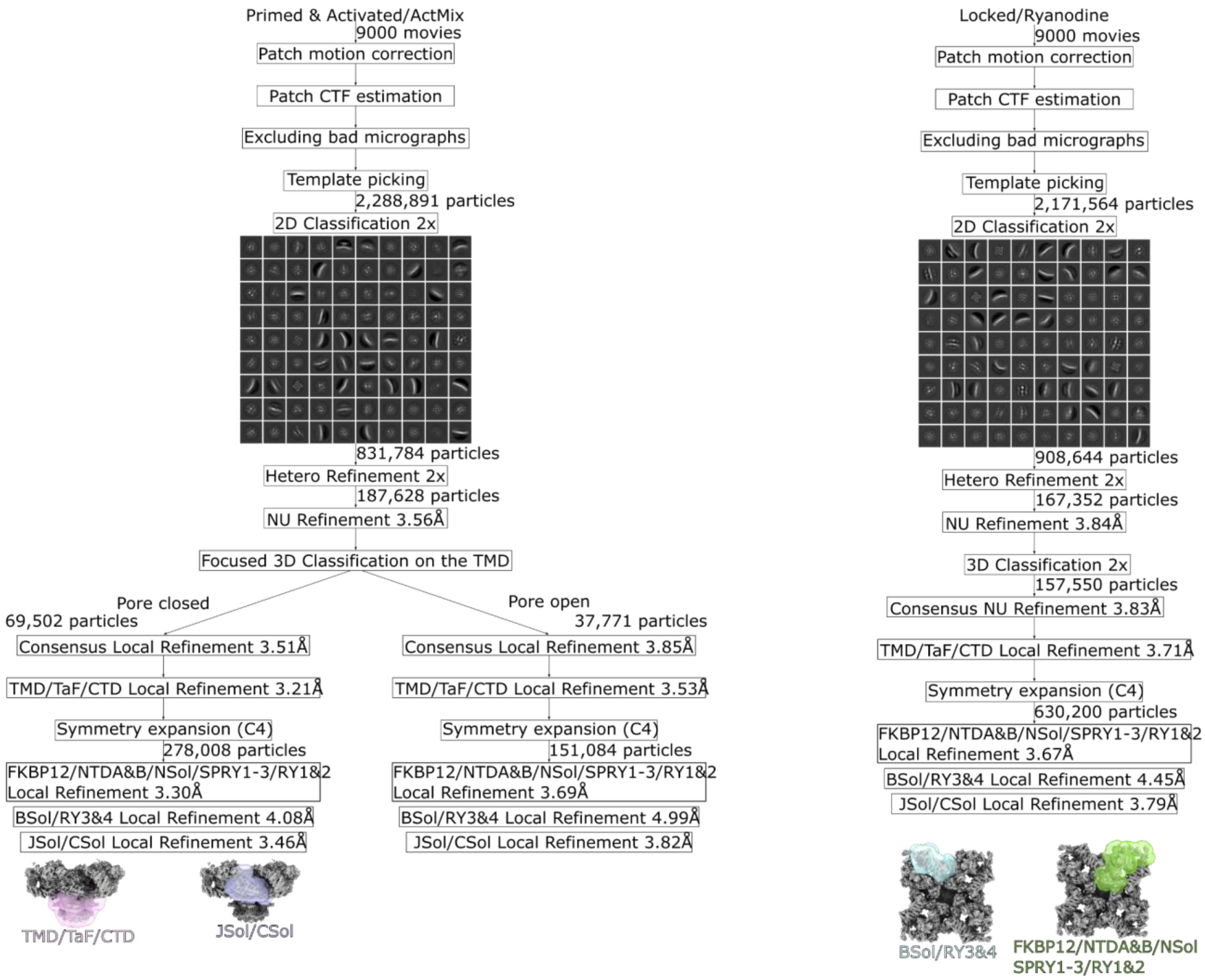

Supplementary Figure 36. Data processing flowchart for the Ca^{2+}/ACP/caffeine and Ryanodine datasets. All steps were performed in CryoSPARC.
