# Supplementary material for: Ligand-induced activation of RyR1 in native membranes
Source: Nat Commun. 2026 Sep 24;17:10075. doi: 10.1038/s41467-026-75504-9 (PMC13612463; doi:10.1038/s41467-026-75504-9)
Supplement: Supplementary file 2 [file 41467_2026_75504_MOESM2_ESM.pdf]

nature portfolio

Corresponding author(s): Mikhail Kudryashev

Last updated by author(s): Jun 10, 2026

Reporting Summary

Nature Portfolio wishes to improve the reproducibility of the work that we publish. This form provides structure for consistency and transparency in reporting. For further information on Nature Portfolio policies, see our Editorial Policies and the Editorial Policy Checklist.

Statistics

For all statistical analyses, confirm that the following items are present in the figure legend, table legend, main text, or Methods section.

n/a	Confirmed	
☐	☒	The exact sample size (*n*) for each experimental group/condition, given as a discrete number and unit of measurement
☐	☒	A statement on whether measurements were taken from distinct samples or whether the same sample was measured repeatedly
☐	☒	The statistical test(s) used AND whether they are one- or two-sided<br>*Only common tests should be described solely by name; describe more complex techniques in the Methods section.*
☒	☐	A description of all covariates tested
☐	☒	A description of any assumptions or corrections, such as tests of normality and adjustment for multiple comparisons
☐	☒	A full description of the statistical parameters including central tendency (e.g. means) or other basic estimates (e.g. regression coefficient) AND variation (e.g. standard deviation) or associated estimates of uncertainty (e.g. confidence intervals)
☐	☒	For null hypothesis testing, the test statistic (e.g. *F*, *t*, *r*) with confidence intervals, effect sizes, degrees of freedom and *P* value noted<br>*Give P values as exact values whenever suitable.*
☒	☐	For Bayesian analysis, information on the choice of priors and Markov chain Monte Carlo settings
☒	☐	For hierarchical and complex designs, identification of the appropriate level for tests and full reporting of outcomes
☒	☐	Estimates of effect sizes (e.g. Cohen's *d*, Pearson's *r*), indicating how they were calculated

Our web collection on statistics for biologists contains articles on many of the points above.

Software and code

Policy information about availability of computer code

Data collection	SerialEM v.4.0, PaceTOMO v1.8, Thermo Scientific Tomography 5 (v5.1), Thermo Scientific EPU v3.1
Data analysis	ChimeraX v.1.5, Coot v.0.8, CryoSPARC v.4.6, Dynamo v.1.1.558, Phenix v.1.20, IMOD v.4.11, RELION-4.0, RELION-5.0, SUSAN, TomoBEAR v0.6.0, ISOLDE v1.5, MATLAB 2021a, Python 3.8.1, pytom-match-pick v0.7.4, MotionCor2 v1.6.4, Gctf v1.18, HOLE v2.2.005, MODELLER v10.8, PISA (web version), GROMACS 2025.3

For manuscripts utilizing custom algorithms or software that are central to the research but not yet described in published literature, software must be made available to editors and reviewers. We strongly encourage code deposition in a community repository (e.g. GitHub). See the Nature Portfolio guidelines for submitting code & software for further information.

Data

Policy information about availability of data

All manuscripts must include a data availability statement. This statement should provide the following information, where applicable:

- Accession codes, unique identifiers, or web links for publicly available datasets
- A description of any restrictions on data availability
- For clinical datasets or third party data, please ensure that the statement adheres to our policy

Previously reported atomic coordinates of RyR1 used for analysis in this study are available in the Protein Data Bank (PDB) under accession codes:
5TB0 [https://doi.org/10.2210/pdb5tb0/pdb] (Apo-state RyR1, EGTA, consensus);

5TB1 [https://doi.org/10.2210/pdb5tb1/pdb] (Apo-state RyR1, EGTA, class 1);
5TB2 [https://doi.org/10.2210/pdb5tb2/pdb] (Apo-state RyR1, EGTA, class 2);
5TB3 [https://doi.org/10.2210/pdb5tb3/pdb] (Apo-state RyR1, EGTA, class 3);
5TB4 [https://doi.org/10.2210/pdb5tb4/pdb] (Apo-state RyR1. EGTA, class 4);
7K0T [https://doi.org/10.2210/pdb7k0t/pdb] (Apo-state RyR1, ACP);
8SEN [https://doi.org/10.2210/pdb8sen/pdb] (Apo-state RyR1, EGTA);
7TDG [https://doi.org/10.2210/pdb7TDG/pdb] (Inactivated-state RyR1, consensus);
7TDJ [https://doi.org/10.2210/pdb7TDJ/pdb] (Inactivated-state RyR1, class 1);
7TDI [https://doi.org/10.2210/pdb7TDI/pdb] (Inactivated-state RyR1, class 2);
7TDK [https://doi.org/10.2210/pdb7TDK/pdb] (Inactivated-state RyR1, class 3);
5T15 [https://doi.org/10.2210/pdb5T15/pdb] (Primed-state RyR1, 30 uM Ca2+, consensus);
5T9M [https://doi.org/10.2210/pdb5T9M/pdb] (Primed-state RyR1, 30 uM Ca2+, class 1);
5T9N [https://doi.org/10.2210/pdb5T9N/pdb] (Primed-state RyR1, 30 uM Ca2+, class 2);
5T9R [https://doi.org/10.2210/pdb5T9R/pdb] (Primed-state RyR1, 30 uM Ca2+, class 3);
5T9S [https://doi.org/10.2210/pdb5T9S/pdb] (Primed-state RyR1, 30 uM Ca2+, class 4);
5TAP [https://doi.org/10.2210/pdb5TAP/pdb] (Primed-state RyR1, CaffATP/EGTA, consensus);
5TAS [https://doi.org/10.2210/pdb5TAS/pdb] (Primed-state RyR1, CaffATP/EGTA, class 1);
5TAT [https://doi.org/10.2210/pdb5TAT/pdb] (Primed-state RyR1, CaffATP/EGTA, class 2);
5TAU [https://doi.org/10.2210/pdb5TAU/pdb] (Primed-state RyR1, CaffATP/EGTA, class 3);
5TAV [https://doi.org/10.2210/pdb5TAV/pdb] (Primed-state RyR1, CaffATP/EGTA, class 4);
5TAN [https://doi.org/10.2210/pdb5TAM/pdb] (Primed-state RyR1, Caffeine/ATP/Ca2+ dataset, class 3);
5TAM [https://doi.org/10.2210/pdb5TAN/pdb] (Primed-state RyR1, Caffeine/ATP/Ca2+ dataset, class 4);
5TAQ [https://doi.org/10.2210/pdb5TAQ/pdb] (Primed-state RyR1, Caffeine/ATP/Ca2+ dataset, class 3&4);
7M6A [https://doi.org/10.2210/pdb7M6A/pdb] (Primed-state RyR1, proteoliposomes);
5T9V [https://doi.org/10.2210/pdb5T9V/pdb] (Activated-state RyR1, Caffeine/ATP/Ca2+ dataset, class 1);
5TA3 [https://doi.org/10.2210/pdb5TA3/pdb] (Activated-state RyR1, Caffeine/ATP/Ca2+ dataset, class 2);
5TAL [https://doi.org/10.2210/pdb5TAL/pdb] (Activated-state RyR1, Caffeine/ATP/Ca2+ dataset, class 1&2);
6M2W [https://doi.org/10.2210/pdb6M2W/pdb] (Activated-state RyR1, Ca2+/Caffeine/ATP/CaM1234/CHL dataset);
7CF9 [https://doi.org/10.2210/pdb7CF9/pdb] (Activated-state RyR1, Ca2+/CHL dataset);
7M6L [https://doi.org/10.2210/pdb7M6L/pdb] (Activated-state RyR1, proteoliposomes);
7T65 [https://doi.org/10.2210/pdb7T65/pdb] (Activated-state RyR1, Y523S mutant);
7TDH [https://doi.org/10.2210/pdb7TDH/pdb] (Activated-state RyR1, with AMP-PCP and high Ca2+);
5TAW [https://doi.org/10.2210/pdb5TAW/pdb] (Locked-state, ryanodine dataset, consensus);
5TAX [https://doi.org/10.2210/pdb5TAX/pdb] (Locked-state, ryanodine dataset, class 1);
5TAY [https://doi.org/10.2210/pdb5TAY/pdb] (Locked-state, ryanodine dataset, class 2);
5TAZ [https://doi.org/10.2210/pdb5TAZ/pdb] (Locked-state, ryanodine dataset, class 3);
For the structures of RyR1 in native SR membrane determined in this study, atomic coordinates have been deposited in the Protein Data Bank (PDB) and cryo-EM maps in the Electron Microscopy Data Bank (EMDB) under accession codes:
9S5F [https://doi.org/10.2210/pdb9S5F/pdb] (Apo-state RyR1, EGTA-ET dataset, consensus);
EMD-54597 [https://www.ebi.ac.uk/pdbe/entry/emdb/EMD-54597] (Apo-state RyR1, EGTA-ET dataset, composite map);
EMD-54587 [https://www.ebi.ac.uk/pdbe/entry/emdb/EMD-54587] (Apo-state RyR1, EGTA-ET dataset, focused map);
EMD-54589 [https://www.ebi.ac.uk/pdbe/entry/emdb/EMD-54589] (Apo-state RyR1, EGTA-ET dataset, focused map);
EMD-54592 [https://www.ebi.ac.uk/pdbe/entry/emdb/EMD-54592] (Apo-state RyR1, EGTA-ET dataset, consensus map);
EMD-54596 [https://www.ebi.ac.uk/pdbe/entry/emdb/EMD-54596] (Apo-state RyR1, EGTA-ET dataset, dimer map);
9S5A [https://doi.org/10.2210/pdb9S5A/pdb] (Apo-state RyR1, EGTA-ET dataset, “Down” class);
EMD-54593 [https://www.ebi.ac.uk/pdbe/entry/emdb/EMD-54593] (Apo-state RyR1, EGTA-ET dataset, “Down” class);
9S5C [https://doi.org/10.2210/pdb9S5C/pdb] (Apo-state RyR1, EGTA-ET dataset, “Up” class);
EMD-54594 [https://www.ebi.ac.uk/pdbe/entry/emdb/EMD-54594] (Apo-state RyR1, EGTA-ET dataset, “Up” class);
9S5E [https://doi.org/10.2210/pdb9S5E/pdb] (Apo-state RyR1, EGTA-ET dataset, “Tight” class);
EMD-54595 [https://www.ebi.ac.uk/pdbe/entry/emdb/EMD-54595] (Apo-state RyR1, EGTA-ET dataset, “Tight” class);
9S16 [https://doi.org/10.2210/pdb9S16/pdb] (Apo-state RyR1, EGTA dataset, consensus);
EMD-54445 [https://www.ebi.ac.uk/pdbe/entry/emdb/EMD-54445] (Apo-state RyR1, EGTA dataset, composite map);
EMD-54431 [https://www.ebi.ac.uk/pdbe/entry/emdb/EMD-54431] (Apo-state RyR1, EGTA dataset, focused map);
EMD-54433 [https://www.ebi.ac.uk/pdbe/entry/emdb/EMD-54433] (Apo-state RyR1, EGTA dataset, focused map);
EMD-54435 [https://www.ebi.ac.uk/pdbe/entry/emdb/EMD-54435] (Apo-state RyR1, EGTA dataset, focused map);
EMD-54437 [https://www.ebi.ac.uk/pdbe/entry/emdb/EMD-54437] (Apo-state RyR1, EGTA dataset, focused map);
EMD-54438 [https://www.ebi.ac.uk/pdbe/entry/emdb/EMD-54438] (Apo-state RyR1, EGTA dataset, consensus map);
9S1H [https://doi.org/10.2210/pdb9S1H/pdb] (Apo-state RyR1, ACP dataset, consensus);
EMD-54454 [https://www.ebi.ac.uk/pdbe/entry/emdb/EMD-54454] (Apo-state RyR1, ACP dataset, composite map);
EMD-54450 [https://www.ebi.ac.uk/pdbe/entry/emdb/EMD-54450] (Apo-state RyR1, ACP dataset, focused map);
EMD-54451 [https://www.ebi.ac.uk/pdbe/entry/emdb/EMD-54451] (Apo-state RyR1, ACP dataset, focused map);
EMD-54452 [https://www.ebi.ac.uk/pdbe/entry/emdb/EMD-54452] (Apo-state RyR1, ACP dataset, focused map);
EMD-54453 [https://www.ebi.ac.uk/pdbe/entry/emdb/EMD-54453] (Apo-state RyR1, ACP dataset, focused map);
EMD-54449 [https://www.ebi.ac.uk/pdbe/entry/emdb/EMD-54449] (Apo-state RyR1, ACP dataset, consensus map);
9S2S [https://doi.org/10.2210/pdb9S2S/pdb] (Primed-state RyR1, ActCa dataset, consensus);
EMD-54509 [https://www.ebi.ac.uk/pdbe/entry/emdb/EMD-54509] (Primed-state RyR1, ActCa dataset, composite map);
EMD-54501 [https://www.ebi.ac.uk/pdbe/entry/emdb/EMD-54501] (Primed-state RyR1, ActCa dataset, focused map);
EMD-54502 [https://www.ebi.ac.uk/pdbe/entry/emdb/EMD-54502] (Primed-state RyR1, ActCa dataset, focused map);
EMD-54503 [https://www.ebi.ac.uk/pdbe/entry/emdb/EMD-54503] (Primed-state RyR1, ActCa dataset, focused map);

EMD-54504 [https://www.ebi.ac.uk/pdbe/entry/emdb/EMD-54504] (Primed-state RyR1, ActCa dataset, focused map);
EMD-54505 [https://www.ebi.ac.uk/pdbe/entry/emdb/EMD-54505] (Primed-state RyR1, ActCa dataset, consensus map);
EMD-54507 [https://www.ebi.ac.uk/pdbe/entry/emdb/EMD-54507] (Primed-state RyR1, ActCa dataset, dimer map);
9S2R [https://doi.org/10.2210/pdb9S2R/pdb] (Primed-state RyR1, ActCa dataset, "Up" class);
EMD-54508 [https://www.ebi.ac.uk/pdbe/entry/emdb/EMD-54508] (Primed-state RyR1, ActCa dataset, "Up" class);
9S5Z [https://doi.org/10.2210/pdb9S5Z/pdb] (Primed-state RyR1, ActMix-ET dataset, consensus);
EMD-54617 [https://www.ebi.ac.uk/pdbe/entry/emdb/EMD-54617] (Primed-state RyR1, ActMix-ET dataset, composite map);
EMD-54598 [https://www.ebi.ac.uk/pdbe/entry/emdb/EMD-54598] (Primed-state RyR1, ActMix-ET dataset, focused map);
EMD-54600 [https://www.ebi.ac.uk/pdbe/entry/emdb/EMD-54600] (Primed-state RyR1, ActMix-ET dataset, focused map);
EMD-54601 [https://www.ebi.ac.uk/pdbe/entry/emdb/EMD-54601] (Primed-state RyR1, ActMix-ET dataset, focused map);
EMD-54611 [https://www.ebi.ac.uk/pdbe/entry/emdb/EMD-54611] (Primed-state RyR1, ActMix-ET dataset, focused map);
EMD-54612 [https://www.ebi.ac.uk/pdbe/entry/emdb/EMD-54612] (Primed-state RyR1, ActMix-ET dataset, consensus map);
EMD-54614 [https://www.ebi.ac.uk/pdbe/entry/emdb/EMD-54614] (Primed-state RyR1, ActMix-ET dataset, dimer map);
9S5W [https://doi.org/10.2210/pdb9S5W/pdb] (Primed-state RyR1, ActMix-ET dataset, "Down" class);
EMD-54615 [https://www.ebi.ac.uk/pdbe/entry/emdb/EMD-54615] (Primed-state RyR1, ActMix-ET dataset, "Down" class);
9S5Y [https://doi.org/10.2210/pdb9S5Y/pdb] (Primed-state RyR1, ActMix-ET dataset, "Up" class);
EMD-54616 [https://www.ebi.ac.uk/pdbe/entry/emdb/EMD-54616] (Primed-state RyR1, ActMix-ET dataset, "Up" class);
9S3V [https://doi.org/10.2210/pdb9S3V/pdb] (Primed-state RyR1, ActMix dataset, consensus);
EMD-54553 [https://www.ebi.ac.uk/pdbe/entry/emdb/EMD-54553] (Primed-state RyR1, ActMix dataset, composite map);
EMD-54512 [https://www.ebi.ac.uk/pdbe/entry/emdb/EMD-54512] (Primed-state RyR1, ActMix dataset, focused map);
EMD-54513 [https://www.ebi.ac.uk/pdbe/entry/emdb/EMD-54513] (Primed-state RyR1, ActMix dataset, focused map);
EMD-54514 [https://www.ebi.ac.uk/pdbe/entry/emdb/EMD-54514] (Primed-state RyR1, ActMix dataset, focused map);
EMD-54515 [https://www.ebi.ac.uk/pdbe/entry/emdb/EMD-54515] (Primed-state RyR1, ActMix dataset, focused map);
EMD-54516 [https://www.ebi.ac.uk/pdbe/entry/emdb/EMD-54516] (Primed-state RyR1, ActMix dataset, consensus map);
EMD-54517 [https://www.ebi.ac.uk/pdbe/entry/emdb/EMD-54517] (Activated and Primed-state RyR1, ActMix dataset, dimer map);
9S3U [https://doi.org/10.2210/pdb9S3U/pdb] (Primed-state RyR1, ActMix dataset, "Down" class);
EMD-54552 [https://www.ebi.ac.uk/pdbe/entry/emdb/EMD-54552] (Primed-state RyR1, ActMix dataset, "Down class");
9S4N [https://doi.org/10.2210/pdb9S4N/pdb] (Activated-state RyR1, ActMix dataset, consensus);
EMD-54570 [https://www.ebi.ac.uk/pdbe/entry/emdb/EMD-54570] (Activated-state RyR1, ActMix dataset, composite map);
EMD-54554 [https://www.ebi.ac.uk/pdbe/entry/emdb/EMD-54554] (Activated-state RyR1, ActMix dataset, focused map);
EMD-54555 [https://www.ebi.ac.uk/pdbe/entry/emdb/EMD-54555] (Activated-state RyR1, ActMix dataset, focused map);
EMD-54557 [https://www.ebi.ac.uk/pdbe/entry/emdb/EMD-54557] (Activated-state RyR1, ActMix dataset, focused map);
EMD-54562 [https://www.ebi.ac.uk/pdbe/entry/emdb/EMD-54562] (Activated-state RyR1, ActMix dataset, focused map);
EMD-54568 [https://www.ebi.ac.uk/pdbe/entry/emdb/EMD-54568] (Activated-state RyR1, ActMix dataset, consensus map);
9S4L [https://doi.org/10.2210/pdb9S4L/pdb] (Activated-state RyR1, ActMix dataset, "Down" class);
EMD-54569 [https://www.ebi.ac.uk/pdbe/entry/emdb/EMD-54569] (Activated-state RyR1, ActMix dataset, "Down class");
9S55 [https://doi.org/10.2210/pdb9S55/pdb] (Locked-state RyR1, Ryanodine dataset, consensus);
EMD-54590 [https://www.ebi.ac.uk/pdbe/entry/emdb/EMD-54590] (Locked-state RyR1, Ryanodine dataset, composite map);
EMD-54571 [https://www.ebi.ac.uk/pdbe/entry/emdb/EMD-54571] (Locked-state RyR1, Ryanodine dataset, focused map);
EMD-54572 [https://www.ebi.ac.uk/pdbe/entry/emdb/EMD-54572] (Locked-state RyR1, Ryanodine dataset, focused map);
EMD-54573 [https://www.ebi.ac.uk/pdbe/entry/emdb/EMD-54573] (Locked-state RyR1, Ryanodine dataset, focused map);
EMD-54574 [https://www.ebi.ac.uk/pdbe/entry/emdb/EMD-54574] (Locked-state RyR1, Ryanodine dataset, focused map);
EMD-54575 [https://www.ebi.ac.uk/pdbe/entry/emdb/EMD-54575] (Locked-state RyR1, Ryanodine dataset, consensus map);
EMD-54580 [https://www.ebi.ac.uk/pdbe/entry/emdb/EMD-54580] (Locked-state RyR1, Ryanodine dataset, dimer map);
9S54 [https://doi.org/10.2210/pdb9S54/pdb] (Locked-state RyR1, Ryanodine dataset, "Down" class);
EMD-54588 [https://www.ebi.ac.uk/pdbe/entry/emdb/EMD-54588] (Locked-state RyR1, Ryanodine dataset, "Down" class);

Research involving human participants, their data, or biological material

Policy information about studies with human participants or human data. See also policy information about sex, gender (identity/presentation), and sexual orientation and race, ethnicity and racism.

Reporting on sex and gender	N/A
Reporting on race, ethnicity, or other socially relevant groupings	N/A
Population characteristics	N/A
Recruitment	N/A
Ethics oversight	N/A

Note that full information on the approval of the study protocol must also be provided in the manuscript.

Field-specific reporting

For a reference copy of the document with all sections, see nature.com/documents/nr-reporting-summary-flat.pdf

Life sciences study design

All studies must disclose on these points even when the disclosure is negative.

Sample size	No statistical methods were used to predetermine sample size. The number of tilt series collected for each EHD2 consutrcut (full-length and N-terminally truncated) and for the sections of resin embedded cells were collected within the available data collection session.
Data exclusions	No data were excluded from the analyses unless stated otherwise in the Methods. All available particles, channels, and models that passed predefined quality criteria (e.g. classification, refinement, and convergence thresholds) were included. Where data were filtered (for example, to require at least one neighbor for inclusion in the pairing analysis), the criteria are described in the Methods.
Replication	The number of replicates is indicated in the figure legends, where applicable. For the potential energy analysis of single-channel and dimer structural models, three independent energy minimizations were performed per functional state and configuration (single vs dimer), yielding n=3 potential energy values per condition.
Randomization	The experiments were not randomized. Particles extracted from the cryo-EM datasets were randomly divided into two half datasets and processed independently to estimate the resolution. For dimer class clustering analysis, class labels were randomly permuted across all channels while preserving the observed graph structure, and the test statistic (mean same-class neighbor fraction per channel in the target class) was recomputed for 10,000 permutations.
Blinding	None of the results presented require blinding since they are not subjective.

Reporting for specific materials, systems and methods

We require information from authors about some types of materials, experimental systems and methods used in many studies. Here, indicate whether each material, system or method listed is relevant to your study. If you are not sure if a list item applies to your research, read the appropriate section before selecting a response.

Materials & experimental systems

n/a	Involved in the study	
☒	☐	Antibodies
☒	☐	Eukaryotic cell lines
☒	☐	Palaeontology and archaeology
☒	☐	Animals and other organisms
☒	☐	Clinical data
☒	☐	Dual use research of concern
☒	☐	Plants

Methods

n/a	Involved in the study	
☒	☐	ChIP-seq
☒	☐	Flow cytometry
☒	☐	MRI-based neuroimaging

Plants

Seed stocks	N/A
Novel plant genotypes	N/A
Authentication	N/A
