# Supplementary material for: Ligand-induced activation of RyR1 in native membranes
Source: Nat Commun. 2026 Sep 24;17:10075. doi: 10.1038/s41467-026-75504-9 (PMC13612463; doi:10.1038/s41467-026-75504-9)
Supplement: Supplementary file 3 [file 41467_2026_75504_MOESM3_ESM.pdf]

nature portfolio

Peer Review File

Ligand-induced activation of RyR1 in native membranes

Corresponding Author: Professor Mikhail Kudryashev

This file contains all reviewer reports in order by version, followed by all author rebuttals in order by version.

Version 0:

Reviewer comments:

Reviewer #1

(Remarks to the Author)
This is an interesting work from the Kudryashev lab presenting several reconstructions of RyR1 in native membranes, leading to new structural and functional insights on the channel. I have three main concerns indicated below and multiple specific comments, but I also want to point out that its main strengths: 1) presenting the channels in their natural environment, 2) presenting the structure of RyR dimers, and 3) an extensive comparative analysis with existing structures, make this manuscript an excellent contribution to the field.

MAIN CONCERNS

The manuscript is a bit misleading in the sense that it would appear that this corresponds to the physiological activation of RyR1 in membranes, but one has to distinguish between A) the coupled gating caused by addition of Ca2+ to artificial bilayers in an experimental setup, and B) the physiological activation of RyR1, NOT caused by Ca2+, but by skeletal-type excitation contraction coupling, i.e., by the DHPRs being activated by depolarization, and these activated DHPRs activating RyR1s via direct contact, in the process of conformational coupling. The sentence in the abstract: “Synchronized calcium release through arrays of the type-1 ryanodine receptor RyR1 is fundamental to skeletal muscle excitation-contraction coupling. This synchrony is achieved through a phenomenon known as “coupled gating”, where the activation of one channel promotes the opening of its neighbors.” is incorrect; the synchrony during physiological function is achieved by the depolarization wave. Likewise, the title gives the impression that depolarization and DHPR will be presented in the work, but the activator is Ca2+, using the same activating conditions as these used for purified channels. An accurate title needs to include the fact that Ca2+ is the activator.

There is exaggeration of differences between the channels in membranes and the purified ones, for example in the Abstract:” These lattice interactions constrain RyR1 activation to a large-scale, continuous in-plane rotation of its cytosolic shell, a mechanism distinct from the tilting motions observed in isolated receptors.” In-plane rotations are known and have been described for isolated receptors, while tilting motions are also observed in this work. The results need to be more accurately worded to not create unnecessary confusion, as later the figures do not fully support such statements.

There is indeed a different range of Flexiontilt and FlexionRot angles in purified versus reconstructions from RyRs in the membrane. But the interpretation that physical packing affects the rotation, although interesting, needs to be worded with caution. The reconstructions include RyRs that are touching the neighbors but also single RyRs - the latter would not be affected by packing restrictions. Another potential explanation could be that unresolved protein partner(s) is/are affecting the conformation. The presence of FKBP has an influence in the Up/Down conformations of the cytosolic shell.

SPECIFIC COMMENTS

The protocol should have produced triads, and should have revealed RyR-DHPR complexes as in ref #26. Although this is not detrimental to the presented work, why DHPR are not visible in this preparation warrants an explanation. Also, the mention of calsequestrin in the introduction creates the unfulfilled expectation that this protein partner will be shown. Likewise, lines 89-92 mention that “To bridge this gap, we determined the structures of RyR1 by single-particle cryo-EM (SPA) and subtomogram averaging (StA) from cryo-ET data in its native membrane environment, with the set of native associated protein partners to define the structural basis of coupled gating.” This should be reworded because partner

proteins are not shown. The absence of CaM bound to the channel is also surprising.

Line 97: “We show that these lattice interactions constrain the channel's activation pathway to a rotation-dominant motion…” Please add “in plane” before “rotation-dominant motion” for clarity.

Line 143: “Activating ligands shifted populations predominantly to the “Up” conformation”. This part is confusing. 1) Looking at Figure S13, it appears that these follow the same directions as published structures, i.e. Apo which has FlexionTilt of 2-5 and activation brings to a FlexionTilt around 0 in actMix. Thus going to a smaller value should be Down. 2) Up would be surprising considering the consensus among all other published structures of purified RyR, where the effect of Ca2+ moves the shell in the “Down” direction. It would be unlikely that the membrane (far away from the Ca+ binding site) would induce an opposite effect to that of Ca2+. Is it possible that Up and Down is defined relative to each condition, instead of all the dataset? 3) the angle was calculated between the axis and the vertical, while normally this angle is considered with respect to the horizontal plane. This would yield opposite results. 4) Flexion Rot also depends on the X axis which is not well defined. 5) The reviewer recommends reconsidering the FlexionTilt angle with respect to the horizontal plane as in other works, and to provide a schematics of the X / Z axes, and the Flexion Tilt / FlexionRot axes, potentially in Fig. S13.

Line 147: “In the EGTA-ET dataset, we also observed some variability in the EF1&2 hand domain conformation (Fig. S10) as well as its unexpected orientation relative to the S2S3 helical bundle domain of the neighbouring protomer (Fig. S11).” Please explain in which way the orientation is unexpected; this is not obvious from Fig. S10. Also, please comment how the orientations compare with respect to these described in Ref #17.

Lines 198-199” In those studies, activation was associated with an extensive ~5.7° downward tilt and a ~5.1° rotation14”. Please double check that this is actually mentioned in the reference.

Lines 199-202: “In contrast, our analysis of RyR1 structures in native membranes revealed a cumulative in-plane rotation of ~6.3° during activation (Fig. 2A–F, Fig. S12, S12A-D)”. 1) Figure S12 does not show in plane angles. 2) There is not so much contrast: 6.3 degrees is not that different from 5.1 degrees found by others.

Lines 204-209: “During priming, the RyR1 cytosolic shell tilts by ~2.8° and rotates by ~3.9° (Fig. S13B-C), while full activation is characterized primarily by additional rotation of ~2.4° without further significant tilt (Fig. S13D). In contrast, purified RyR1 structures exhibit greater cumulative tilting of ~4.5° during priming, and an additional ~1.1° tilt upon activation. Thus, the total difference in cumulative tilting between purified and native RyR1 channels is ~3°, emphasizing that native environment constraints substantially limit the vertical domain movements (Fig. 2G).” 1) Please explain where the 3 degrees comes from -the cumulative (in plane + vertical) rotation is 10.8 degrees for purified protein and 9.1 for protein within the membrane = 1.7 degrees difference = ~10% difference, which would be less dramatic.

Figure S15 B-D. Dimension 1 and 2 are arbitrary; It would help reproducibility if this information could be displayed with more context, showing the directionality of such changes. The figure would greatly benefit if purified RyRs and SR-RyRs were in different panels.

Methods, line 446. “1 mM CaCl2 and EGTA stock solutions were added to the sample to a final concentration of 30 uM Ca2+ determined with Maxchelator”. It is not clear if Mg2+ and ACP were included in the Maxchelator calculation but they should be, as ACP binds Ca2+ and Mg2+ reducing their free concentrations. Also, free Mg2+ should be stated: both free Ca2+ and Mg2+ concentration are critical to determine the state of the channel, and need to be considered for comparison with the existing structures.

Table S1: Structures/classes %: please add the % of the different subclasses.

Figure S11: Please indicate the identity of the 4 residues of interest at least in one of the panels. It is difficult to extract the main idea from the different pairwise comparisons; it could help to use the same “reference” reconstruction for the different panels. In addition, large distance differences do not reflect well in the panels. For example in panel E, the E4075-R4736 distance does not seem to be 8 Å different as indicated in panel I. Perhaps it is the perspective but it would be worth to double check.

Figure S15: Very interesting figure, but the names of the atomic models are superimposed in some of the panels, becoming illegible. Also, please add the definition of the color coding.

Figure S17: Please indicate the residue corresponding to wt and mutation

Figure S18: Probably this is the same flowchart for one of the ActMix datasets, please indicate so in the legend. Also, please describe the “Particle Subtraction” in the legend or in the text

Reviewer #2

(Remarks to the Author)
The work presents a valuable investigation into the high-resolution structures of RyR1 in native membranes using cryo-EM STA and SPA, successfully revealed the conformational transitions underlying RyR1 channel opening. The capture of

multiple functional states along the activation pathway provides important insights into the mechanism of synchronized Ca^{2+} release in skeletal muscle, which represents a notable advance in understanding RyR1 biology.
However, several critical limitations—related to technical clarity, sample validity, structural resolution, and data presentation—undermine the robustness of key conclusions, particularly regarding inter-RyR1 interfaces, dimer function, and physiological relevance. Below is a detailed expansion of concerns:
1. Line 104, the phrase "following the previous reports" is misleading and uninformative. A core strength of this study is its achievement of high-resolution RyR1 structures in native membranes—yet the manuscript fails to highlight the key technical improvements that enabled this advance, which is critical for readers to evaluate and build upon the work. While the Methods section outlines protocols, the Results section lacks clear discussion of how the authors optimized sample preparation, cryo-EM data collection, or data processing to surpass the resolution of prior in situ RyR1 studies. Without this context, the novelty of the methodological approach is obscured.
2. Line 106, Misrepresentation of "checkerboard" organization. The claim that isolated SR microsomes exhibit a RyR1 lattice "closely resembling their native 'checkerboard' organization in intact muscle triads" is unsupported. Mammalian skeletal muscle triads feature RyR1 arranged in two tightly packed, uniformly oriented rows on the SR, with consistent alignment of all tetramers. In contrast, Fig. S1 shows no evidence of this ordered two-row arrangement; instead, RyR1 orientation appears heterogeneous and packing is irregular. This discrepancy strongly suggests post-isolation rearrangement of RyR1 tetramers. If the authors insist on "closely resembling" native triads, they must provide side-by-side comparative diagrams of their lattice versus published intact triad structures and quantify key parameters (e.g., inter-RyR distance, tetramer orientation).
3. Potential membrane curvature artifacts. Native junctional SR in triads is a highly curved, narrow structure optimized for DHPR-RyR1 coupling. However, the SR sample in this study appear relatively flat. Membrane curvature directly influences protein conformation and lattice organization—flattening could alter RyR1's cytosolic domain orientation or inter-channel contacts. The authors must address whether this curvature change impacts structural conclusions.
3. Line 108 references "Using a combination of SPA and StA," but the manuscript provides no justification for this hybrid approach or practical details of its implementation. Readers need to understand why was a combination necessary?
4. Vague references to "previously reported structures". The manuscript repeatedly claims similarities to prior RyR1 structures without quantitative or visual validation. For all such claims, the authors must include side-by-side structural alignments (maybe in supplementary figures) of their models with the referenced structures, along with RMSD values for key domains.
5.Line 137, inadequate characterization of "Up," "Down," and "Tight" states. The structural differences between these states (Fig. S9) are visually indiscernible due to the lack of local zoomed-in panels highlighting the most dynamic regions. Additionally, the Methods mention centroid-based angle measurements, but the Results provide no clear diagrams of how these angles were calculated (e.g., axes definitions, reference points for "Up" vs. "Down" orientations).
6. Fig. S9–S11: Text labels (e.g., domain names, state designations) are difficult to read. Distance matrix plots (e.g., in Fig. S15): Protein identifiers are jumbled, making it impossible to trace arrangement of different states. Fig. S15: Protein labels in the FlexionTilt/FlexionRot plot are illegible, preventing readers from verifying trends in cytosolic shell motion. These figures must be revised for clarity, with standardized font sizes, simplified layouts, and clear legends.
7. Line 249, the authors report RyR1 dimers in five functional states, but the manuscript does not explain why dimer resolution is significantly lower than monomer resolution (e.g., is it due to conformational heterogeneity of the interface, or insufficient particle numbers?). This is critical because contact area measurements rely on these low-resolution models—readers need to know if such analyses are statistically robust.
8. Table S1 provides no information on the number of particles used for dimer reconstruction. If most particles were discarded during 3D classification, the remaining subset may not be representative of physiological RyR1 interactions.
9. Fig. S1 suggests heterogeneous inter-RyR1 contacts in isolated SR microsomes. The authors must address whether this heterogeneity can be resolved for dimer reconstruction and how it impacts conclusions about "coupled gating."
10. Missing structural determination workflows. The Methods section does not include schematic workflows for STA, SPA, and dimer reconstruction. These diagrams are essential for readers to follow how raw tomograms/ micrographs were processed into final models (e.g., particle picking, classification steps, refinement iterations).
11. Fig. 4H, the schematic of coupled gating is overly simplistic and fails to integrate key structural details. It should be revised to include: (1) Visual cues for conformational changes, (2) Labels for key domains, and (3) A timeline of events.

Version 1:

Reviewer comments:

Reviewer #1

(Remarks to the Author)
The authors addressed satisfactorily all my questions.
Based on the improvements and the scientific advance that this work represents, I recommend publication.

(Remarks on code availability)

Reviewer #2

(Remarks to the Author)
The authors have fully responded to all the questions and comments raised in my previous review. The logic, content

integrity and expression of the manuscript have been effectively improved. I am now satisfied with the revised version. The manuscript meets the publication requirements of Nature Communications and I recommend it for acceptance.

(Remarks on code availability)

Open Access This Peer Review File is licensed under a Creative Commons Attribution 4.0 International License, which permits use, sharing, adaptation, distribution and reproduction in any medium or format, as long as you give appropriate credit to the original author(s) and the source, provide a link to the Creative Commons license, and indicate if changes were made.
In cases where reviewers are anonymous, credit should be given to 'Anonymous Referee' and the source.
The images or other third party material in this Peer Review File are included in the article's Creative Commons license, unless indicated otherwise in a credit line to the material. If material is not included in the article's Creative Commons license and your intended use is not permitted by statutory regulation or exceeds the permitted use, you will need to obtain permission directly from the copyright holder.
To view a copy of this license, visit https://creativecommons.org/licenses/by/4.0/

We thank the reviewers for highlighting the strengths and the importance of our manuscript and for your detailed comments that helped to improve the interpretation and presentation of data. We expanded the Results and Discussion sections with the large update of the Methods section. Furthermore, we added five supplemental tables and sixteen supplemental figures. Furthermore, we updated the figures following the guidelines of Nature Communications.

Below, we provide a point-to-point response along with the revised text. Our comments are in blue.

Reviewer #1 (Remarks to the Author):

This is an interesting work from the Kudryashev lab presenting several reconstructions of RyR1 in native membranes, leading to new structural and functional insights on the channel. I have three main concerns indicated below and multiple specific comments, but I also want to point out that its main strengths: 1) presenting the channels in their natural environment, 2) presenting the structure of RyR dimers, and 3) an extensive comparative analysis with existing structures, make this manuscript an excellent contribution to the field.

Thank you very much for your comments and constructive suggestions.

MAIN CONCERNS

The manuscript is a bit misleading in the sense that it would appear that this corresponds to the physiological activation of RyR1 in membranes, but one has to distinguish between A) the coupled gating caused by addition of Ca2+ to artificial bilayers in an experimental setup, and B) the physiological activation of RyR1, NOT caused by Ca2+, but by skeletal-type excitation contraction coupling, i.e., by the DHPRs being activated by depolarization, and these activated DHPRs activating RyR1s via direct contact, in the process of conformational coupling. The sentence in the abstract: “Synchronized calcium release through arrays of the type-1 ryanodine receptor RyR1 is fundamental to skeletal muscle excitation-contraction coupling. This synchrony is achieved through a phenomenon known as “coupled gating”, where the activation of one channel promotes the opening of its neighbors.” is incorrect; the synchrony during physiological function is achieved by the depolarization wave.

Response: Thank you very much. We agree that the action potential and DHPR activation lead to coupled gating, but it can also be observed in planar bilayers without DHPR (reference 3 in our manuscript), and is therefore also a property of the RyR1 lattice.

We updated the abstract, now the abstract reads: “Synchronized calcium release through arrays of the type-1 ryanodine receptor RyR1 is fundamental to skeletal muscle excitation-contraction coupling. This synchrony is achieved through the mechanical coupling of RyR1s to voltage-sensing receptors DHPR that activate RyR1s in response to action potentials. The Ca^{2+} release is further enhanced through a phenomenon known as “coupled gating”, where the activation of one channel promotes the opening of its neighbours.” (beginning of the abstract)

Likewise, the title gives the impression that depolarization and DHPR will be presented in the work, but the activator is Ca2+, using the same activating conditions as these used for purified channels. An accurate title needs to include the fact that Ca2+ is the activator.

Response: We agree and modified the title to "Ligand-induced activation of RyR1 in native membranes.", as we have a combination of ligands in our report.
We also rewrote a sentence in the first paragraph of the discussion to remove the mention of the depolarization; now it reads:
"The stored potential energy lowers the activation barrier for the cluster of channels, priming them to open synchronously."

There is exaggeration of differences between the channels in membranes and the purified ones, for example in the Abstract:" These lattice interactions constrain RyR1 activation to a large-scale, continuous in-plane rotation of its cytosolic shell, a mechanism distinct from the tilting motions observed in isolated receptors." In-plane rotations are known and have been described for isolated receptors, while tilting motions are also observed in this work. The results need to be more accurately worded to not create unnecessary confusion, as later the figures do not fully support such statements.

Response: Thank you, we agree and toned the statement down, now it reads:
"These lattice interactions constrain RyR1 activation to a continuous in-plane rotation of its cytosolic shell, with less tilting motion than in the isolated receptors." (in the abstract).

We modified the last sentence of the corresponding section in the results, which reads:
"Thus, the total difference in cumulative tilting between purified and native RyR1 channels is ~3°, emphasizing that native environment constraints limit the vertical domain movements (Fig. 2G)."

We also toned down the discussion by replacing the term "paradigm" with "mechanism" in the first paragraph of the discussion and the "model" to "mechanism" in the abstract.

There is indeed a different range of Flexiontilt and FlexionRot angles in purified versus reconstructions from RyRs in the membrane. But the interpretation that physical packing affects the rotation, although interesting, needs to be worded with caution. The reconstructions include RyRs that are touching the neighbors but also single RyRs - the latter would not be affected by packing restrictions. Another potential explanation could be that unresolved protein partner(s) is/are affecting the conformation. The presence of FKBP has an influence in the Up/Down conformations of the cytosolic shell.

Response: Thank you for the insightful comment. We agree that multiple factors can play a role here, and we would like to add a clarification.

First, we realized that the naming of the angles as FlexionRot and FlexionTilt is less intuitive for readers and decided to update the notation to “In-plane rotation” and “cytosolic shell tilt”. We give credit to the original reports of Montserrat Samso’s group and describe their calculation the methods section.

About the packing: RyR1 channels form arrays *in vivo*, and some of these array interactions are lost during the SR vesicles purification. A fraction of RyR1 particles is also lost during data processing due to technical limitations. Signal in the tomograms is spatially anisotropic, as it’s influenced by thickness (ice gradient, local sample concentration, tilting), tilt series alignment (areas with fewer fiducial markers are noisier), and multiple other factors like grid contamination, variable contrast, etc. As a result, some particles aren’t picked by template matching or are “classified out” during data processing. Therefore, many of the particles in the final dataset have neighbouring particles, but these neighbouring particles aren’t part of the final dataset, as they were excluded.

We also added the particle numbers that constitute dimers into Table S1. From the particles in our single-particle datasets, the majority of their neighbours have corner contacts. For the actCa dataset, for ~82 k monomeric RyR1 particles, ~37k are dimers (89%). We could not convincingly separate individual RyR1s from RyR1s in contact. However, as the high-resolution models excellently fit all the structures of dimers, we believe that for a given functional state, our average structures are the same for monomeric RyR1 and RyR1s in a lattice.

For tomography, the processing is more elaborate, and we added Figures S28 and S29 to show that a large fraction of receptors form dimers. We refer to the new figures in the results under “A BSol-mediated interface forms the structural basis for RyR1 coupling” (lines 324 and later)

The presence of the FKBP12 influences the cytosolic shell conformation, as it lifts it more “Up”. Most of the previously determined structures that are used for comparison also have either FKBP12 or FKBP12.6 bound; we believe that the comparison to our structures, all of which have FKBP12, is fair. While we cannot exclude that additional, unresolved partners could influence conformation, we do not observe consistent ordered density near RyR1 that would suggest an additional tightly bound, structurally ordered factor across datasets. We modified the results section; now it reads:

“We therefore propose that the combination of the native interacting partners, including FKBP12 and the lateral contacts with neighbouring RyR1 tetramers in the native SR lattice restrict large-scale tilting of the cytoplasmic shell during activation” (line 313 onwards)

We modified a sentence in the discussion: “Our results define the combined impact of native lipids, FKBP12, and RyR1-RyR1 lattice contacts as stabilizers of RyR1 in the closed state, preventing spontaneous activation and ensuring precise Ca^{2+} signaling control.” (line 427 onwards)

SPECIFIC COMMENTS

The protocol should have produced triads, and should have revealed RyR-DHPR complexes as in ref #26. Although this is not detrimental to the presented work, why DHPR are not visible in this preparation warrants an explanation.

Response: Thank you, we previously used a similar protocol and found that some RyR1s are in contact with a neighbouring membrane, resembling the T-Tubule membrane (Chen and Kudryashev, EMBO Rep, 2020, reference #22). In that paper, we attempted to determine the structure of the supercomplex; we did not see DHPR in the average (Figure 1 in ref. #22). We can not make a clear statement why that was the case; the complex of RyR1 and DHPR may dissociate for a large fraction of molecules, and on average, we only get an empty membrane. Furthermore, as the putative T-tubule membranes were rarely attached to RyR1s, it is statistically unlikely to have a large number of such particles for analysis and averaging.

We modified the second last paragraph of the discussion, now it reads: "... While our preparations contained few putative T-tubules, similar to our previous report[22], they were rare, and the averages did not have T-tubule membranes and DHPR …"

Also, the mention of calsequestrin in the introduction creates the unfulfilled expectation that this protein partner will be shown.

Response: Thank you, we removed the corresponding sentence from the introduction.

Likewise, lines 89-92 mention that "To bridge this gap, we determined the structures of RyR1 by single-particle cryo-EM (SPA) and subtomogram averaging (StA) from cryo-ET data in its native membrane environment, with the set of native associated protein partners to define the structural basis of coupled gating." This should be reworded because partner proteins are not shown. The absence of CaM bound to the channel is also surprising.

Response: Thank you, we indeed do not see the entire set of the regulatory proteins, including CaM. We reworded this sentence, highlighting the context of the natively preserved RyR1 lattice. It reads:
"To bridge this gap, we determined the structures of RyR1 by single-particle cryo-EM (SPA) and subtomogram averaging (StA) from cryo-ET data in its native membrane environment, within the receptor lattices to define the structural basis of coupled gating." (now lines 93 onwards)

We also added the following clarification to the Discussion to explicitly state which regulators were not visualized and why:

"Our analysis provided the most native high-resolution structures of RyR1 to date and revealed the details of the mechanism of ligand-modulated activation. While our preparations contained few putative T-tubules, similar to our previous report[22], they were rare, and the reconstructions did not have T-tubule membranes and DHPR; therefore, our analysis does not explain the voltage sensitivity of the EC-coupling machinery. Furthermore, calmodulin, the important

regulator of RyR1, was absent in all our conditions, while some auxiliary regulators: calsequestrin, triadin, and junctin were not visualized, likely due to their dynamic binding behavior….” (new lines 466 onwards).

Line 97: “We show that these lattice interactions constrain the channel's activation pathway to a rotation-dominant motion…” Please add “in plane” before “rotation-dominant motion” for clarity.

Response: Thank you, we updated the text: “We show that these lattice interactions constrain the channel's activation pathway to an in-plane rotation-dominant motion…”

Line 143: “Activating ligands shifted populations predominantly to the “Up” conformation”. This part is confusing.
1) Looking at Figure S13, it appears that these follow the same directions as published structures, i.e. Apo which has FlexionTilt of 2-5 and activation brings to a FlexionTilt around 0 in actMix. Thus going to a smaller value should be Down.
2) Up would be surprising considering the consensus among all other published structures of purified RyR, where the effect of Ca2+ moves the shell in the “Down” direction. It would be unlikely that the membrane (far away from the Ca+ binding site) would induce an opposite effect to that of Ca2+. Is it possible that Up and Down is defined relative to each condition, instead of all the dataset?

Response: Thank you for pointing this out.

First, we realized that the naming of the angles as FlexionRot and FlexionTilt is less intuitive for readers and decided to update the notation to “In-plane rotation” and “cytosolic shell tilt”. We give credit to the original reports of Montserrat Samso’s group and describe their calculation the methods section.

We followed the Angle definition as in Steele & Samso, JSB, 2019, in which downward motion of the cytosolic shell corresponds to lower angle values (termed FlexionAngle and cytosolic shell tilt in our study). “Up” and “Down” are indeed defined relative to the consensus reconstruction within each dataset (and within each functional-state subset where applicable), not as absolute labels across all datasets. Therefore, an “Up” class in one dataset can correspond to a lower absolute tilt value than a “Down” class in another dataset.

We added the explanation to the main text to remove the ambiguity and changed the order of the sentences, so the updated text in lines 190 onwards now reads:

“Cytoplasmic shells exhibit a wide range of motion in all states of the receptor; therefore, for each of the states in this study, we obtained additional classes, typically resulting in the shell position being either “Up” or “Down”, termed relatively to the consensus reconstruction for each of the functional states (Table S1). In all datasets, the consensus reconstructions from all particles represented the major class (Figs. S12 – S13). The “Down” conformation was predominant in the ActCa dataset (85.4% of particles, Table S1), while the activating ligands

have shifted particle populations predominantly to the "Up" conformation: 72.5% and 56.7% of particles for the Primed and Activated states, respectively (Table S1). The ActMix-ET dataset yielded both “Down” and “Up” conformations in addition to the consensus reconstruction, while 67% of particles were “Up” in the Ryanodine dataset (Table S1).”

3) the angle was calculated between the axis and the vertical, while normally this angle is considered with respect to the horizontal plane. This would yield opposite results.

Response: We thank the reviewer for this comment and have provided a detailed explanation below.
We followed the convention for the flexion angle for our measurements, as suggested in (Steele & Samso, JSB, 2019). In our implementation, we compute the angle relative to the Z-axis and then subtract from 90°, which yields the equivalent angle relative to the horizontal membrane plane, while preserving the published sign convention.

We expanded the method section (part of Analysis of structures, second paragraph). It reads:

“To calculate the cytosolic shell tilt (FlexionTilt) and in-plane rotation (FlexionRot) angles, the center of mass was defined for each model with the *define centroid* command. The Z and X axes were then defined from the center with *define axis*. The axis for the cytosolic shell was defined from the residue number 348 Cα atom to the residue number 984 Cα atom of chain A, to match the definition in the previous reports[17,30]. The residue number 984 was chosen instead of the original number 1,052 because of some modeling discrepancies between some earlier and later models in this less resolved region. Cytosolic shell tilt was calculated as the angle of this axis relative to the Z- axis, followed by subtracting the calculated value from 90 degrees. The subtraction from 90 degrees effectively gives the angle relative to the horizontal membrane plane. The sign of the resulting angle was determined from the Flexion-axis Z-vector sign (positive if the end-point is higher than the start-point along Z, negative if the end-point is lower than the start-point along Z). Because all models were positioned upside-down, the sign was inverted to match the flexion angle convention. The resulting values for the cytosolic shell tilt angle closely match the previously reported values[17,30] (see Table S5 for comparison). In-plane rotation was calculated as the angle of the shell axis relative to the X- axis, followed by subtracting the calculated value from 90 degrees. The sign of the resulting angle was determined from the Flexion-axis X-vector sign (positive if the end-point is higher than the start-point along X, negative if the end-point is lower than the start-point along X). As the X-axis is arbitraty, depending on the in-plane position of a model, it can also be alternatively defined as the axis between residue number 71 Cδ atom of chain B and residue number 71 Cδ atom of chain D.” (line 843 and onwards)

We verified that our FlexionTilt values match the trend and approximate values reported in the prior (Steele & Samso, JSB, 2019; Nayak & Samso, eLife, 2022) literature for comparable models;

We added an additional Table S5 with the measurements to the supplementary material.

Table S5. Comparison of the cytosolic shell tilt angle values measured in this study with the flexion angle values available from the literature.

Model (PDB ID)	flexion angle, reported previously (reference)	Cytosolic shell tilt, this study
5tal	-3.8, estimated (JSB, 2019)	-4.1
5tb0	1.7 estimated (JSB, 2019)	1.57
7tdj	-3.6 (eLife, 2022)	-3.52
7tdi	-4.4 (eLife, 2022)	-4.51
7tdk	-5.6 (eLife, 2022)	-5.44
7tdh	-5.1 (eLife, 2022)	-5.53
7k0t	-2.2 (eLife, 2022)	-2.64

4) Flexion Rot also depends on the X axis which is not well defined.

Response: Indeed, the in-plane X-axis can be defined arbitrarily because it depends on a model's orientation.

We now provide an explicit operational definition for the X-axis within our coordinate system and also provide an alternative definition based on fixed residues in our Apo/EGTA-SPA model.
We added the note to the methods: "As the X-axis is arbitrary, depending on the in-plane position of a model, it can also be defined as the axis between residue number 71 Cδ atom of chain B and residue number 71 Cδ atom of chain D" (line 861 onwards, also see the reply to your previous point)

We also added schematics of the X and Z axes and the cytosolic shell tilt/in-plane rotation definitions to the Supplementary Information to improve clarity for readers (Figure S16).

5) The reviewer recommends reconsidering the FlexionTilt angle with respect to the horizontal plane as in other works, and to provide a schematics of the X / Z axes, and the Flexion Tilt / FlexionRot axes, potentially in Fig. S13.

Response: We thank the reviewer for this recommendation. The cytosolic shell tilt (FlexionTilt) angle is already aligned with the previous reports, to which we refer in the results and the methods section "Analysis of structures". We validated that the previous reports result in similar values according to this convention, it is shown in the new Table S5.

We expanded the Methods section to include all the aforementioned details of the FlexionTilt and FlexionRot angle calculations and updated the text, see also part of the reply to your point 3.

As you suggested, we added the schematics of the Z and X axes, as well as the cytosolic shell tilt (FlexionTilt) and in-plane rotation (FlexionRot) axes, in Figure S16 (a-c). The other part of Figure S16, with the cytosolic shell domain movements upon functional state transitions, has been moved into a new Figure S17 for clarity.

Line 147: “In the EGTA-ET dataset, we also observed some variability in the EF1&2 hand domain conformation (Fig. S10) as well as its unexpected orientation relative to the S2S3 helical bundle domain of the neighbouring protomer (Fig. S11).” Please explain in which way the orientation is unexpected; this is not obvious from Fig. S10. Also, please comment how the orientations compare with respect to these described in Ref #17.

Response: We thank the reviewer for this suggestion and have now added the more detailed explanation, which reads:

“Unlike other datasets where the EF1&2 hand domain remains stable across subclasses, the EGTA-ET dataset revealed significant subclass-specific conformational variability in this region (Fig. S14). Structural similarity analysis of the EF1&2 hand domain using multidimensional scaling (see methods) relative to the CSol domain (Fig. S14i) or the neighboring S2S3′ bundle (Fig. S15j) consistently placed all EGTA-ET models outside the established Apo cluster, closer to canonical activated and inactivated states (Fig. S15a-h, j). Because inactivated conformations are reported to form salt bridges across this interface (Glu4075–Arg4736 and Lys4101–Asp4730), we measured the corresponding Cα-Cα distances. This also showed a higher similarity of the EF1&2 hand domain in the Apo-states in membranes to the canonical open and inactivated states of purified receptors (Fig. S15i). ” (lines 204 onwards)

Lines 198-199” In those studies, activation was associated with an extensive ~5.7° downward tilt and a ~5.1° rotation14”. Please double check that this is actually mentioned in the reference.

Response: Thank you, it is correct, it was not explicitly mentioned in the original references, and we calculated the values from the deposited atomic models. We modified the text as:

“Calculations based on the reported structures showed that the activation was associated with an extensive ~5.7° downward tilt and a ~5.1° rotation[3]...”

Lines 199-202: “In contrast, our analysis of RyR1 structures in native membranes revealed a cumulative in-plane rotation of ~6.3° during activation (Fig. 2A–F, Fig. S12, S12A-D)”. 1) Figure S12 does not show in plane angles. 2) There is not so much contrast: 6.3 degrees is not that different from 5.1 degrees found by others.

Response: Thank you, indeed. Fig. S12 (which is now Fig. S23) shows side views, so we have now referenced it elsewhere:

“In turn, activation-induced rearrangements within the TMD and activation core appear to be absorbed in the SR by deformations of the BSol itself rather than by a rigid-body tilt of the whole shell (Fig. S22a-d, S23).”

While the difference in the rotation angle was not large, the tilting difference was substantial. We rewrote the sentence:

“Our analysis of RyR1 structures in native membranes revealed a significantly lower total downward tilt of ~2.8° accompanied by a higher cumulative in-plane rotation of ~6.3° during activation (Fig. 2a–f, S17).”

Lines 204-209: “During priming, the RyR1 cytosolic shell tilts by ~2.8° and rotates by ~3.9° (Fig. S13B-C), while full activation is characterized primarily by additional rotation of ~2.4° without further significant tilt (Fig. S13D). In contrast, purified RyR1 structures exhibit greater cumulative tilting of ~4.5° during priming, and an additional ~1.1° tilt upon activation. Thus, the total difference in cumulative tilting between purified and native RyR1 channels is ~3°, emphasizing that native environment constraints substantially limit the vertical domain movements (Fig. 2G).”
1) Please explain where the 3 degrees comes from -the cumulative (in plane + vertical) rotation is 10.8 degrees for purified protein and 9.1 for protein within the membrane = 1.7 degrees difference = ~10% difference, which would be less dramatic.

Response: We thank the reviewer for this comment and provide a detailed explanation below.
We do not sum in-plane rotation and vertical tilt into a single metric, because these are distinct motions that are reported separately. Here, “cumulative tilt/rotation” refers to the total change in Tilt/rotation across the sequence of state transitions (state 1 → state 2 → state 3 …). Below are the explicit tilt calculations:

For the SR structures:

Apo (Apo5, EGTA-SPA, consensus) -> Primed with Activating [free Ca^{2+}] (ActCa1, consensus) = 3.18º - 0.9º = **2.28º tilt**;

Primed with Activating [free Ca^{2+}] (ActCa1, consensus) -> Primed with ActMix (ActMix1, consensus) = 0.9º - 0.36º = **0.54º tilt**;

Primed with ActMix (ActMix1, consensus) -> Activated with ActMix (ActMix3, consensus) =0.36º - 0.44º = **-0.08º tilt**;

Cumulative tilt in the SR structures: **2.28º + 0.54º + -0.08º = 2.74º**;

For the purified structures:

Apo (5TB0, consensus) -> Primed with Activating [free Ca^{2+}] (5T15, consensus) = 1.57° - -1.73° = **3.3°**;

Primed with Activating [free Ca^{2+}] (5T15, consensus) -> Primed with ActMix (5TAQ, consensus) = -1.73° - -2.98° = **1.25°**;

Primed with ActMix (5TAQ, consensus) -> Activated with ActMIx (5TAL, consensus) = -2.98° - -4.10° = **1.12°**;

Cumulative tilt in the Purified structures: **3.3° + 1.25° + 1.12° = 5.67°**;

Difference in the cumulative tilt upon functional state transitions between the SR and the Purified structures is therefore **5.67° - 2.74° = 2.93°**;

We rounded all the angles in the main text, so the final difference became **"~3°"**;
Due to the large size of the receptor, these are large difference (by a factor of ~5.7/~2.7 =~2.1x) in the cumulative tilting between the structures in SR and the purified structures.
To estimate the cumulative in-plane rotation differences, the same calculations were done for the in-plane rotation angles.

We updated the corresponding section:
"In contrast, purified RyR1 structures exhibit greater overall conformational change with a cumulative tilting of ~4.5° during priming, and an additional ~1.1° tilt upon activation[14]. The total difference in cumulative tilting between purified and native RyR1 channels is ~3°, emphasizing that native environment constraints limit the vertical domain movements (Fig. 2g). " (line 277 onwards).

Figure S15 B-D. Dimension 1 and 2 are arbitrary; It would help reproducibility if this information could be displayed with more context, showing the directionality of such changes.

Response: Thank you, dimensions 1 and 2 in our scatter plots originate from linear multidimensional analysis, and the distances between the points have physical meaning relative to each other, but not on the absolute scale. We illustrated conformational changes between the states with vector fields in our Figures 2 and S23.

For further reproducibility, we uploaded the code to produce these scatters to the GitHub of our group: https://github.com/KudryashevLab/RMSDistance. This should make it accessible and usable by other researchers.

The figure would greatly benefit if purified RyRs and SR-RyRs were in different panels.

Response: Thank you for this suggestion! We revised Fig. S15 (which is now Fig. S24) for clarity and separated structures from the SR and the purified structures on the plots, shown side

by side. Figure S24 now shows the cytosolic shell tilt vs. in-plane rotation plot (top) as well as the CSol displacement relative to the TMD (bottom). Fig. S25 shows EF1&2 hand domain displacement plots, and Fig. S26 shows the salt-bridging residue distances between the EF1&2 hand domain and the S2S3' helical bundle.

Methods, line 446. "1 mM CaCl2 and EGTA stock solutions were added to the sample to a final concentration of 30 uM Ca2+ determined with Maxchelator". It is not clear if Mg2+ and ACP were included in the Maxchelator calculation but they should be, as ACP binds Ca2+ and Mg2+ reducing their free concentrations.
Also, free Mg2+ should be stated: both free Ca2+ and Mg2+ concentration are critical to determine the state of the channel, and need to be considered for comparison with the existing structures.

Response: Thank you, we expanded this part of the Sample Preparation Methods section. It reads:

"EGTA was added to the Apo-state sample aliquots to a final concentration of 5 mM. $CaCl_2$ stock solution was added to the ActCa and ActMix sample aliquots to reach the target concentration of 30 μM free Ca^{2+}, according to the MaxChelator[4], with free Mg^{2+} and added ACP (if applicable) taken into account. The free Mg^{2+} concentration was approximately 10-15 μM, as it was mostly sequestered by the pyrophosphate, and even lower at approximately 0.4 μM in the 2mM ACP-containing samples. ACP and caffeine were added to the mixture at the final concentrations of 2 mM and 5 mM, respectively, and incubated at 4 °C for 30 min. After the addition of ryanodine to a final concentration of 5 μM, the sample was incubated overnight at 4 °C to ensure complete binding." (lines 564 onwards)

Table S1: Structures/classes %: please add the % of the different subclasses.

Response: We thank the reviewer for this suggestion and added the particle percentages in Table S1 for each class.

Figure S11: Please indicate the identity of the 4 residues of interest at least in one of the panels.

We thank the reviewer for this suggestion and added the 4 residue captions in panel (a) in what is now Figure S15.

It is difficult to extract the main idea from the different pairwise comparisons; it could help to use the same "reference" reconstruction for the different panels.

Response: We thank the reviewer for this suggestion and added the Apo1/Consensus to each of the panels a–h as a reference structure in what is now Figure S15.

In addition, large distance differences do not reflect well in the panels. For example in panel E, the E4075-R4736 distance does not seem to be 8 Å different as indicated in panel I. Perhaps it is the perspective but it would be worth to double check.

Response: Thanks for pointing this out. We reoriented the models so that the distances between the domains are more apparent.

Figure S15: Very interesting figure, but the names of the atomic models are superimposed in some of the panels, becoming illegible. Also, please add the definition of the color coding.

Response: Thank you for pointing this out. We revised this figure and added the color legend.

As mentioned above, we revised Figure S24 (used to be Fig. S15) for clarity and separated the SR and the purified structures on the plots, shown side by side. Fig. S24 now shows the cytosolic shell tilt vs. in-plane rotation plot as well as the CSol displacement relative to the TMD. Fig. S25 shows EF1&2 hand domain displacement plots, and Fig. S26 shows the salt-bridging residue distances between the EF1&2 hand domain and the S2S3' helical bundle.

Figure S17: Please indicate the residue corresponding to wt and mutation

Response: Thank you for this suggestion. We changed the captions for each point mutation to contain both the WT and the substitution amino acid identity. It is now Fig. S31.

Figure S18: Probably this is the same flowchart for one of the ActMix datasets, please indicate so in the legend. Also, please describe the "Particle Subtraction" in the legend or in the text

Response: Thank you for this suggestion. We added the StA flowchart for the ActMix-ET dataset (Fig. S34), substantially revised the StA flowchart for the EGTA-ET dataset (Fig. S33), and expanded the "Cryo-ET Data Processing" Methods section to include all the necessary details of what was done.

Reviewer #2 (Remarks to the Author):

The work presents a valuable investigation into the high-resolution structures of RyR1 in native membranes using cryo-EM STA and SPA, successfully revealed the conformational transitions underlying RyR1 channel opening. The capture of multiple functional states along the activation pathway provides important insights into the mechanism of synchronized Ca^{2+} release in skeletal muscle, which represents a notable advance in understanding RyR1 biology.

Response: We thank the reviewer for the positive assessment and for noting the significance of the study.

However, several critical limitations—related to technical clarity, sample validity, structural resolution, and data presentation—undermine the robustness of key conclusions, particularly

regarding inter-RyR1 interfaces, dimer function, and physiological relevance. Below is a detailed expansion of concerns:

1. Line 104, the phrase "following the previous reports" is misleading and uninformative. A core strength of this study is its achievement of high-resolution RyR1 structures in native membranes—yet the manuscript fails to highlight the key technical improvements that enabled this advance, which is critical for readers to evaluate and build upon the work.

Response: Thank you very much. We agree that the manuscript contains substantial cutting-edge structural data. We greatly expanded on its description in the methods sections: "Cryo-ET Data Processing" and "SPA Data Processing".

We updated and added multiple supplementary material figures: S9, S10, S28, S29, S33, and S34.

And added a new paragraph to the discussion section:

"Our ability to obtain high-resolution structures from cryo-ET reflected an end-to-end improvement of throughput: we increased the number of RyR1 particles per tomogram by optimizing sample enrichment and grid preparation, collected more tomograms per session using automated acquisition with PACE-tomo[31], and accelerated downstream analysis through streamlined data processing implemented in tomoBEAR[44], as well as sensitive template matching in PyTOM-TM[32,33]. These improvements raised our effective yield to roughly ~70 particles per tomogram (about ~13k particles per dataset), exceeding earlier reports that recovered on the order of ~30–60 particles per tomogram[25,31]. In parallel, we used single-particle analysis when vesicles could be imaged in sufficiently thin ice; as expected, SPA generally produced slightly higher-resolution reconstructions, due to the larger particle numbers contributing to the reconstructions. The two approaches were complementary: cryo-ET clearly showed the RyR1 lattice, while SPA increased particle statistics, and together they enabled structural characterization, including determination of RyR1 dimer structures." (lines 440 onwards)

And added "cryo-ET" to the abstract to show it as a prominent method.

While the Methods section outlines protocols, the Results section lacks clear discussion of how the authors optimized sample preparation, cryo-EM data collection, or data processing to surpass the resolution of prior in situ RyR1 studies. Without this context, the novelty of the methodological approach is obscured.

Response: Thank you, we are very happy to elaborate on the technical aspects of our paper. We added more description of the updates to our sample preparation workflows to the methods section:
"We changed the discontinuous sucrose gradient layover pattern to enrich the heavy SR fraction in the target bands, improving the amount of RyR1 particles per field of view in our data."

And: “We optimized the sample concentration and volume applied to the grid as well as the blot time, to ensure the proper ice gradient along the hole for optimal data collection.”

To the results section:
“We isolated junctional SR microsomes from rabbit skeletal muscle and examined RyR1 arrays by cryo-ET (Fig. 1A), following the previous reports[22,23,25,26], and performed high-resolution imaging of the SR vesicles in 3D by cryo-ET and in 2D for single particle cryo-EM. “ (lines 108 onwards)

And added a new paragraph in the discussion section, see our reply to your previous comment.

2. Line 106, Misrepresentation of “checkerboard” organization. The claim that isolated SR microsomes exhibit a RyR1 lattice “closely resembling their native ‘checkerboard’ organization in intact muscle triads” is unsupported. Mammalian skeletal muscle triads feature RyR1 arranged in two tightly packed, uniformly oriented rows on the SR, with consistent alignment of all tetramers. In contrast, Fig. S1 shows no evidence of this ordered two-row arrangement; instead, RyR1 orientation appears heterogeneous and packing is irregular. This discrepancy strongly suggests post-isolation rearrangement of RyR1 tetramers.
If the authors insist on “closely resembling” native triads, they must provide side-by-side comparative diagrams of their lattice versus published intact triad structures and quantify key parameters (e.g., inter-RyR distance, tetramer orientation).

Response: We thank the reviewer for this comment. We refer to the local organization of the dimer contacts between the receptors. We clarified the text.

The protocol for the extraction of the SR vesicles directly from the native tissue does not contain detergent and retains the native lipid environment and protein-protein interactions. However, during homogenization and centrifugation, SR membranes fragment into vesicles, which disrupts the large-scale, ordered two-row clustering but preserves the local inter-channel arrangement. This is what we see in our data: large arrays on the SR surface appear less regular, but many channels retain the native corner-to-corner contacts (Figure S1), closely resembling the contacts reported by Xu et al (Science Advances, 2024, DOI: 10.1126/sciadv.adl1126) in intact muscle, as we show in our new Figure S30.

The modified section now reads as follows:
“While the linear two-row organization of the receptors in the muscle was perturbed, the local order was preserved with the corner-to-corner contacts clearly visible (Fig. S1). The corner-to-corner contacts were formed via bridging-solenoid BSol domains, recapitulating the physiological arrangement of RyR1.” (at the beginning of the results section, lines 111 onwards).

We added two figures with the workflows for structural characterization for dimers, S28 and S29, that also show a very clear preference of neighbouring receptors to form contacts, representing their native arrangement. We refer to them in the section “A BSol-mediated interface forms the structural basis for RyR1 coupling.”

3. Potential membrane curvature artifacts. Native junctional SR in triads is a highly curved, narrow structure optimized for DHPR-RyR1 coupling. However, the SR sample in this study appear relatively flat.
Membrane curvature directly influences protein conformation and lattice organization—flattening could alter RyR1's cytosolic domain orientation or inter-channel contacts. The authors must address whether this curvature change impacts structural conclusions.

Response: Thank you very much. We provided a detailed comparison of the RyR1 dimer from the isolated SR vesicles and the native triad junction in Fig. S30; we refer to it in the section: "A BSol-mediated interface forms the structural basis for RyR1 coupling". Our analysis demonstrates an overall preservation of the inter-channel contacts. When it comes to a single channel, the local membrane shape around its TMD appears to be preserved in both cases, independent of the membrane curvature (new figure S32). The same pattern of the local membrane flattening around the TMD was reported in the RyR1 structure from the triad junctions with T-Tubules (Fig.3a in Nayak and Samsó. eLife 2022;0:e75568). Even higher membrane curvature was observed in the proteoliposome reconstituted RyR1 (Melville et al, Structure 2022 30(1):172-180.e3), and it had no profound effect on the protein conformation. nor the local flattening. Therefore, the SR membrane curvature in our case does not create major artifacts in the cytosolic shell domains orientation.

We added Figure S32, which compares variations of membrane curvatures from our data with the native triad junctions, which shows that there is a local curvature in the structure in the tussie (Xu et al, Science Advances, 2024), similar to the one observed by us.

We added a statement in the Discussion:

"The loss of the T-tubules has also induced a higher SR membrane curvature, varying between datasets (Fig. S32). The local membrane curvature in the channel vicinity appeared to be preserved, displaying a characteristic membrane flattening in the TMD insertion area (Fig. S32)." (lines 471 onwards)

3. Line 108 references "Using a combination of SPA and StA," but the manuscript provides no justification for this hybrid approach or practical details of its implementation. Readers need to understand why was a combination necessary?

We thank the reviewer for this question. The detailed explanation is provided below.
Despite all the recent advances in cryo-ET that allowed us to achieve the resolution of up to 4 Å in our subtomogram averages, the technique is generally still lower in throughput compared to the standard cryo-EM imaging. It's particularly hard to obtain the open-pore state from cryo-ET and StA, as typically less than 50% of particles have open pores even under the fully activating conditions. For the set of ~13k particles we get from our data, that would mean that there are not enough open-pore particles to isolate by 3D classification and reconstruct to high enough resolution.

We optimized the sample and specimen preparation as outlined in the answer above, which allowed us to skip the stage tilting and record SPA data. With more particles and thinner ice, we obtained more RyR1 particles than with cryo-ET and resolved the open-pore state as well as some other functional states at higher resolution with ordered lipids and ligand densities.

Nevertheless, cryo-ET was essential to visualize the arrangement of the receptors in a lattice and was instrumental in obtaining the first structure of the RyR1 dimer, which was then used as the initial template for the SPA dimer structures. It is also a highlight for the broader structural biology community that cryo-ET and StA can achieve maps of comparable resolution and quality even with smaller particle numbers, as each particle in StA contains signal from multiple viewing directions. Another methodologically interesting aspect of the cryo-ET data in our study is that it provides a hardware benchmark: a Gatan K3 DED with a Gatan BioQuantum K3 EF was used for one dataset, and a Falcon4i DED with a SelectrisX EF was used for another on the same sample with similar particle numbers.

We have described it in the text:
"These improvements raised our effective yield to ~70 particles per tomogram (about ~13k particles per dataset), exceeding earlier reports that recovered on the order of ~30–60 particles per tomogram[25,31]. In parallel, we used single-particle analysis when vesicles could be imaged in sufficiently thin ice; as expected, SPA generally produced higher-resolution reconstructions, due to the larger particle numbers contributing to the reconstructions. The two approaches were complementary: cryo-ET clearly showed the RyR1 lattice, while SPA increased particle statistics, and together they enabled structural characterization, including determination of RyR1 dimer structures." (line 445 onwards)

4. Vague references to "previously reported structures". The manuscript repeatedly claims similarities to prior RyR1 structures without quantitative or visual validation. For all such claims, the authors must include side-by-side structural alignments (maybe in supplementary figures) of their models with the referenced structures, along with RMSD values for key domains.

Response: We thank the reviewer for this suggestion. We added Figures. S18, S19, S20, and S21 with superimposed models and per-domain RMSDs for each consensus model reported in this study. The models chosen for comparisons correspond to the same functional states. The RMSD values are reported in the table format, and each panel additionally has the model colored and represented (varying cartoon thickness) according to the RMSD values.

These figures will facilitate readers' assessment of the differences between our atomic models and previously determined ones. These newly added figures are also referenced in the text:

"Comparison of the structures or RyR1 in SR membranes and the purified receptors quantified by a per-domain RMSD calculation confirms this trend (Figs. S18-21)." (lines 278 onwards)

5.Line 137, inadequate characterization of “Up,” “Down,” and “Tight” states. The structural differences between these states (Fig. S9) are visually indiscernible due to the lack of local zoomed-in panels highlighting the most dynamic regions.

Response: We thank the reviewer for noting this. The “Up” and “Down” states are always denominated for each functional state. This means that each class is compared to the consensus reconstruction within each dataset (or functional state in case of the ActMix-SPA dataset that yielded two functional states).

We have now expanded the original Fig. S9 (which is now Fig. S11) into three figures with the zoomed-in panels on the two cytosolic shell parts: Fig. S11, S12, and S13. These new zoom-in panels will make the inter-class differences clearer.

We updated the text:
“Our analysis further revealed conformational variability of RyR1 cytosolic shell in native membranes, particularly in the Apo state, classifiable into discrete "Up," "Down," and "Tight" states based on positions of BSol domains (Fig. S11).”

And:
“In all datasets, the consensus reconstructions from all particles represented the major class (Figs. S12 – S13).” (lines 194 onwards)

Additionally, the Methods mention centroid-based angle measurements, but the Results provide no clear diagrams of how these angles were calculated (e.g., axes definitions, reference points for “Up” vs. “Down” orientations).

Response: We thank the reviewer for this recommendation and have added the schematics of the Z and X axes, as well as the cytosolic shell tilt and in-plane rotation axes, in Fig. S16 (a-c).

6. Fig. S9–S11: Text labels (e.g., domain names, state designations) are difficult to read.

Response: We thank the reviewer for this note, and we updated the Figures. S9 – S11, which are now Figsures S11-S13, S14, and S15.

Distance matrix plots (e.g., in Fig. S15): Protein identifiers are jumbled, making it impossible to trace arrangement of different states. Fig. S15: Protein labels in the FlexionTilt/FlexionRot plot are illegible, preventing readers from verifying trends in cytosolic shell motion. These figures must be revised for clarity, with standardized font sizes, simplified layouts, and clear legends.

Response: We thank the reviewer for this note, and we have revised Fig. S15 (now Figs. S24-26) for clarity and separated the SR and the Purified structures on the plots, shown side by side. Fig. S24 now shows the cytosolic shell tilt vs in-plane rotation plot as well as the CSol displacement relative to the TMD. Fig. S25 shows EF1&2 hand domain displacement plots, and

Fig. S26 shows the salt-bridging residue distances between the EF1&2 hand domain and the S2S3' helical bundle.

7. Line 249, the authors report RyR1 dimers in five functional states, but the manuscript does not explain why dimer resolution is significantly lower than monomer resolution (e.g., is it due to conformational heterogeneity of the interface, or insufficient particle numbers?). This is critical because contact area measurements rely on these low-resolution models—readers need to know if such analyses are statistically robust.

Response: Thank you very much. Indeed, the resolution of the dimer structures is lower than of individual receptors, which we believe is the case for 3 reasons: 1) lower particle number; 2) higher flexibility associated with the larger macromolecule, which also changes upon activation, making it naturally more flexible than some other complexes, such as i.e. Ribosome large and small subutits; and 3) potential loss of some dimer particles during data processing.

We modified the text accordingly,

a) We added the particle numbers to Table S1. Even with our highest number of particles of ~37.000-33.000, the resolution was limited by flexibility.
b) We modified the Results "A BSol-mediated interface forms the structural basis for RyR1 coupling "section: "The resolutions of the dimer maps ranged from 11.7 to 26 Å (Table S1), limited by the particle number and the conformational heterogeneity of the flexible interface." (lines 324 onwards)
c) Updated the results section: "Notably, a fraction of RyR1 particles were lost during cryo-ET data processing due to technical limitations, resulting in only a fraction of channels being engaged in the interaction with neighbours in case of EGTA-ET and ActMix-ET datasets." (lines 333 onwards)
d) The dimer subsets represent locally preserved native array packaging and showed functional coupling (Figure 4a-f). Although the reconstructions of dimers are at a lower resolution (11.7–26 Å), the interface is characterized by rigid-body docking high-resolution monomer models, derived from focused refinement of the same datasets, into the dimer density. Because the dimer maps provide sufficient detail for unambiguous model fitting, we believe that the resulting contact area measurements and interface analyses are structurally well-supported.

We updated the discussion:
"Our dimer maps allowed us to unambiguously dock high-resolution monomer models from the same datasets, revealing clear corner-to-corner interactions mediated primarily by the BSol domain loops (residues 2982–2992, 3111–3116, 3220–3236) and helices (residues 2955–2980, 3085–3110)." (lines 342 onwards)

8. Table S1 provides no information on the number of particles used for dimer reconstruction. If most particles were discarded during 3D classification, the remaining subset may not be representative of physiological RyR1 interactions.

Response: Thank you, we updated Table S1 with the particle counts used for each dimer reconstruction. In the SPA datasets, where we optimized the ice layer thickness to be lower, the signal is higher, and dimer engagement is substantial, reaching up to ~89% in the ActCa dataset. The Apo-state dimer structure resembled the *in situ* dimer structures also in the apo-state from FIB-milled native triad junctions in mouse skeletal muscle (EMD-37094). Our dimer structure shows high agreement with the native architecture, with a peak inter-tetramer distance of 31.5 nm, compared to 31.1 nm native, and a center-to-center angle of 17° compared to 17.9° native. The slight difference in out-of-plane tilt (5.1° vs. 1.8°) likely reflects the higher membrane curvature in our isolated SR vesicles following T-tubule loss (Fig. S32).

While the absolute number of dimer-engaged particles is lower in the cryo-ET datasets - 256 for EGTA-ET, this is possibly due to the thicker ice and a higher ratio of particle loss during subtomogram classification. To confirm that these subsets are representative and not random, we generated 3D heatmaps of the nearest-neighbor localization (Figures S28a, S29a). These heatmaps show four distinct, C4-symmetry-related peaks corresponding to preferred corner-to-corner contacts, providing structural evidence for a non-random, preferred orientation between RYR1 in our sample.

We updated the results section:
"To understand the mechanism constraining the channel's tilt and mediating inter-receptor communication, we analyzed the interactions between adjacent receptors by determining the structures of RyR1-RyR1 dimers in five different functional states (Fig. 3, Fig. S16, Table S1). For this, we took advantage of the local order of the RyR1-RyR1 lattice (Fig.S1). The resolutions of the dimers maps ranged from 11.7 to 26 Å (Table S1), limited by the particle number and the conformational heterogeneity of the flexible interface. As these structures are the product of averaging, our reconstructions specifically represent the most homogeneous and well-ordered lattice units, as the more flexible dimer arrangements were excluded during 3D classification. Ordered contact preservation was particularly high in the SPA datasets, reaching 89% in the ActCa dataset (Table S1). In cryo-ET datasets, the estimated contact ratio was lower; we analyzed 3D neighbour heatmaps demonstrating the preferred arrangements of neighbours (Figs. S28a, S29a). These heatmaps showed four C4-symmetry-related peaks corresponding to native corner-to-corner interactions. The Apo-state dimer structure was further validated by direct comparison with the in situ dimer structures from FIB-milled native triad junctions (EMD-37094, Xu et al, Sci. Adv.10,eadl1126 (2024)). The Inter-receptor center-to-center distance was measured at 31.5 nm, closely matching the 31 nm average seen in intact triads (Fig. S30). Similarly, the center-to-center angle was 17°, aligning with the native 18.3° angle. This analysis shows that the RyR1 lattice tends to be locally preserved in purified SR microsomes even in the absence of the T-Tubule membrane. Our dimer maps allowed us to unambiguously dock high-resolution monomer models from the same datasets, revealing clear corner-to-corner interactions mediated primarily by the BSol domain loops (residues 2982–2992, 3111–3116, 3220–3236) and helices (residues 2955–2980, 3085–3110)." (lines 321 onwards)

9. Fig. S1 suggests heterogeneous inter-RyR1 contacts in isolated SR microsomes. The authors must address whether this heterogeneity can be resolved for dimer reconstruction and how it impacts conclusions about "coupled gating."

Response: Thank you, as an average structure, it represents the most common conformation. We highlighted it in the previous reply:
"As these structures are the product of averaging, our reconstructions specifically represent the most homogeneous and well-ordered lattice units, as the more flexible dimer arrangements were excluded during 3D classification.".

The conclusions about the coupled gating were drawn from the statistical analysis performed on the clean homogenous dimers, so the heterogeneous packing does not influence them.

We generally agree with the reviewer that obtaining higher resolution maps of dimers with more conformational intermediates would be interesting and should be possible by collecting more data. However, this will take major effort, and we believe that it goes beyond the scope of the current manuscript.

10. Missing structural determination workflows. The Methods section does not include schematic workflows for STA, SPA, and dimer reconstruction. These diagrams are essential for readers to follow how raw tomograms/ micrographs were processed into final models (e.g., particle picking, classification steps, refinement iterations).

Response: We thank the reviewer for this suggestion. We added detailed schematic workflows for the StA, SPA, and dimer reconstruction pipelines to the Supplementary Information (Figs. S28b, S29b, S33, S34, S35, and S36). These diagrams describe the complete processing path, including initial particle picking, 3D classification, and refinement iterations, into the final high-resolution models. Correspondingly, the Methods sections for "Cryo-ET Data Processing" and "SPA Data Processing" have been significantly expanded to provide an exhaustive description of these workflows and the specific technical optimizations used to achieve high-resolution reconstructions.

11. Fig. 4H, the schematic of coupled gating is overly simplistic and fails to integrate key structural details. It should be revised to include: (1) Visual cues for conformational changes, (2) Labels for key domains, and (3) A timeline of events.

Response: Thank you! The scheme in Figure 4h illustrates the proposed model for coupled gating based on the rotation-dominating mechanism that we described in our manuscript. We revised the scheme in Figure 4h to label the RyR1-RyR1 interfaces and the fact that every second RyR1 in the lattice is associated with four DHPRs. We intentionally simplified the scheme as the details of the interactions and the domain movements are now described in the main text and supplementary information in fine detail. This scheme is a conceptual illustration of how counterclockwise rotation within a lattice releases neighbour interfaces throughout the RyR1 lattice.
